# Supplementary material for: AI-guided pipeline for protein–protein interaction drug discovery identifies a SARS-CoV-2 inhibitor
Source: Mol Syst Biol. 2024 Mar 11;20(4):428–57. doi: 10.1038/s44320-024-00019-8 (PMC10987651; doi:10.1038/s44320-024-00019-8)
Supplement: Supplementary file 1 — Appendix [file 44320_2024_19_MOESM1_ESM.pdf]

APPENDIX

Table of Content:

**Appendix Figure**	**page**
Appendix Figure S1	2
Appendix Figure S2	4
Appendix Figure S3	5
Appendix Figure S4	6
Appendix Figure S5	7
Appendix Figure S6	9
Appendix Figure S7	10
Appendix Figure S8	11

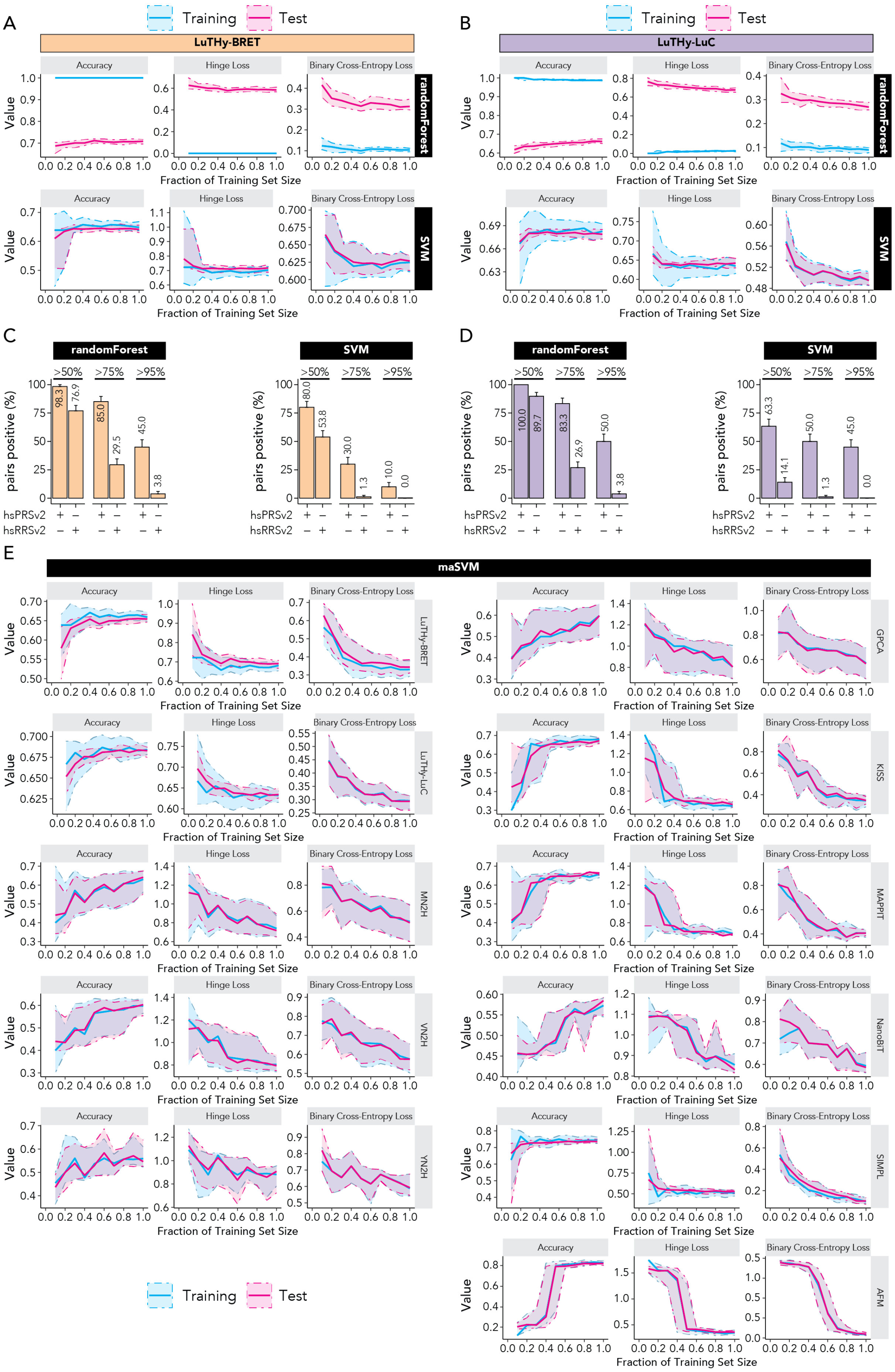
A
Training
Test
LuTHy-BRET
Accuracy
Hinge Loss
Binary Cross-Entropy Loss
randomForest
SVM
Value
Fraction of Training Set Size
B
LuTHy-LuC
C
D
randomForest
SVM
>50%
>75%
>95%
pairs positive (%)
hsPRSv2
hsRRSv2
98.3
76.9
85.0
29.5
45.0
3.8
80.0
53.8
30.0
1.3
10.0
0.0
100.0
89.7
83.3
26.9
50.0
3.8
63.3
14.1
50.0
1.3
45.0
0.0
E
maSVM
LuTHy-BRET
LuTHy-LuC
MN2H
VN2H
YN2H
GPCA
KISS
MAPPIT
NanoBiT
SIMPL
AFM

Appendix Figure S1 (related to Figure 1). Learning performance over fraction of training set size.

(**A,B**) Training curves for random forest or support vector machine models trained in 50 ensembles on fractions of the training set consisting in total of 182 hsPRS-v2 and 182 hsRRS-v2 reference set protein pair configurations sampled randomly from the total number of 1104 available reference set protein paris. The 704 protein pairs not used for training were used as the test set. Training curves show 'Accuracy', 'Hinge Loss' and 'Binary Cross-Entropy Loss' (y-axis) are plotted against the fraction of training set size (x-axis) for (**A**) LuTHy-BRET and (**B**) LuTHy-LuC. The continuous line represents the median and the band the interquartile range. (**C,D**) Bar plots showing the fraction of hsPRS-AF and hsRRS-AF protein pairs that scored above classifier probabilities of 50%, 75% and 95%. (**E**) Training curves as in (A,B) for the maSVM algorithm for the LuTHy-BRET (e=50, i=5, C=1; 182 hsPRS-v2 & 182 hsRRS-v2 protein pairs) LuTHy-LuC (e=50, i=5, C=1, 182 hsPRS-v2 and 182 hsRRS-v2 protein pairs), mN2H (e=100, i=10, C=0.1, 46 hsPRS-v2 and 46 hsRRS-v2 protein pairs), vN2H (e=100, i=10, C=0.1, 46 hsPRS-v2 and 46 hsRRS-v2 protein pairs), yN2H (e=25, i=5, C=0.1, 110 hsPRS-v2 and 110 hsRRS-v2 protein pairs), GPCA (e=100, i=10, C=0.1, 46 hsPRS-v2 and 46 hsRRS-v2 protein pairs), KISS (e=25, i=10, C=0.1, 53 hsPRS-v2 and 53 hsRRS-v2 protein pairs), MAPPIT (e=25, i=5, C=0.1, 54 hsPRS-v2 and 54 hsRRS-v2 protein pairs), NanoBiT (e=25, i=5, C=0.1, 110 hsPRS-v2 and 110 hsRRS-v2 protein pairs, SIMPL (e=25, i=10, C=0.1, 76 hsPRS-v2 and 76 hsRRS-v2 protein pairs) and AFM (e=100, i=5, C=0.01, 86 hsPRS-v2 and 86 hsRRS-v2 protein pairs) assays.

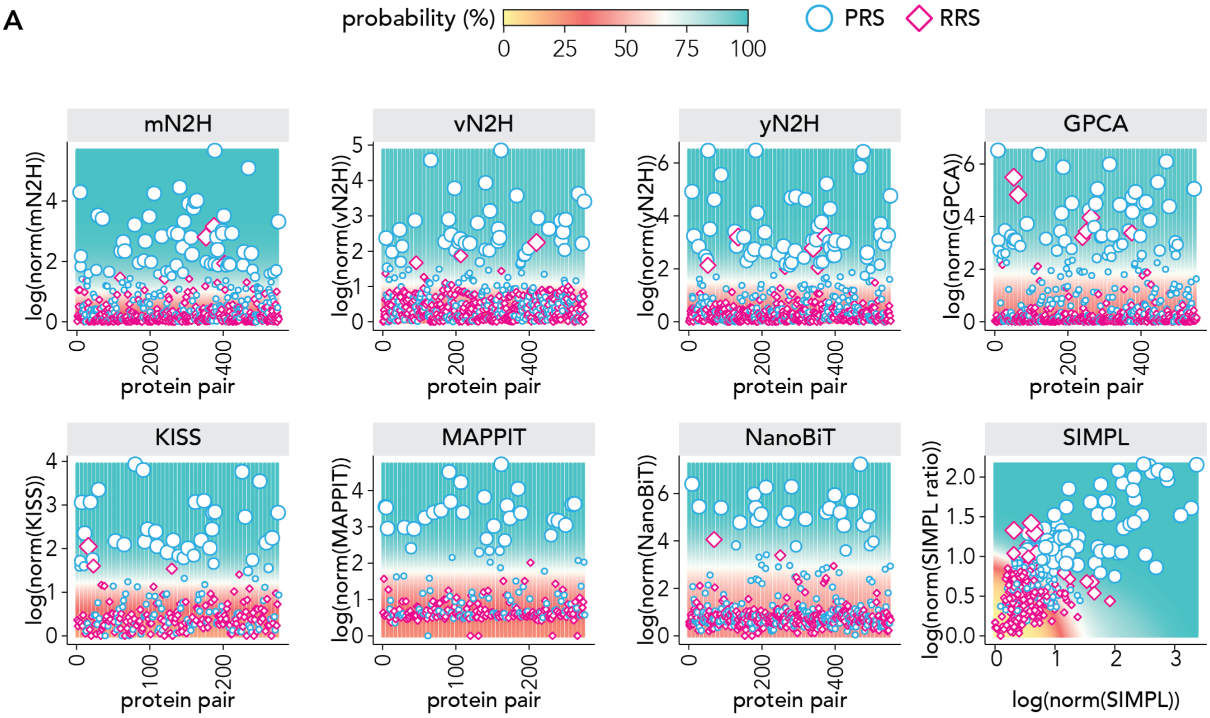

Appendix Figure S2 (related to Figure 1). maSVM decision boundaries for eight quantitative PPI assay variants.

(**A**) Scatter plots for eight quantitative PPI assay variants showing the number of protein pairs (x-axis) against their respective log transformed and robust scaler normalized interaction scores (y-axis) for hsPRS-v2 (blue) and hsRRS-v2 (magenta) protein pairs. Average classifier probability from the maSVM models is displayed as the size of the data points and as a colored grid in the background. The maSVM algorithm for the SIMPL assay was trained on the robust scaler normalized SIMPL interaction score (SIMPL, x-axis) and the robust scaler normalized ratio between SIMPL interaction score and bait expression (SIMPL ratio, y-axis) using the published data from Yao et al (Yao et al, 2020). For the SIMPL assay, the x- and y-axis are log transformed. Note that the SIMPL assay was benchmarked against 88 positive proteins pairs derived from the hsPRS-v1 (Venkatesan et al, 2009) and as a random reference set against “88 protein pairs with baits and preys selected from the PRS but used in combinations determined computationally to have low probability of interaction” (Yao et al, 2020). Data for all other assays is from Choi et al (Choi et al, 2019).

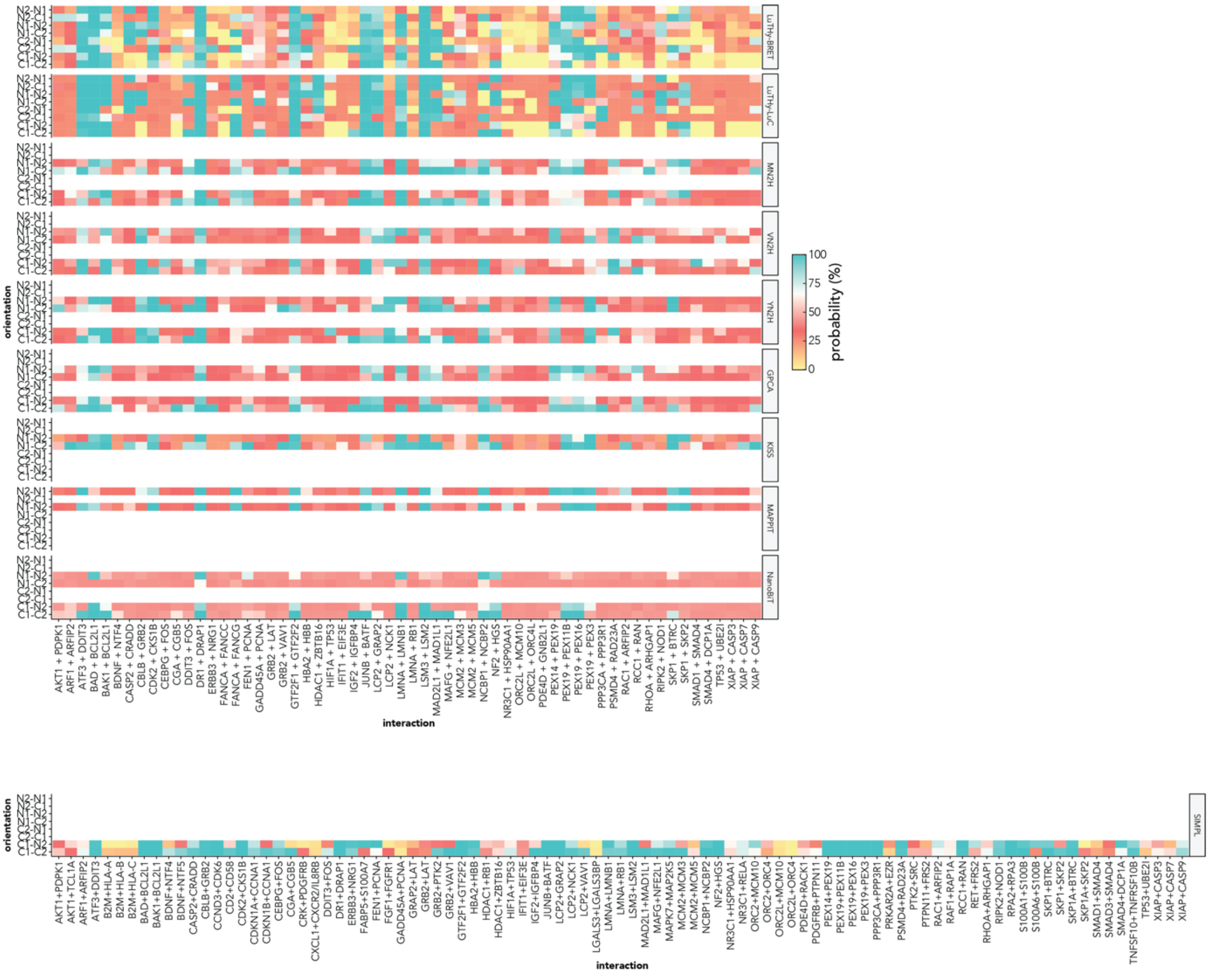

Appendix Figure S3 (related to Figure 1). Tagging configuration-specific classifier probabilities for positive reference interactions.

Heatmaps showing the tagging configuration-specific classifier probabilities for the assays benchmarked with hsPRS-v2 and for the positive reference protein pairs tested for the SIMPL assay (Yao et al, 2020). LuTHy data from this study; all other data from Choi et al. (Choi et al, 2019) and Yao et al. (Yao et al, 2020).

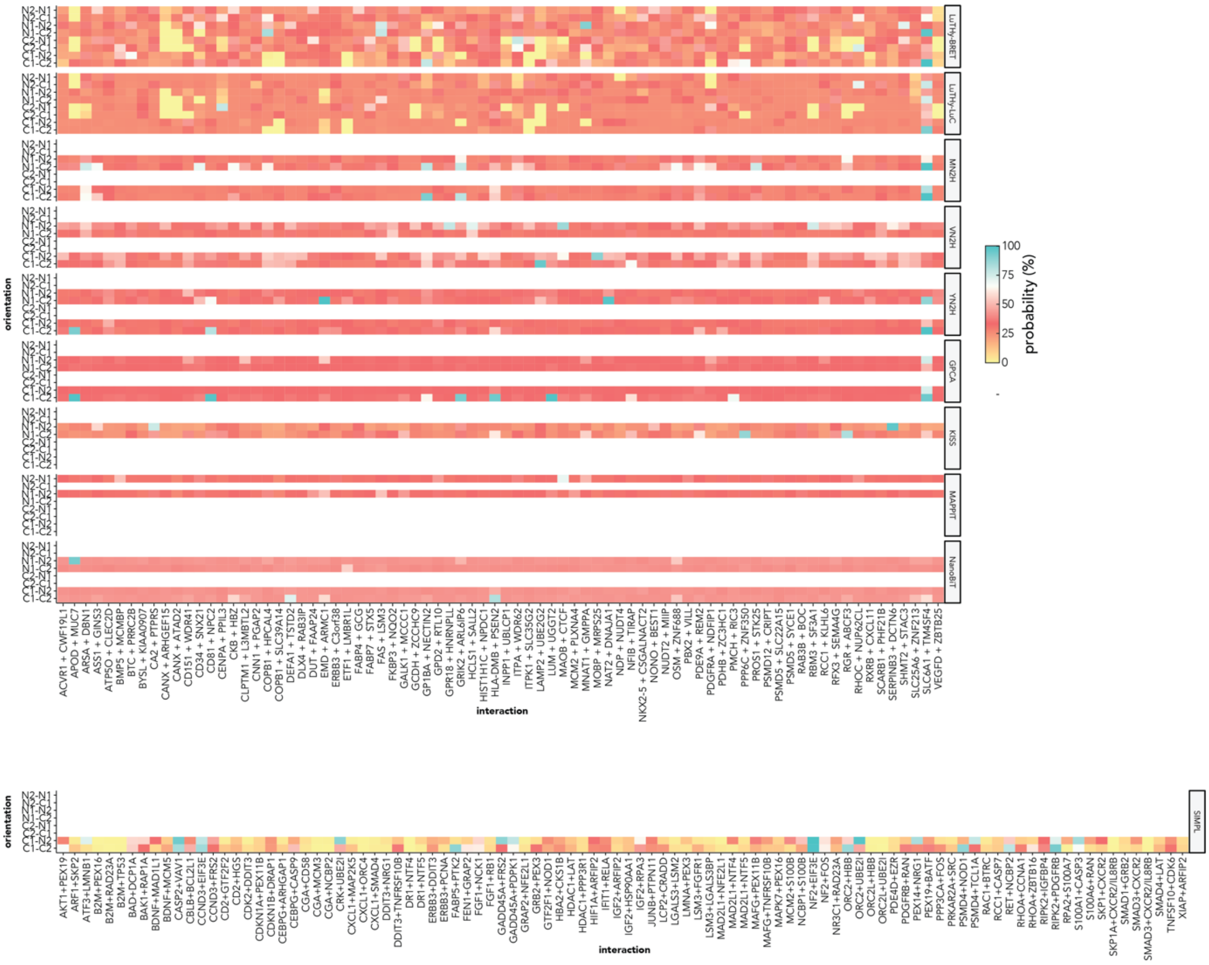

Appendix Figure S4 (related to Figure 1). Tagging configuration-specific classifier probabilities for random reference protein pairs.

Heatmaps showing the tagging configuration-specific classifier probabilities for the assays benchmarked with hsRRS-v2 and for the random reference protein pairs tested for the SIMPL assay (Yao et al, 2020). LuTHy data from this study; all other from Choi et al (Choi et al, 2019) and Yao et al (Yao et al, 2020).

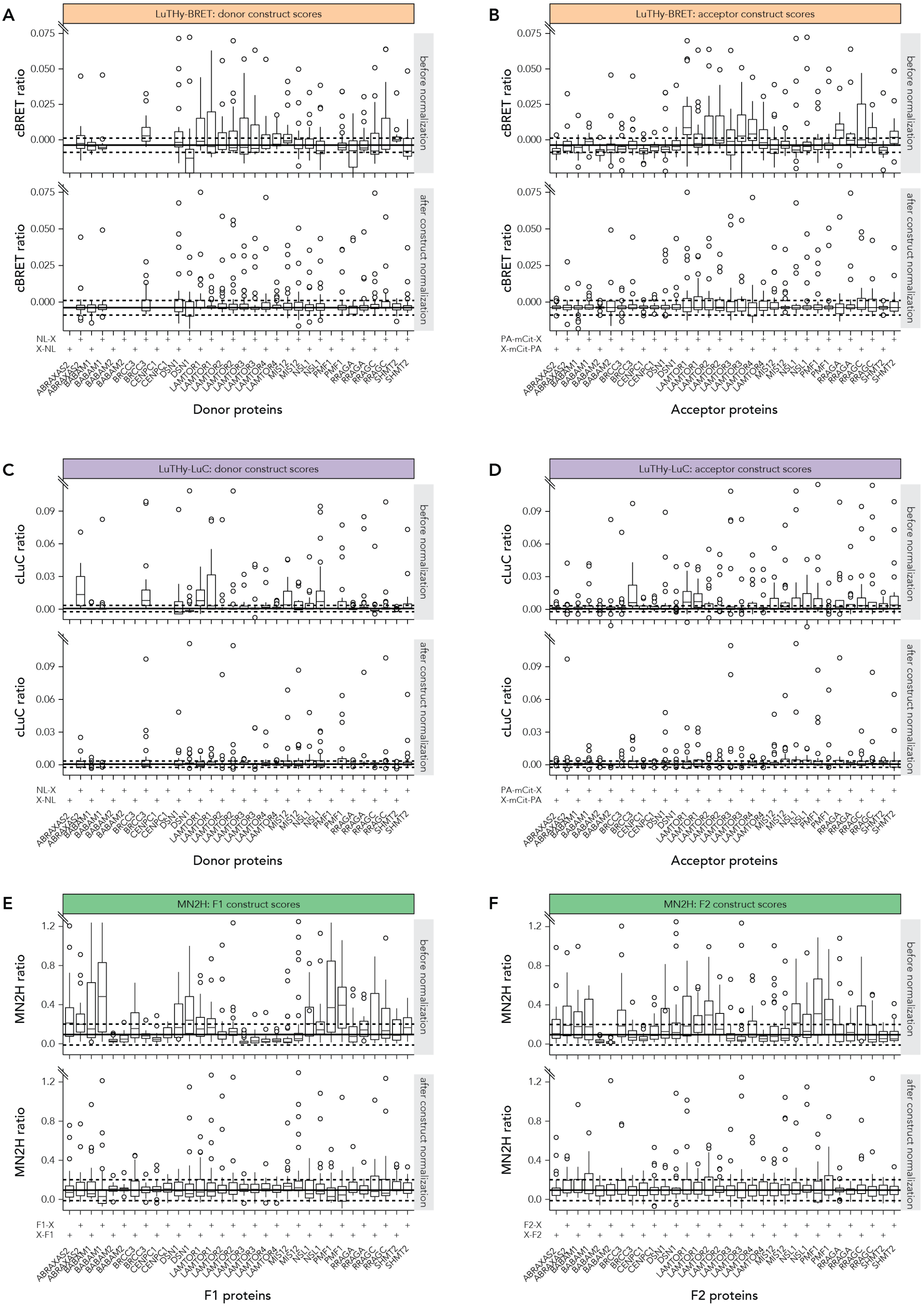
A
LuTHy-BRET: donor construct scores
cBRET ratio
before normalization
after construct normalization
NL-X
X-NL
Donor proteins
B
LuTHy-BRET: acceptor construct scores
PA-mCit-X
X-mCit-PA
Acceptor proteins
C
LuTHy-LuC: donor construct scores
cLuC ratio
D
LuTHy-LuC: acceptor construct scores
E
MN2H: F1 construct scores
MN2H ratio
F1-X
X-F1
F1 proteins
F
MN2H: F2 construct scores
F2-X
X-F2
F2 proteins
ABRAXAS2 ABRAXAS2 BABAM1 BABAM1 BABAM2 BABAM2 BRCC3 BRCC3 CENPC1 CENPC1 DSN1 DSN1 LAMTOR1 LAMTOR1 LAMTOR2 LAMTOR2 LAMTOR3 LAMTOR3 LAMTOR4 LAMTOR4 MIS12 MIS12 NSL1 NSL1 PMF1 PMF1 RRAGA RRAGA RRAGC RRAGC SHMT2 SHMT2

Appendix Figure S5 (related to Figure 3). Construct-specific robust scaler normalization for mapping multiprotein complexes.

(**A-F**) Boxplots of assay specific interaction scores before and after robust scaler normalization for the multiprotein complex proteins in all tagging configurations of donor and acceptor constructs. Boxplots display the constructs' median, lower and upper hinges the 25th and 75th percentiles, lower and upper whiskers extending from the hinges with 1.5x the interquartile range and outlier points beyond the end of the whiskers. The thick horizontal line indicates the median interaction score over all constructs of the multiprotein complex set and the dashed lines the respective IQR of the 25th and 75th quartiles. Note that the horizontal lines always refer to the median and IQR before normalization and that the range of the y-axis is limited to visualize all boxplots as well as the median and IQR, but high scoring protein pairs (outliers) are hidden. (**A**) cBRET ratios for donor constructs before (top) and after (bottom) robust scaler normalization. (**B**) cBRET ratios for acceptor constructs before (top) and after (bottom) robust scaler normalization. (**C**) cLuC ratios for donor constructs before (top) and after (bottom) robust scaler normalization. (**D**) cLuC ratios for acceptor constructs before (top) and after (bottom) robust scaler normalization. (**E**) mN2H ratios for F1 constructs before (top) and after (bottom) robust scaler normalization. (**F**) mN2H ratios for F2 constructs before (top) and after (bottom) robust scaler normalization.

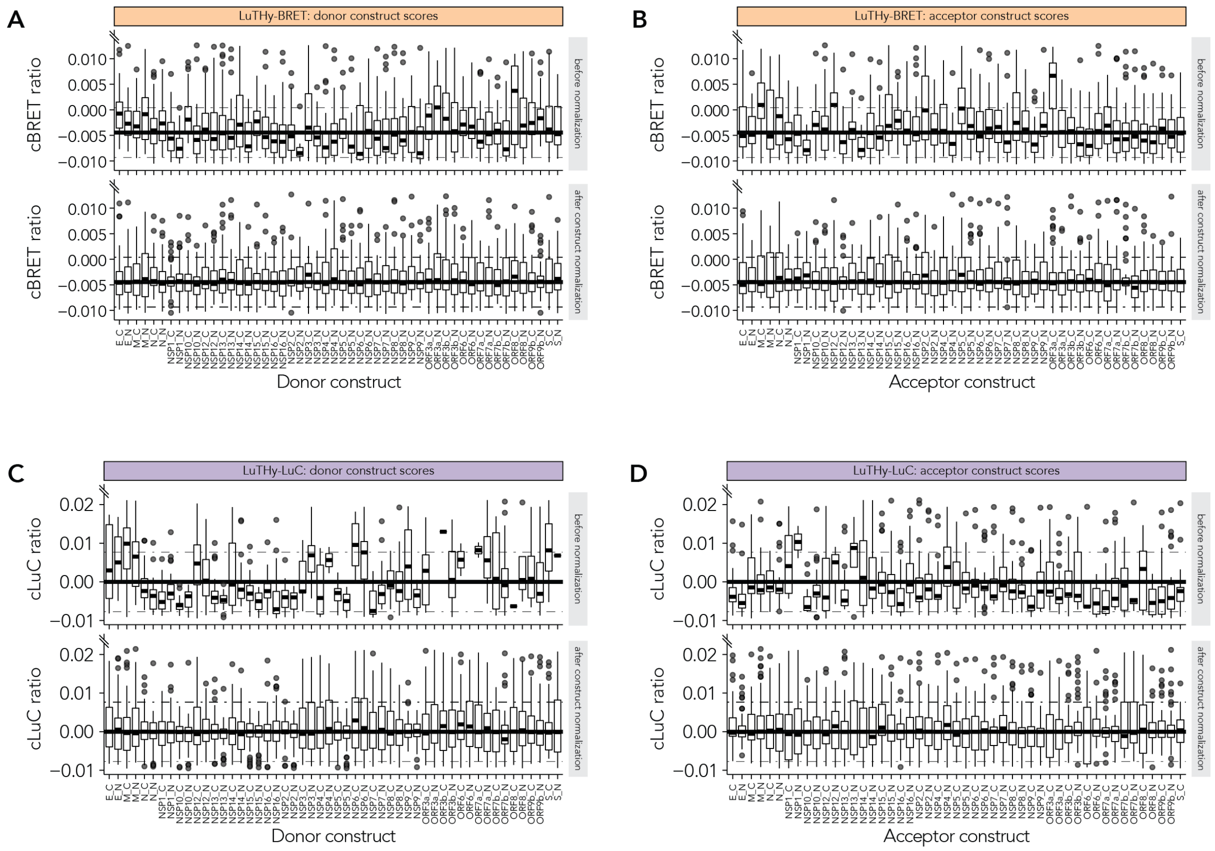

Appendix Figure S6 (related to Figure 4). Construct-specific robust scaler normalization for SARS-CoV-2 binary PPI mapping.

(**A-D**) Boxplots of LuTHy-BRET and LuTHy-LuC interaction scores before and after robust scaler normalization for the SARS-CoV-2 proteins in all tagging configurations of donor and acceptor constructs. Boxplots display the constructs' median, lower and upper hinges the 25th and 75th percentiles, lower and upper whiskers extending from the hinges with 1.5x the interquartile range and outlier points beyond the end of the whiskers. The thick horizontal line indicates the median interaction score over all constructs of the training (hsPRS-v2, hsRRS-v2, multiprotein complex) and test set (SARS-CoV-2) and the dashed lines the respective IQR of the 25th and 75th quartiles. Note that the horizontal lines always refer to the median and IQR before normalization and that the range of the y-axis is limited to visualize all boxplots as well as the median and IQR, but high scoring protein paris (outliers) are hidden. (**A**) cBRET ratios for donor constructs before (top) and after (bottom) robust scaler normalization. (**B**) cBRET ratios for acceptor constructs before (top) and after (bottom) robust scaler normalization. (**C**) cLuC ratios for donor constructs before (top) and after (bottom) robust scaler normalization. (**D**) cLuC ratios for acceptor constructs before (top) and after (bottom) robust scaler normalization.

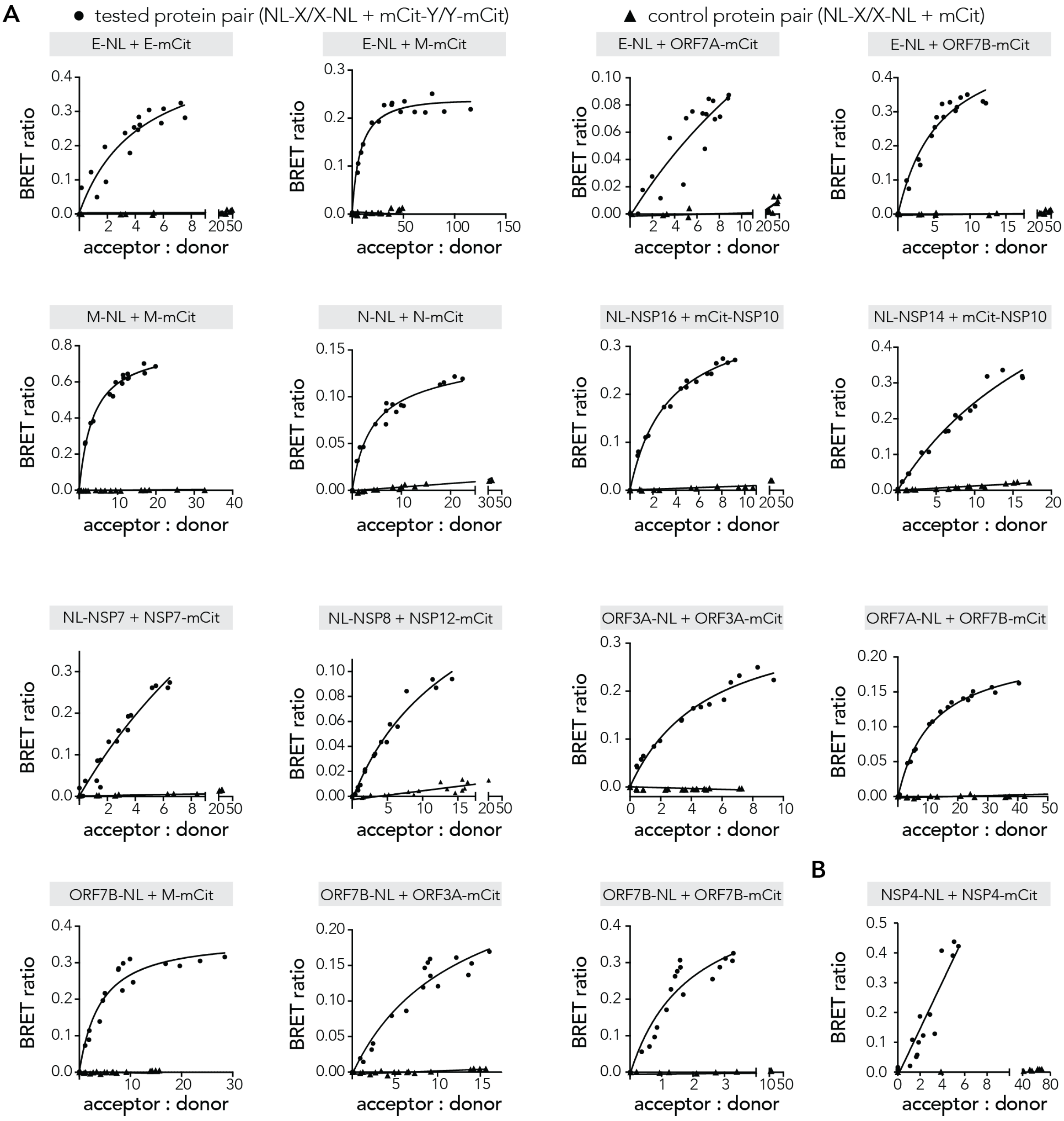

Appendix Figure S7 (related to Figure 4 and Figure EV3). LuTHy-BRET binding strengths for SARS CoV-2-AF protein complexes.

(**A**,**B**) Scatter plots showing LuTHy-BRET donor saturation curves with the acceptor to donor ratio (x-axis) plotted against the BRET ratio (y-axis) for 16 CoV-2-AF protein pairs with classifier probabilities >75%. A non-linear regression fit was performed using the ‘One site – Total and nonspecific binding’ in GraphPad Prism, using the results from the ‘NL-X/X-NL + mCit’ protein pair to subtract nonspecific binding in order to calculate the acceptor to donor ratios at half-maximal BRET ratios ($BRET_{50}$). $BRET_{50}$ values for protein pairs in (**A**) were used in Figure EV7F. (**B**) For the homodimer between NSP4-NSP4 the calculation of a $BRET_{50}$ failed due to the linear relation between acceptor : donor and the BRET ratio. The linear relation can be the result of an unspecific binding between the two proteins or because of a higher order oligomerization of the protein. Experiments were repeated twice accounting for n = 2 biological replicates, each containing 2 technical replicates.

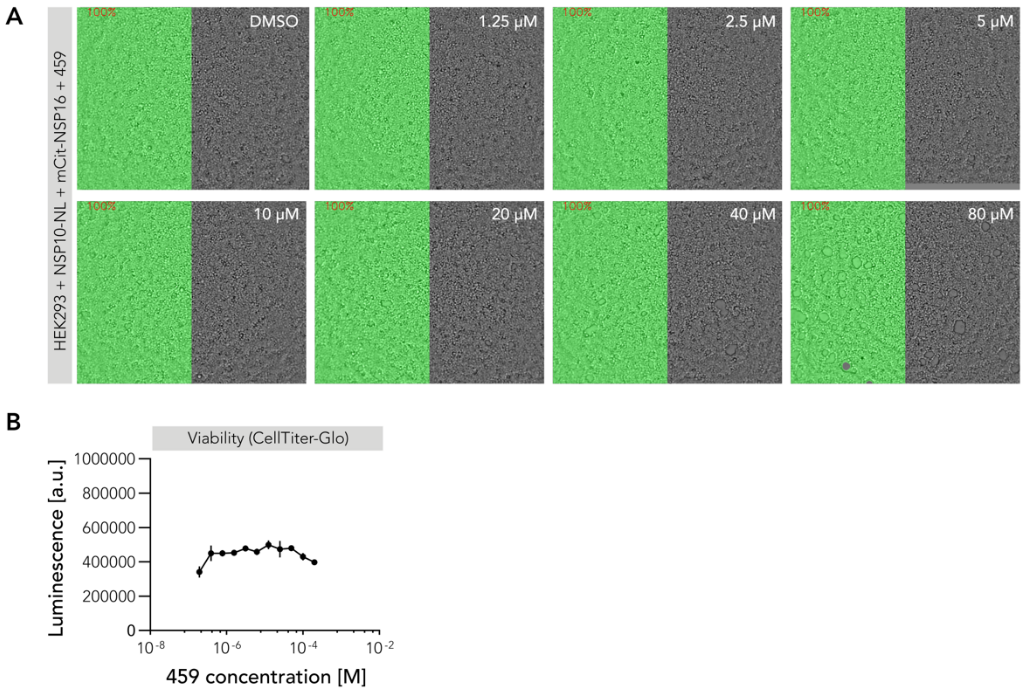

Appendix Figure S8 (related to Figure 6). HEK293 cell morphology, confluency and viability after compound treatment in LuTHy-BRET assay.

(**A**) Images from automated microscopy (20x magnification) showing cell morphology (right, gray images) and cell confluency (left, green images) after compound treatment. Cells were transfected with NSP10-NL and mCitrine (mCit)-NSP16 to quantify the interaction in living cells. Neither cell morphology nor cell confluency was influenced by treatment with up 80 µM of compound 459. (**B**) Cell viability after treatment with compound 459 in SARS-CoV-2 replication assays. Dots represent the average luminescence intensities of the CellTiter-Glo readout and error bars correspond to the standard deviations.
