# Supplementary material for: AI-guided pipeline for protein–protein interaction drug discovery identifies a SARS-CoV-2 inhibitor
Source: Mol Syst Biol. 2024 Mar 11;20(4):428–57. doi: 10.1038/s44320-024-00019-8 (PMC10987651; doi:10.1038/s44320-024-00019-8)
Supplement: Supplementary file 2 — Dataset EV1 [file 44320_2024_19_MOESM2_ESM.zip › Dataset EV1/hyperparameter_comparison_VN2H.html]

Hyperparameter optimization for VN2H predictions


# *Hyperparameter optimization* for **VN2H** predictions

#### Philipp Trepte

#### 2023-10-25

Hyperparameter comparison (e, i, C) for the maSVM algorithm trained
on the *reference* set

# Hyperarameters

## ensembleSize: 25

### iter: 1

#### C: 0.01

(**A**) Recovery training data, (**B**) ROC
curve training data

---

#### C: 0.1

(**A**) Recovery training data, (**B**) ROC
curve training data

---

#### C: 1

(**A**) Recovery training data, (**B**) ROC
curve training data

---

#### C: 10

(**A**) Recovery training data, (**B**) ROC
curve training data

---

### iter: 5

#### C: 0.01

(**A**) Recovery training data, (**B**) ROC
curve training data

---

#### C: 0.1

(**A**) Recovery training data, (**B**) ROC
curve training data

---

#### C: 1

(**A**) Recovery training data, (**B**) ROC
curve training data

---

##### C: 10

(**A**) Recovery training data, (**B**) ROC
curve training data

---

### iter: 10

#### C: 0.01

(**A**) Recovery training data, (**B**) ROC
curve training data

---

#### C: 0.1

(**A**) Recovery training data, (**B**) ROC
curve training data

---

#### C: 1

(**A**) Recovery training data, (**B**) ROC
curve training data

---

#### C: 10

(**A**) Recovery training data, (**B**) ROC
curve training data

---

## ensembleSize: 50

### iter: 1

#### C: 0.01

(**A**) Recovery training data, (**B**) ROC
curve training data

---

#### C: 0.1

(**A**) Recovery training data, (**B**) ROC
curve training data

---

#### C: 1

(**A**) Recovery training data, (**B**) ROC
curve training data

---

#### C: 10

(**A**) Recovery training data, (**B**) ROC
curve training data

---

### iter: 5

#### C: 0.01

(**A**) Recovery training data, (**B**) ROC
curve training data

---

#### C: 0.1

(**A**) Recovery training data, (**B**) ROC
curve training data

---

#### C: 1

(**A**) Recovery training data, (**B**) ROC
curve training data

---

##### C: 10

(**A**) Recovery training data, (**B**) ROC
curve training data

---

### iter: 10

#### C: 0.01

(**A**) Recovery training data, (**B**) ROC
curve training data

---

#### C: 0.1

(**A**) Recovery training data, (**B**) ROC
curve training data

---

#### C: 1

(**A**) Recovery training data, (**B**) ROC
curve training data

---

#### C: 10

(**A**) Recovery training data, (**B**) ROC
curve training data

---

## ensembleSize: 100

### iter: 1

#### C: 0.01

(**A**) Recovery training data, (**B**) ROC
curve training data

---

#### C: 0.1

(**A**) Recovery training data, (**B**) ROC
curve training data

---

#### C: 1

(**A**) Recovery training data, (**B**) ROC
curve training data

---

#### C: 10

(**A**) Recovery training data, (**B**) ROC
curve training data

---

### iter: 5

#### C: 0.01

(**A**) Recovery training data, (**B**) ROC
curve training data

---

#### C: 0.1

(**A**) Recovery training data, (**B**) ROC
curve training data

---

#### C: 1

(**A**) Recovery training data, (**B**) ROC
curve training data

---

#### C: 10

(**A**) Recovery training data, (**B**) ROC
curve training data

---

### iter: 10

#### C: 0.01

(**A**) Recovery training data, (**B**) ROC
curve training data

---

#### C: 0.1

(**A**) Recovery training data, (**B**) ROC
curve training data

---

#### C: 1

(**A**) Recovery training data, (**B**) ROC
curve training data

---

#### C: 10

(**A**) Recovery training data, (**B**) ROC
curve training data

---
