# Supplementary figures and images for: AI-guided pipeline for protein–protein interaction drug discovery identifies a SARS-CoV-2 inhibitor (part 1 of 2)
Source: Mol Syst Biol. 2024 Mar 11;20(4):428–57. doi: 10.1038/s44320-024-00019-8 (PMC10987651; doi:10.1038/s44320-024-00019-8)

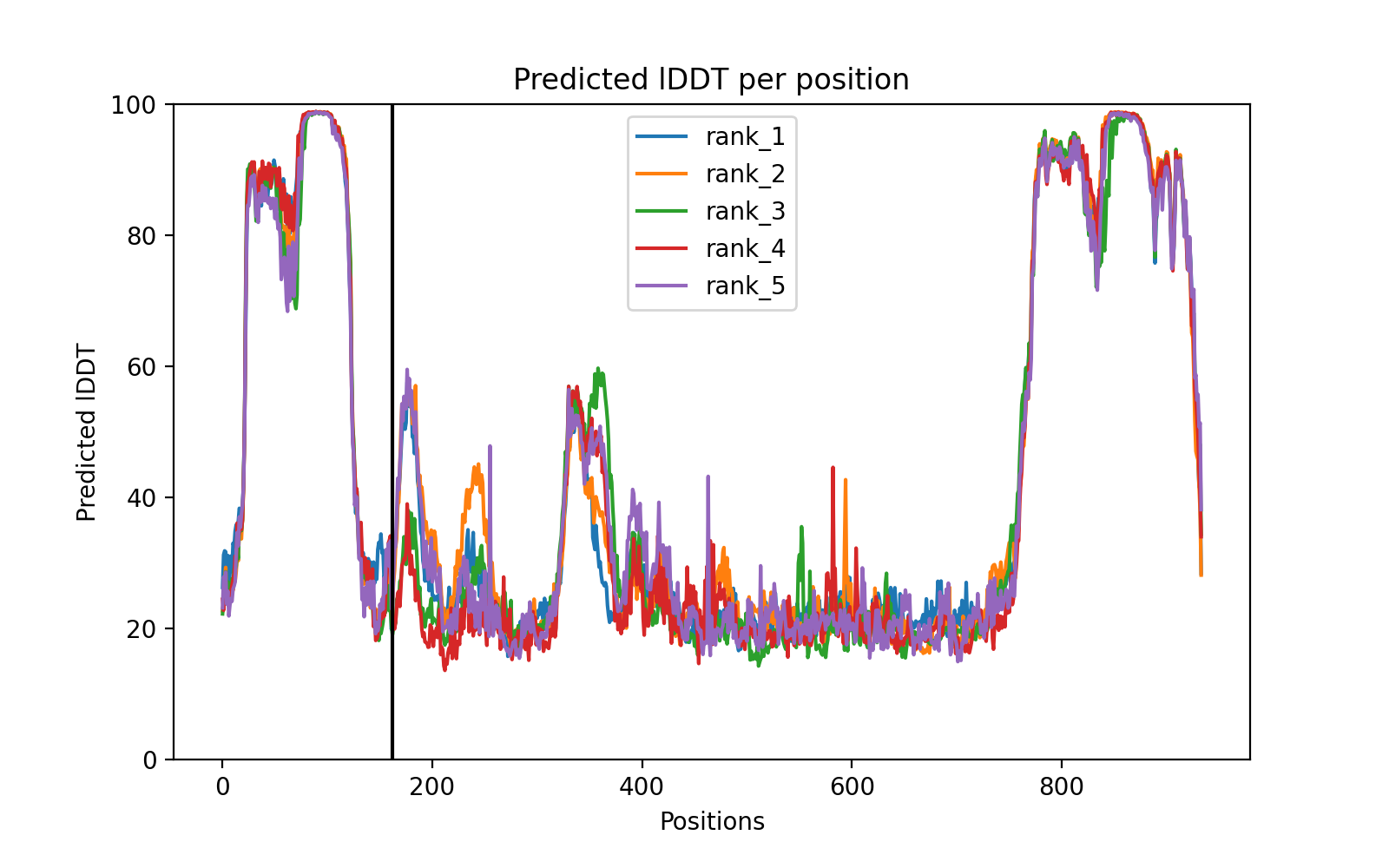

Supplement: Supplementary file 12 — Source Data Fig. 2 [file 44320_2024_19_MOESM12_ESM.zip › Source Data Figure EV3-EV4/ColabFold/MAFG_NFE2L1_plddt.png]

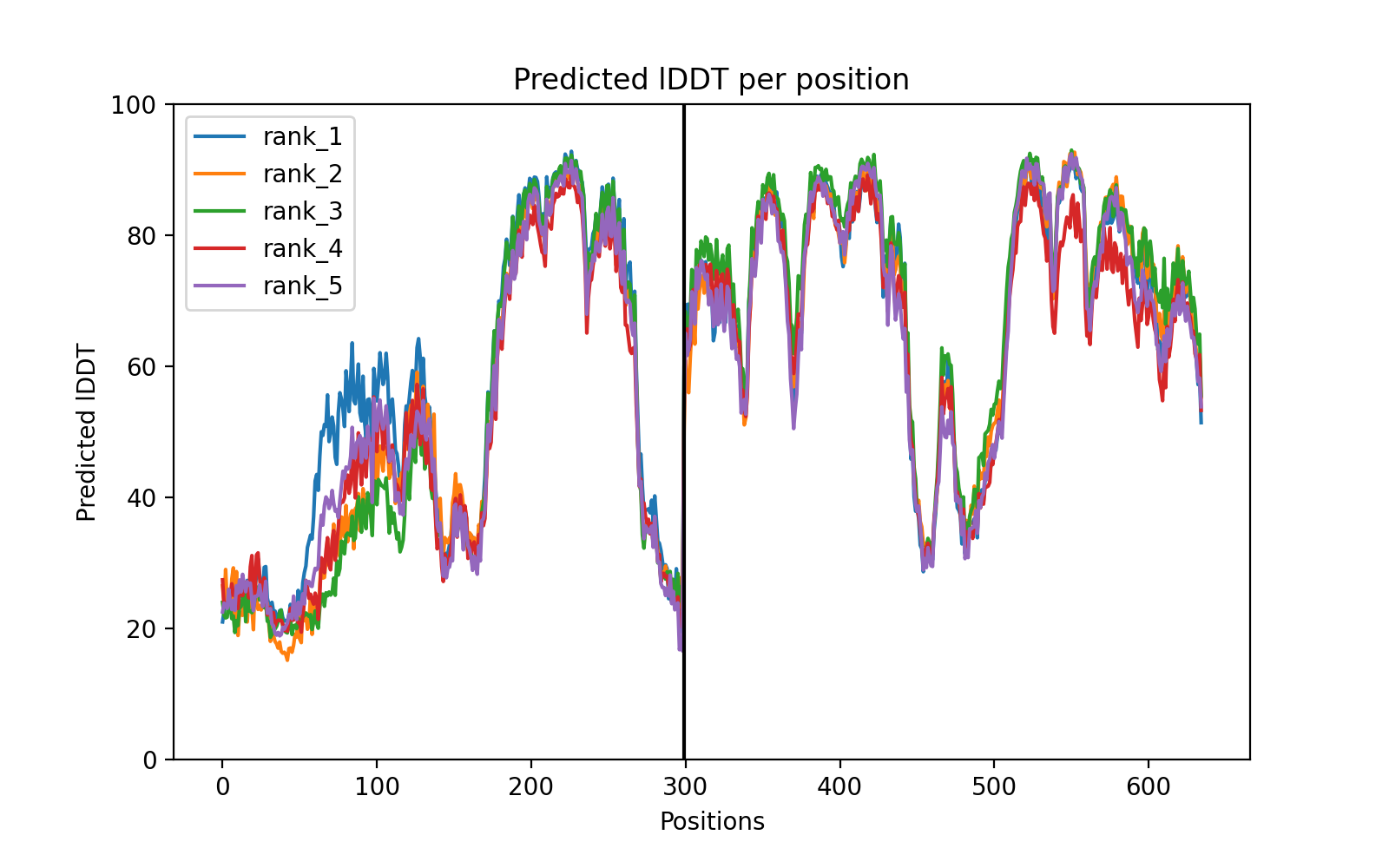

Supplement: Supplementary file 12 — Source Data Fig. 2 [file 44320_2024_19_MOESM12_ESM.zip › Source Data Figure EV3-EV4/ColabFold/PEX19_PEX16_plddt.png]

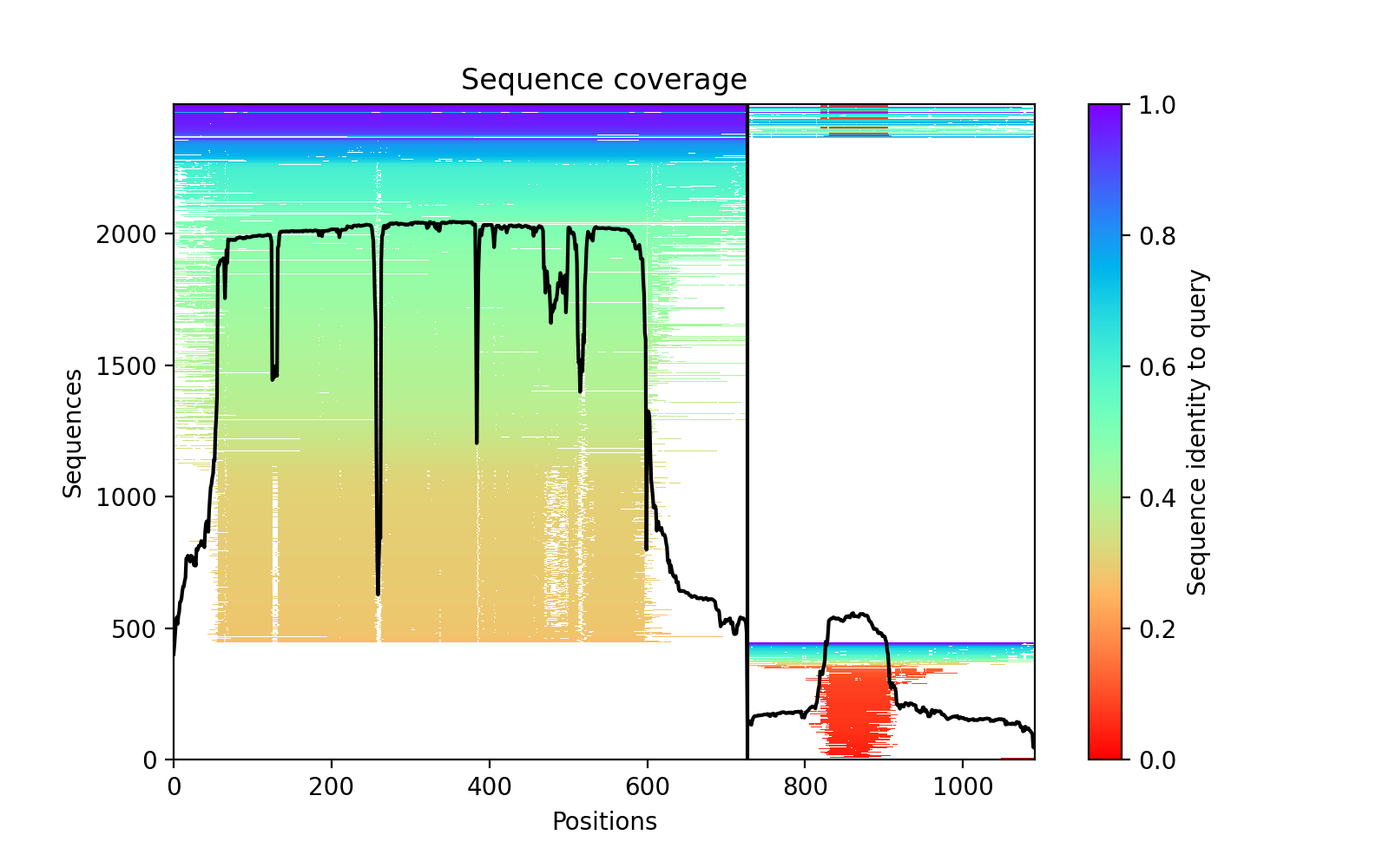

Supplement: Supplementary file 12 — Source Data Fig. 2 [file 44320_2024_19_MOESM12_ESM.zip › Source Data Figure EV3-EV4/ColabFold/GPD2_C22orf29_coverage.png]

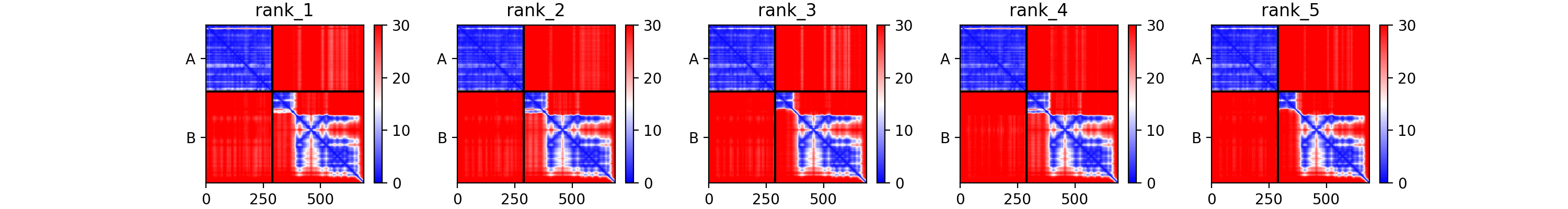

Supplement: Supplementary file 12 — Source Data Fig. 2 [file 44320_2024_19_MOESM12_ESM.zip › Source Data Figure EV3-EV4/ColabFold/NAT2_DNAJA1_PAE.png]

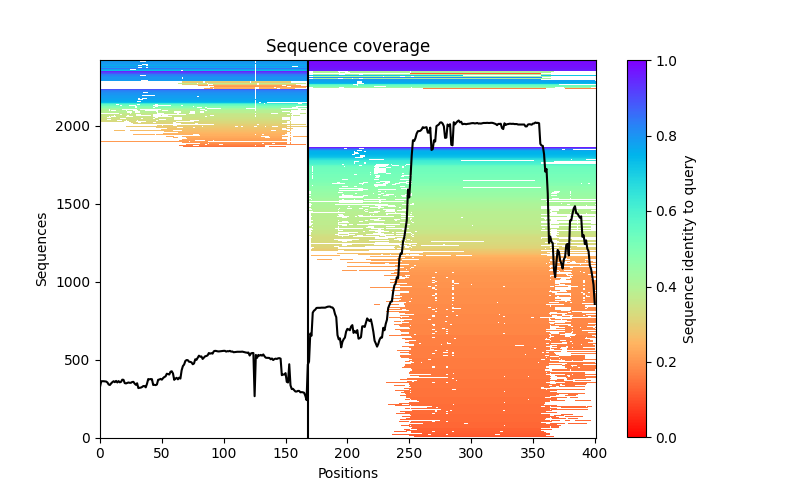

Supplement: Supplementary file 12 — Source Data Fig. 2 [file 44320_2024_19_MOESM12_ESM.zip › Source Data Figure EV3-EV4/ColabFold/BAD_BCL2L1_coverage.png]

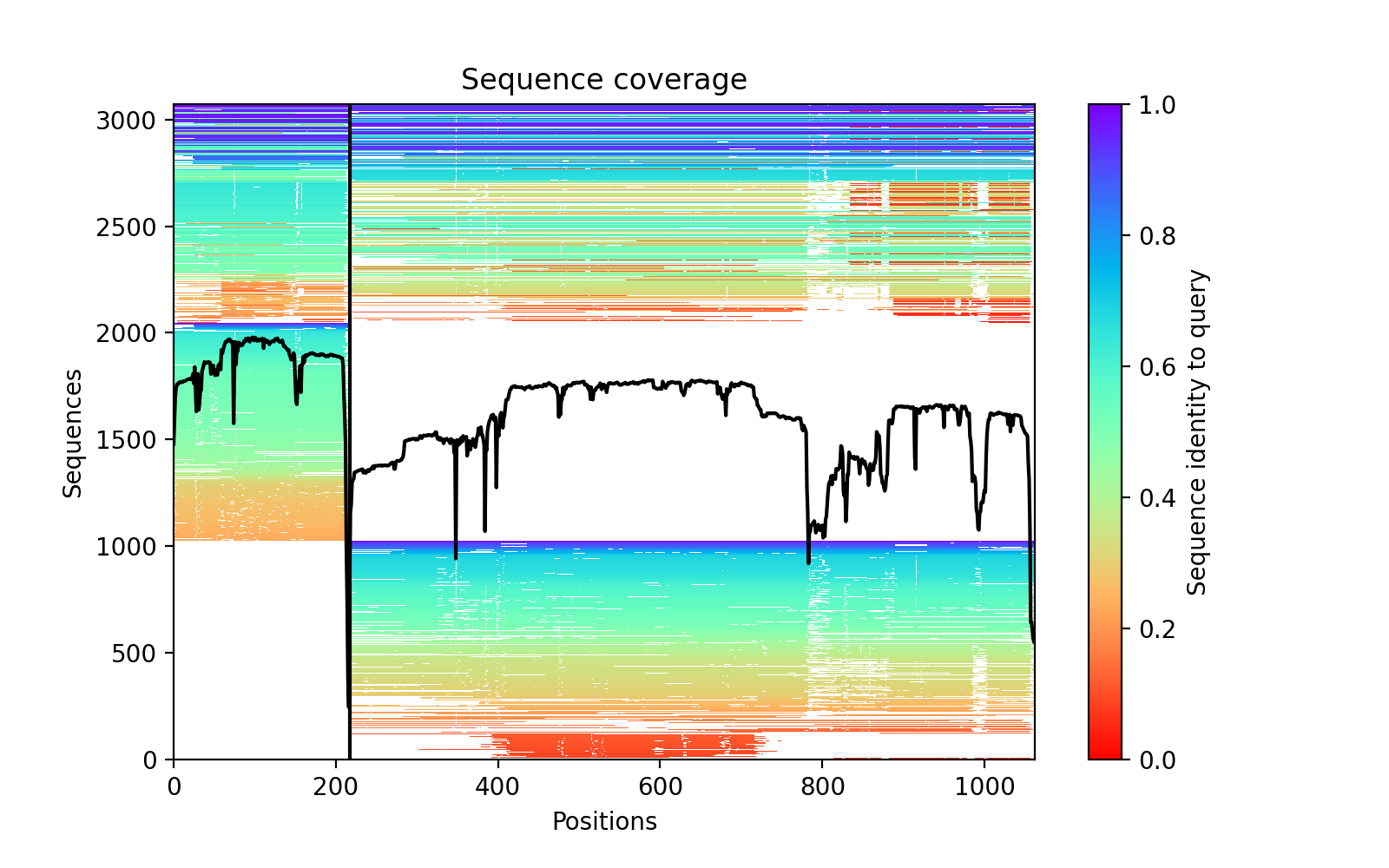

Supplement: Supplementary file 12 — Source Data Fig. 2 [file 44320_2024_19_MOESM12_ESM.zip › Source Data Figure EV3-EV4/ColabFold/GRB2_VAV1_coverage.png]

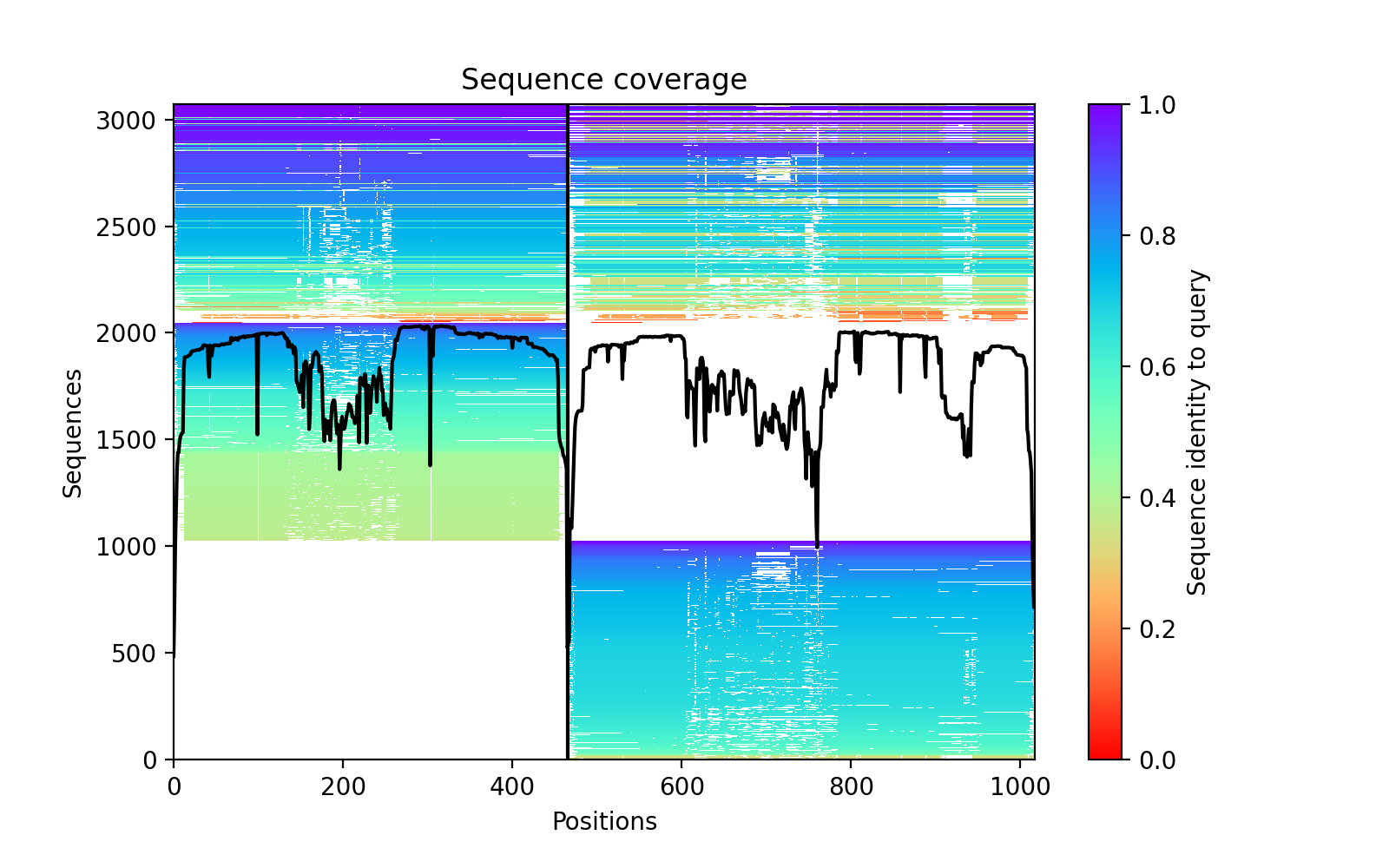

Supplement: Supplementary file 12 — Source Data Fig. 2 [file 44320_2024_19_MOESM12_ESM.zip › Source Data Figure EV3-EV4/ColabFold/SMAD1_SMAD4_coverage.png]

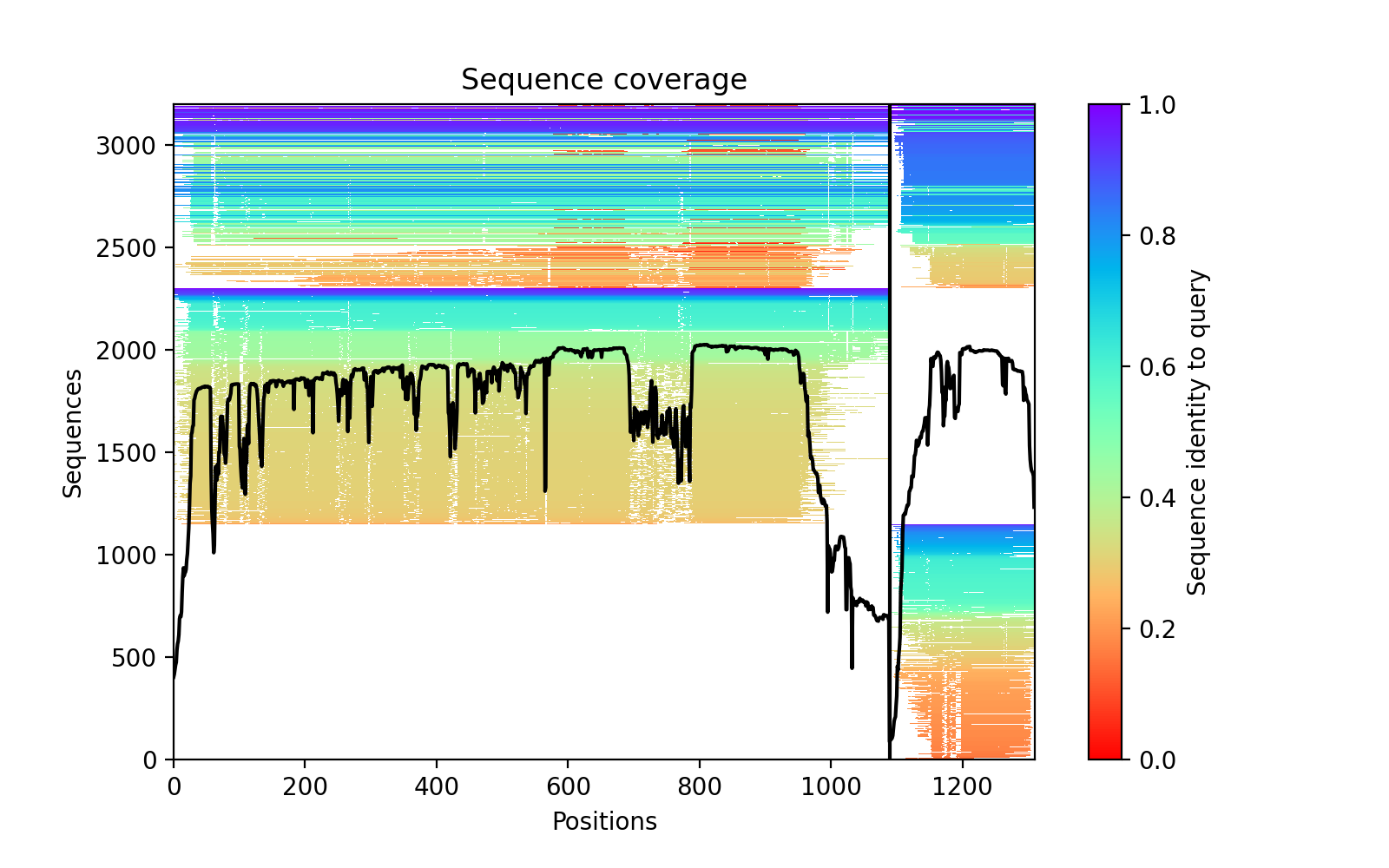

Supplement: Supplementary file 12 — Source Data Fig. 2 [file 44320_2024_19_MOESM12_ESM.zip › Source Data Figure EV3-EV4/ColabFold/PDGFRA_NDFIP1_coverage.png]

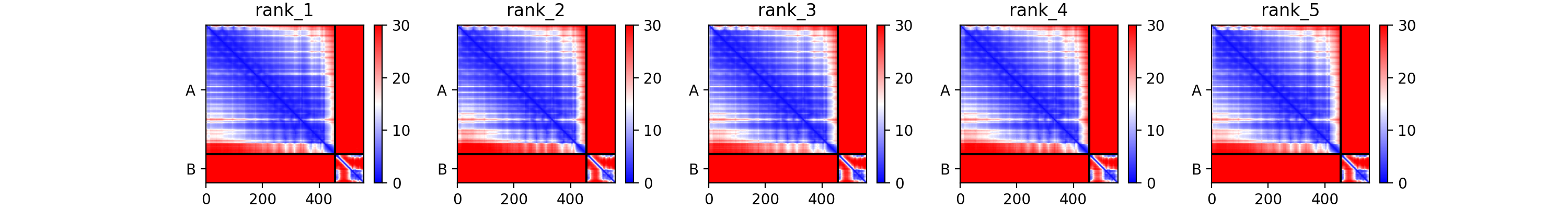

Supplement: Supplementary file 12 — Source Data Fig. 2 [file 44320_2024_19_MOESM12_ESM.zip › Source Data Figure EV3-EV4/ColabFold/PSMD12_CRIPT_PAE.png]

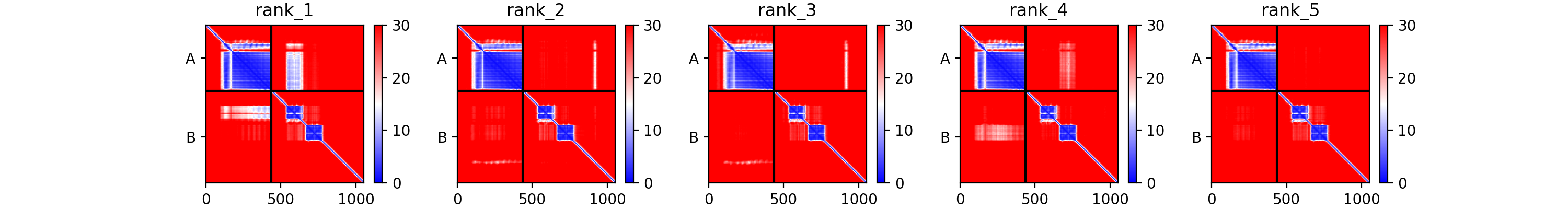

Supplement: Supplementary file 12 — Source Data Fig. 2 [file 44320_2024_19_MOESM12_ESM.zip › Source Data Figure EV3-EV4/ColabFold/BYSL_KIAA0907_PAE.png]

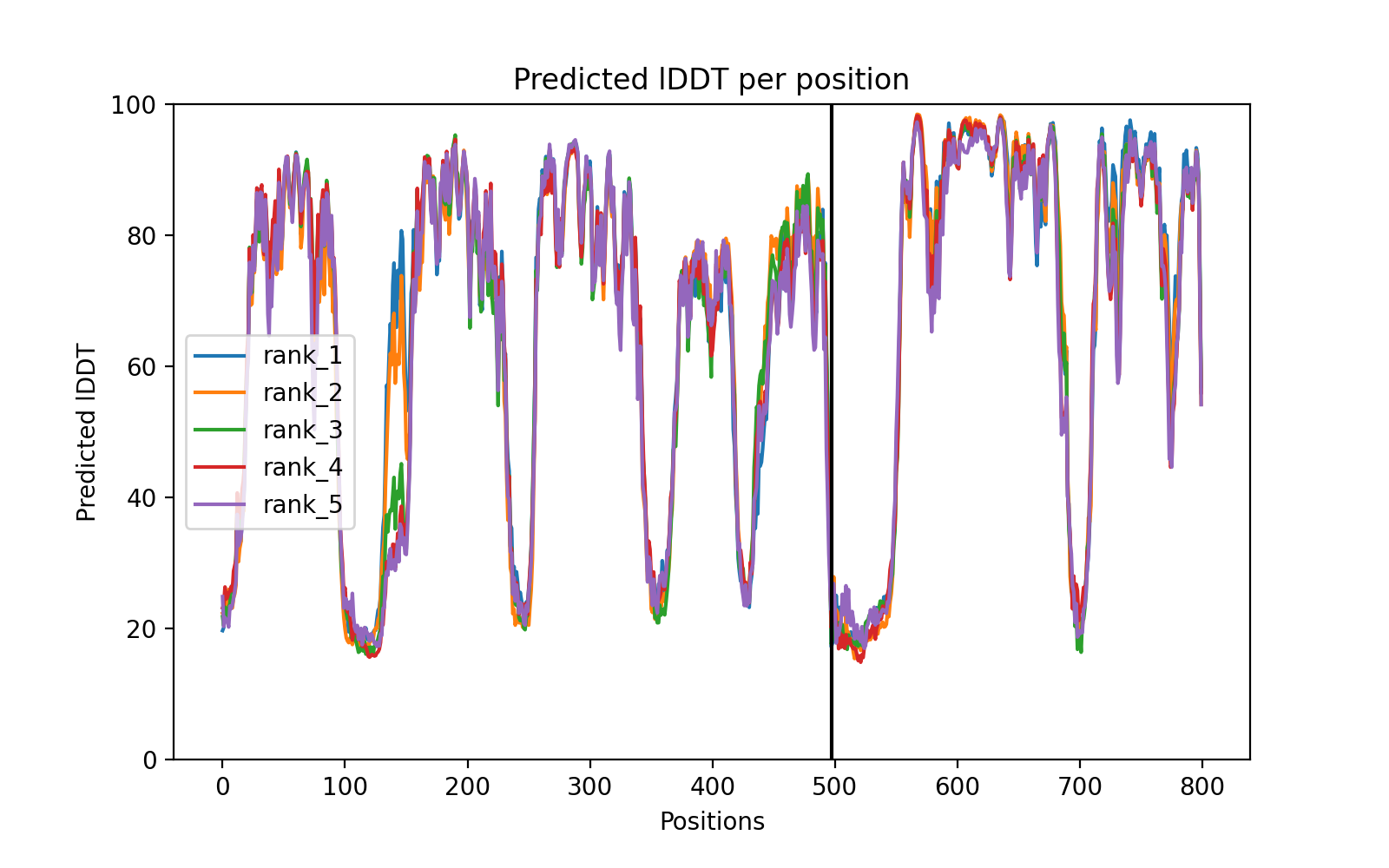

Supplement: Supplementary file 12 — Source Data Fig. 2 [file 44320_2024_19_MOESM12_ESM.zip › Source Data Figure EV3-EV4/ColabFold/XIAP_CASP7_plddt.png]

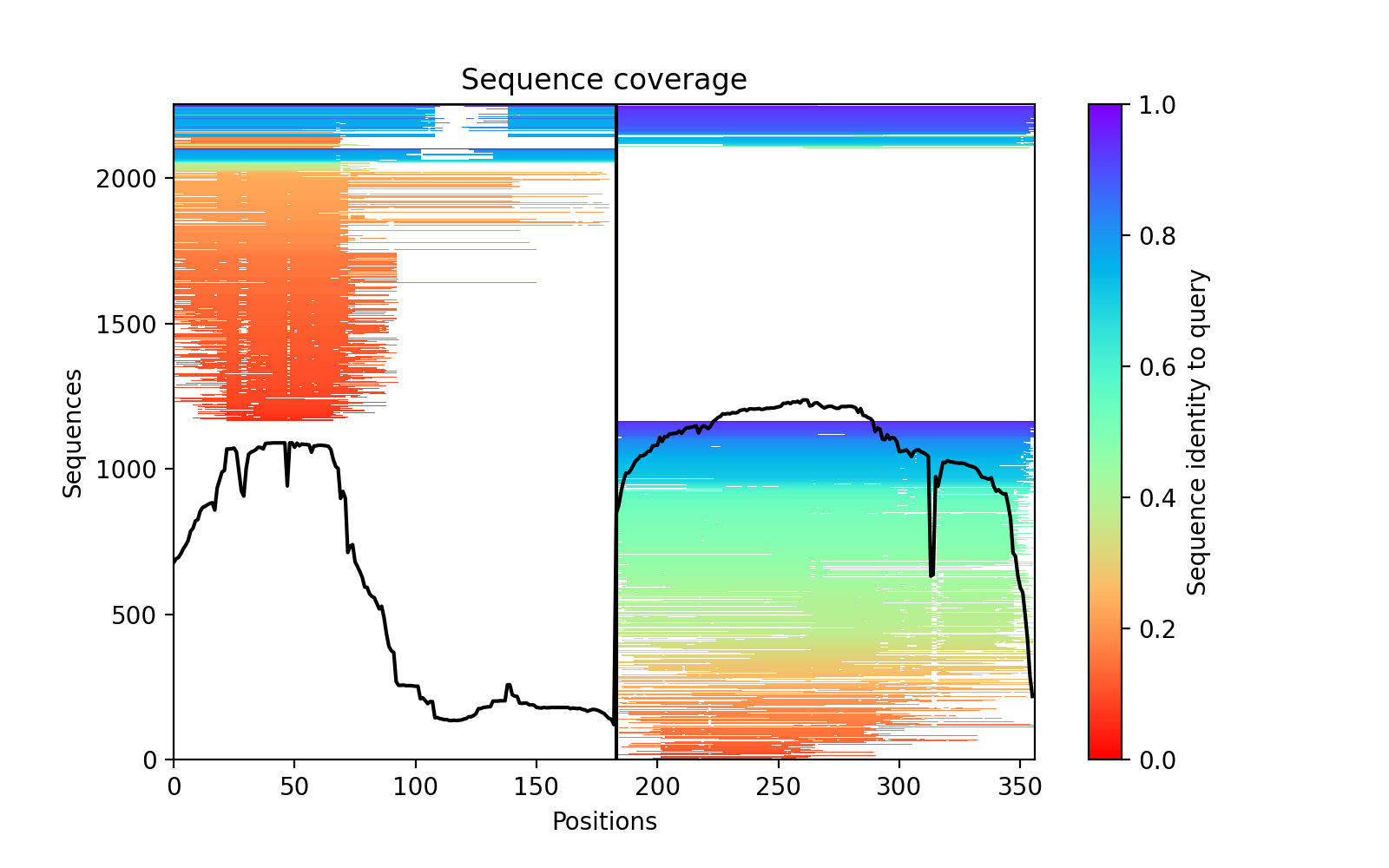

Supplement: Supplementary file 12 — Source Data Fig. 2 [file 44320_2024_19_MOESM12_ESM.zip › Source Data Figure EV3-EV4/ColabFold/MOBP_MRPS25_coverage.png]

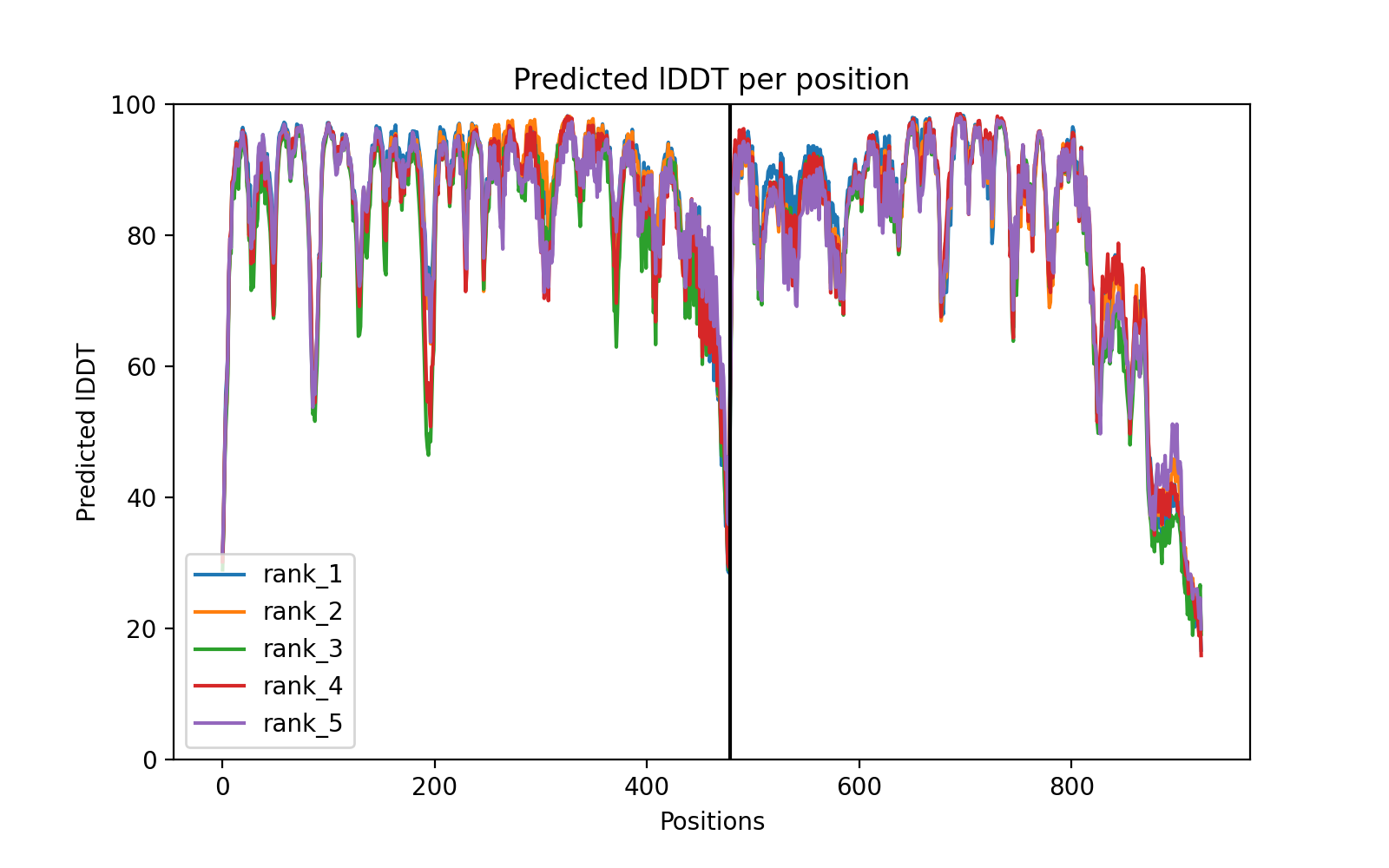

Supplement: Supplementary file 12 — Source Data Fig. 2 [file 44320_2024_19_MOESM12_ESM.zip › Source Data Figure EV3-EV4/ColabFold/IFIT1_EIF3E_plddt.png]

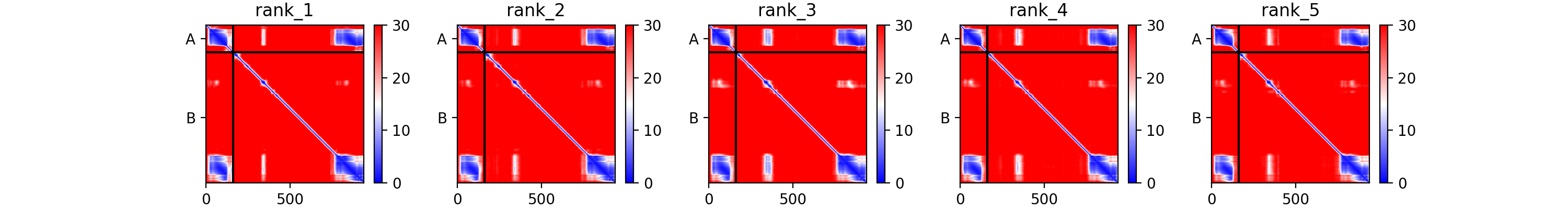

Supplement: Supplementary file 12 — Source Data Fig. 2 [file 44320_2024_19_MOESM12_ESM.zip › Source Data Figure EV3-EV4/ColabFold/MAFG_NFE2L1_PAE.png]

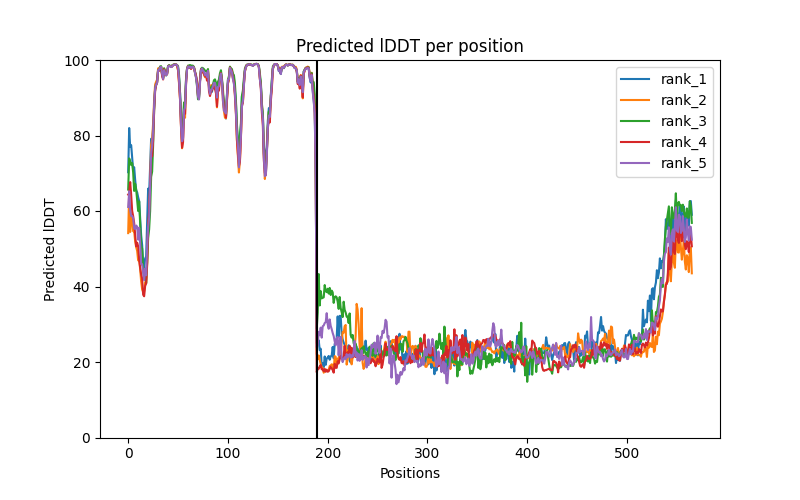

Supplement: Supplementary file 12 — Source Data Fig. 2 [file 44320_2024_19_MOESM12_ESM.zip › Source Data Figure EV3-EV4/ColabFold/APOD_MUC7_plddt.png]

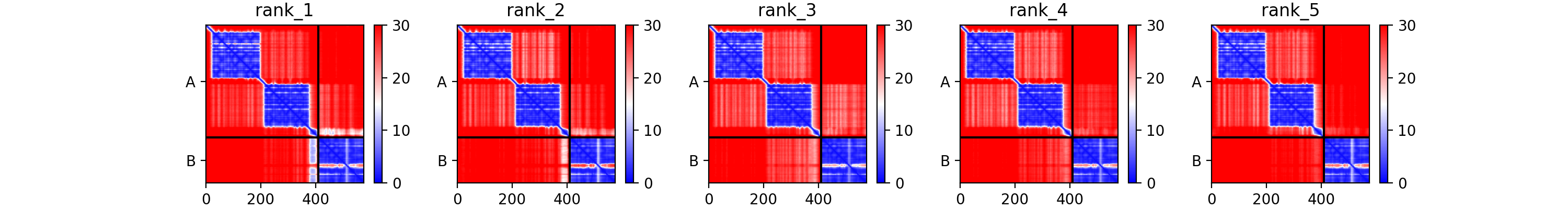

Supplement: Supplementary file 12 — Source Data Fig. 2 [file 44320_2024_19_MOESM12_ESM.zip › Source Data Figure EV3-EV4/ColabFold/LAMP2_UBE2G2_PAE.png]

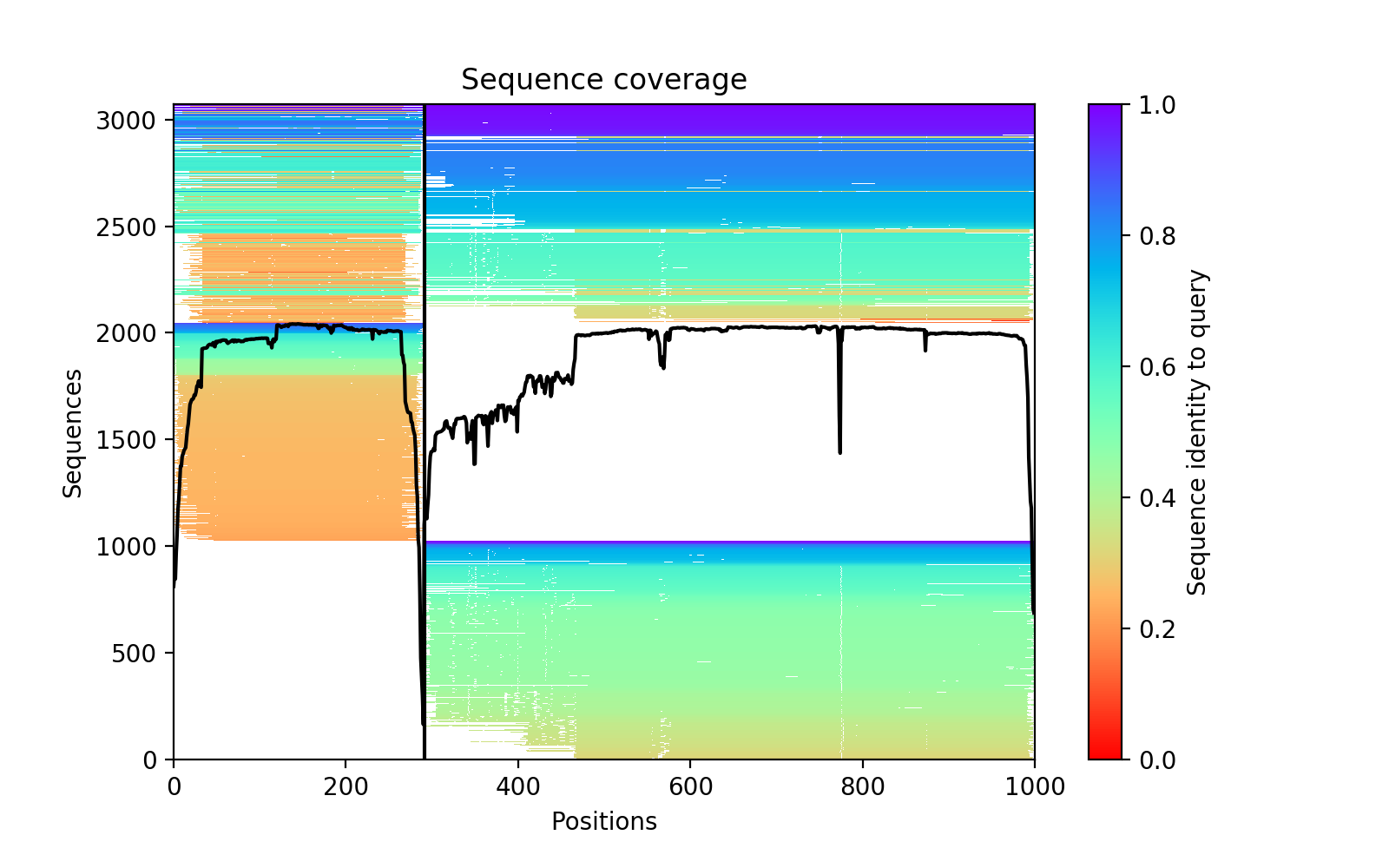

Supplement: Supplementary file 12 — Source Data Fig. 2 [file 44320_2024_19_MOESM12_ESM.zip › Source Data Figure EV3-EV4/ColabFold/RGR_ABCF3_coverage.png]

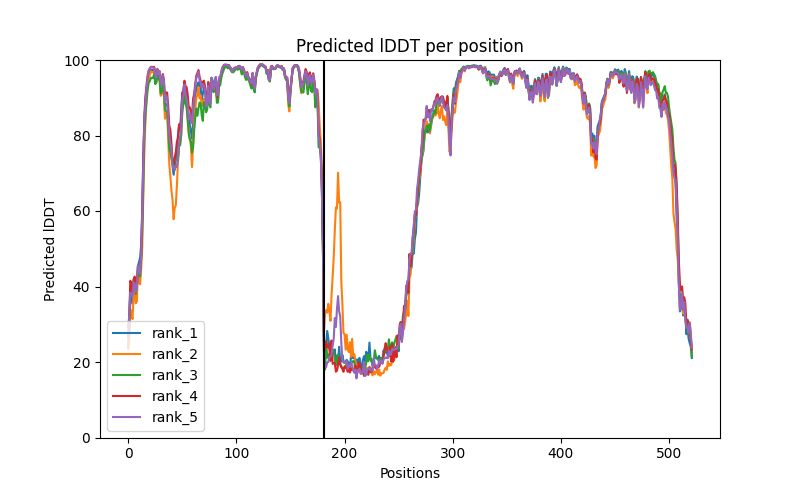

Supplement: Supplementary file 12 — Source Data Fig. 2 [file 44320_2024_19_MOESM12_ESM.zip › Source Data Figure EV3-EV4/ColabFold/ARF1_ARFIP2_plddt.png]

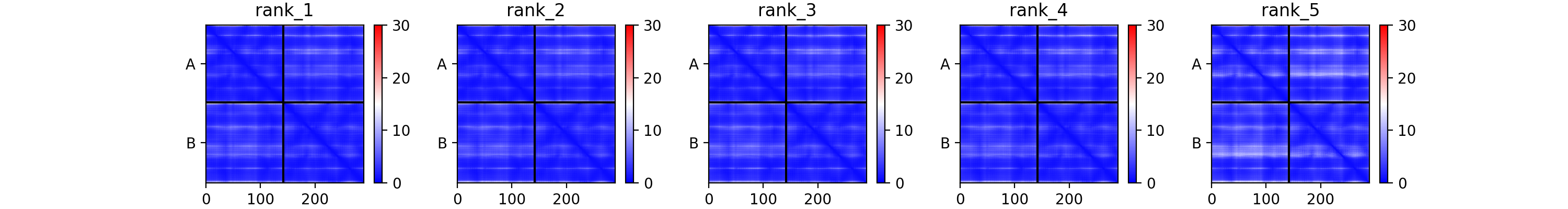

Supplement: Supplementary file 12 — Source Data Fig. 2 [file 44320_2024_19_MOESM12_ESM.zip › Source Data Figure EV3-EV4/ColabFold/HBA2_HBB_PAE.png]

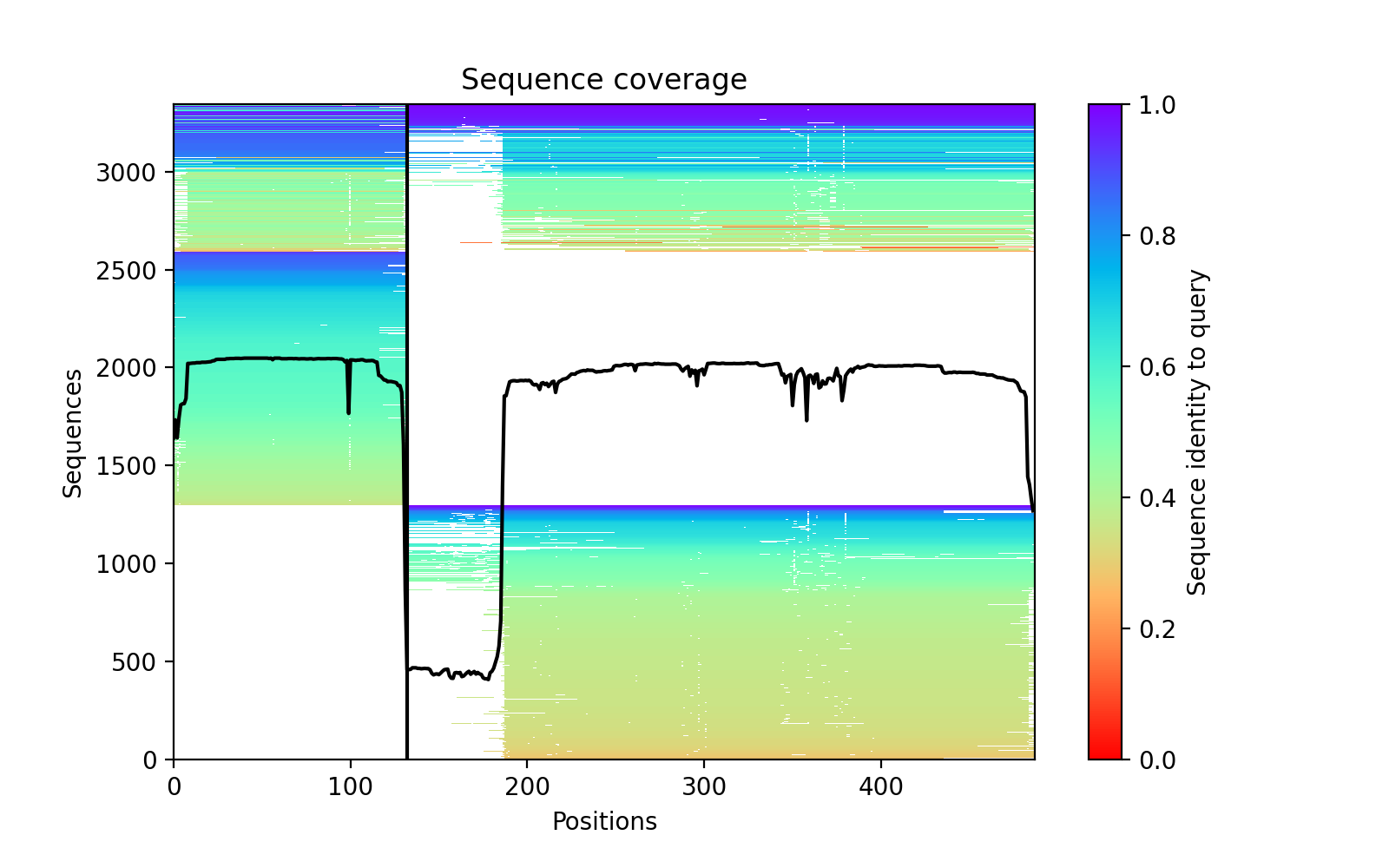

Supplement: Supplementary file 12 — Source Data Fig. 2 [file 44320_2024_19_MOESM12_ESM.zip › Source Data Figure EV3-EV4/ColabFold/FABP7_STX5_coverage.png]

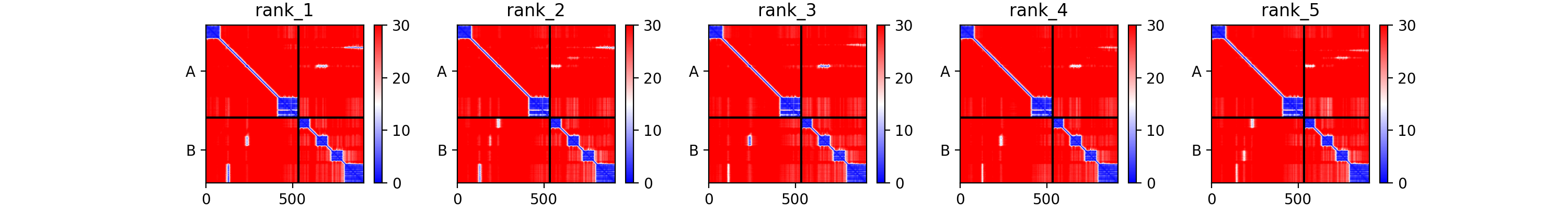

Supplement: Supplementary file 12 — Source Data Fig. 2 [file 44320_2024_19_MOESM12_ESM.zip › Source Data Figure EV3-EV4/ColabFold/LCP2_NCK1_PAE.png]

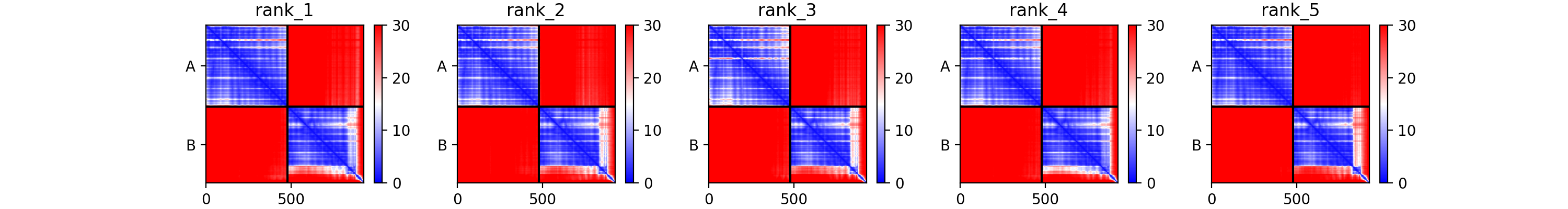

Supplement: Supplementary file 12 — Source Data Fig. 2 [file 44320_2024_19_MOESM12_ESM.zip › Source Data Figure EV3-EV4/ColabFold/IFIT1_EIF3E_PAE.png]

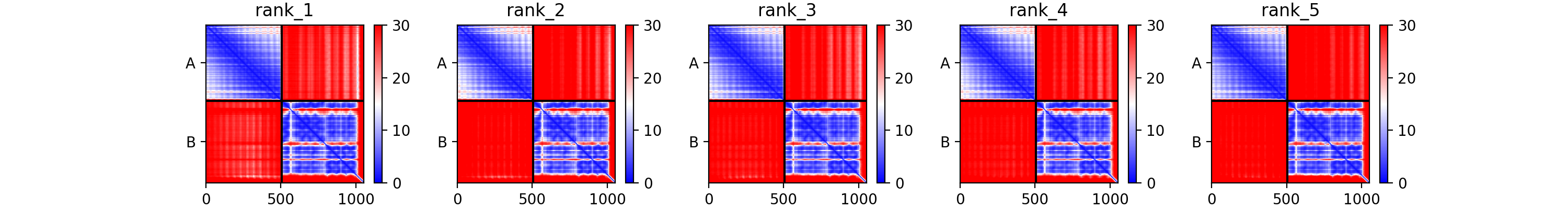

Supplement: Supplementary file 12 — Source Data Fig. 2 [file 44320_2024_19_MOESM12_ESM.zip › Source Data Figure EV3-EV4/ColabFold/PSMD5_SLC22A15_PAE.png]

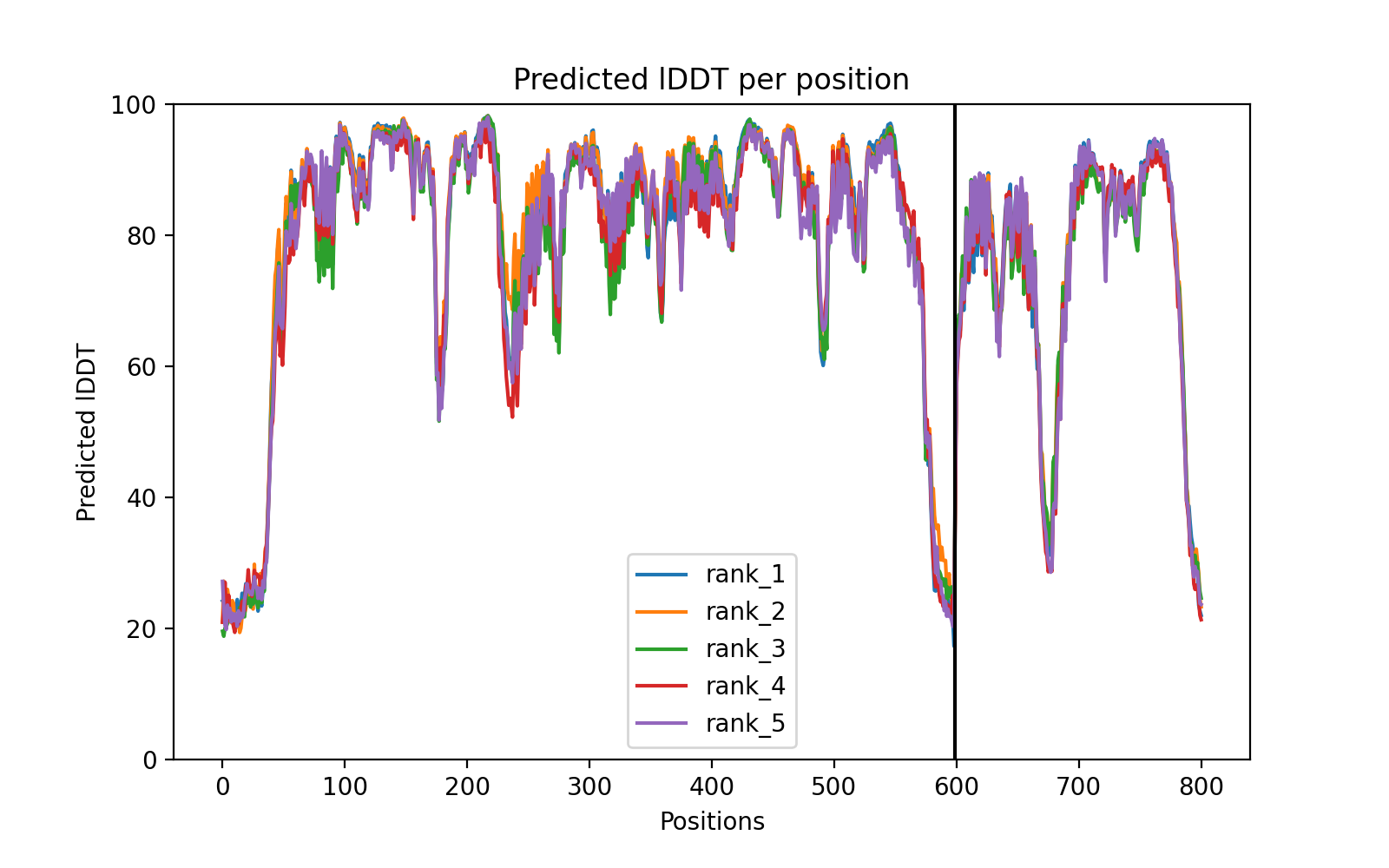

Supplement: Supplementary file 12 — Source Data Fig. 2 [file 44320_2024_19_MOESM12_ESM.zip › Source Data Figure EV3-EV4/ColabFold/SLC6A1_TM4SF4_plddt.png]

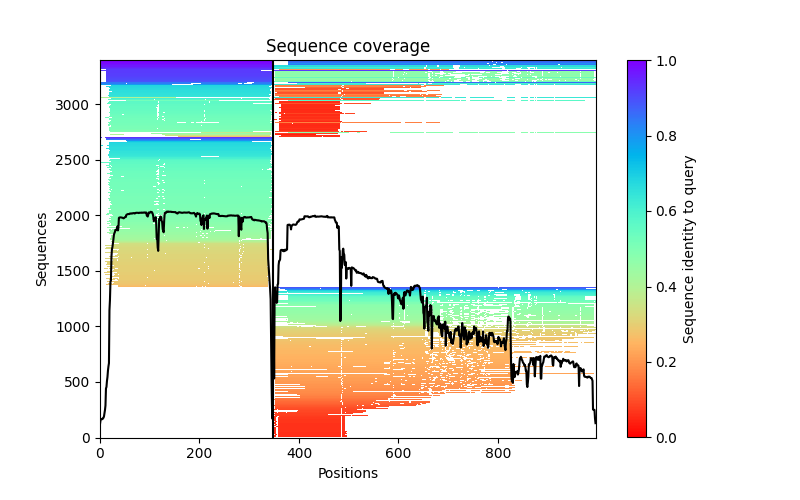

Supplement: Supplementary file 12 — Source Data Fig. 2 [file 44320_2024_19_MOESM12_ESM.zip › Source Data Figure EV3-EV4/ColabFold/ARSA_DBN1_coverage.png]

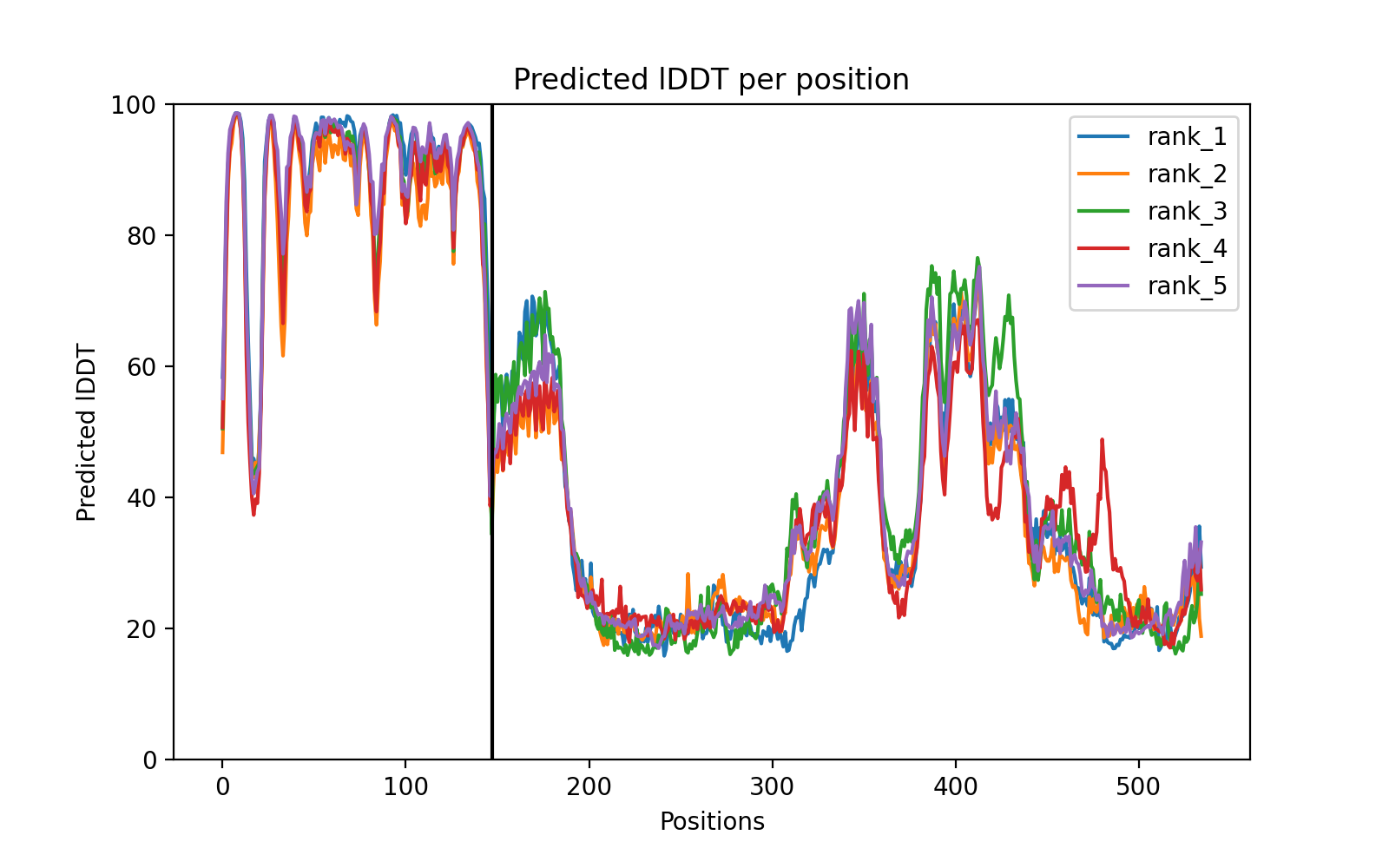

Supplement: Supplementary file 12 — Source Data Fig. 2 [file 44320_2024_19_MOESM12_ESM.zip › Source Data Figure EV3-EV4/ColabFold/NUDT2_MIIP_plddt.png]

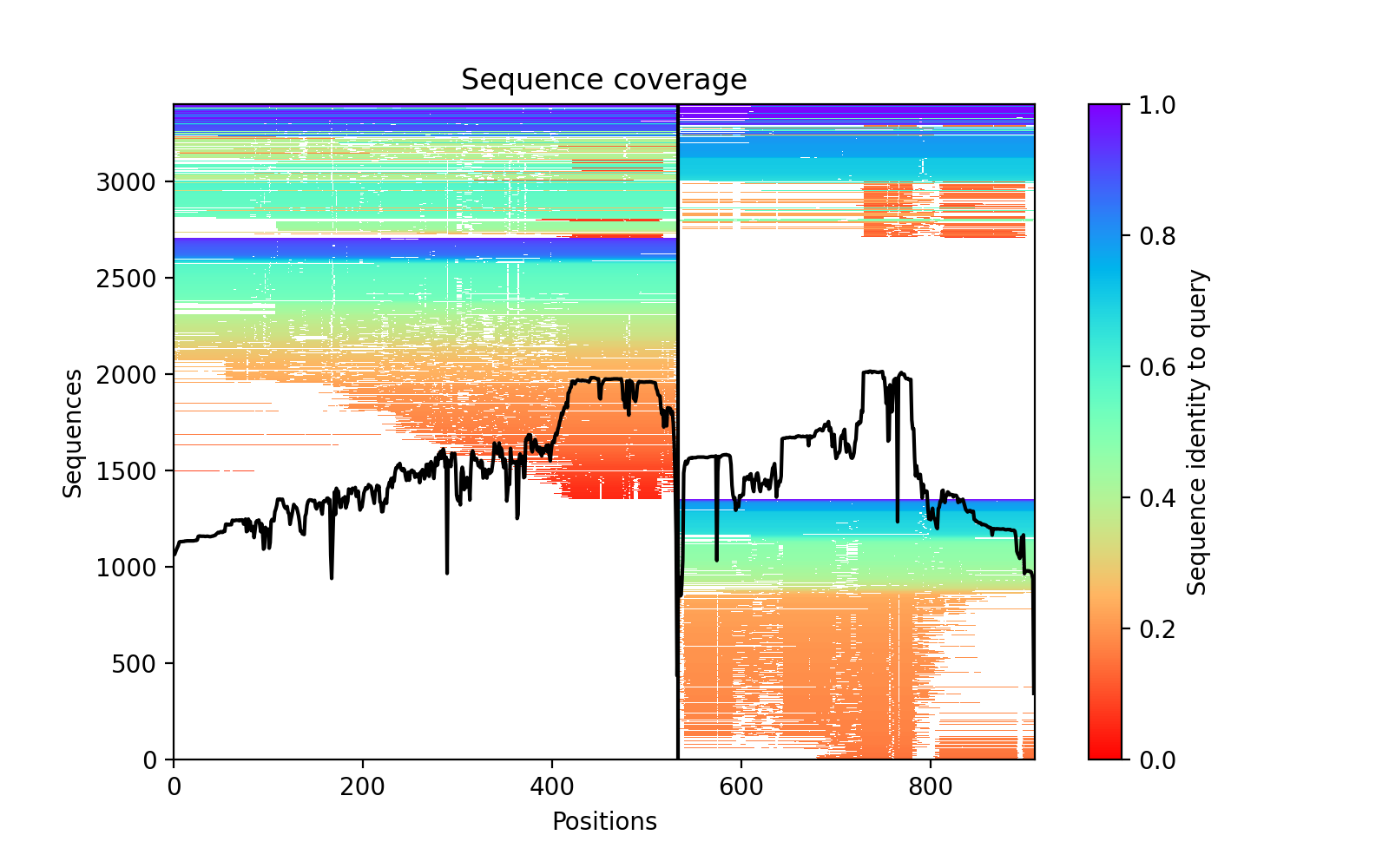

Supplement: Supplementary file 12 — Source Data Fig. 2 [file 44320_2024_19_MOESM12_ESM.zip › Source Data Figure EV3-EV4/ColabFold/LCP2_NCK1_coverage.png]

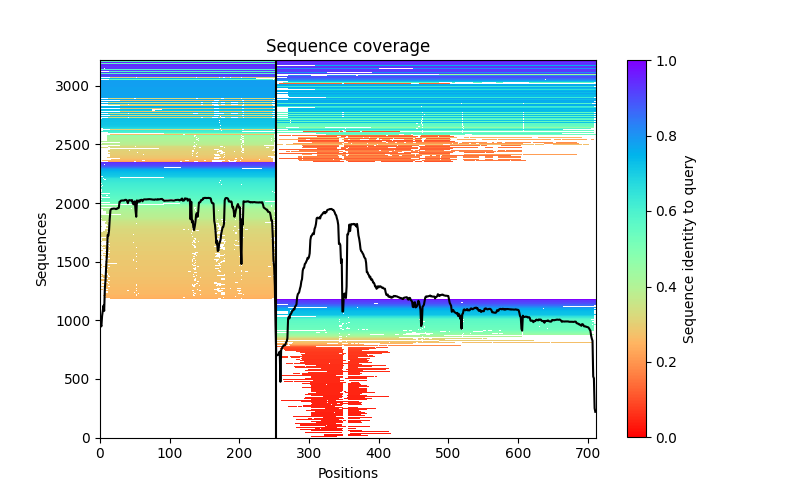

Supplement: Supplementary file 12 — Source Data Fig. 2 [file 44320_2024_19_MOESM12_ESM.zip › Source Data Figure EV3-EV4/ColabFold/CD151_WDR41_coverage.png]

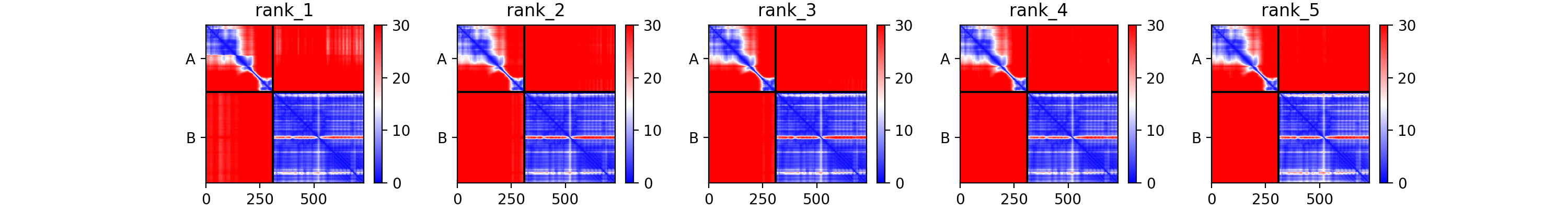

Supplement: Supplementary file 12 — Source Data Fig. 2 [file 44320_2024_19_MOESM12_ESM.zip › Source Data Figure EV3-EV4/ColabFold/MNAT1_GMPPA_PAE.png]

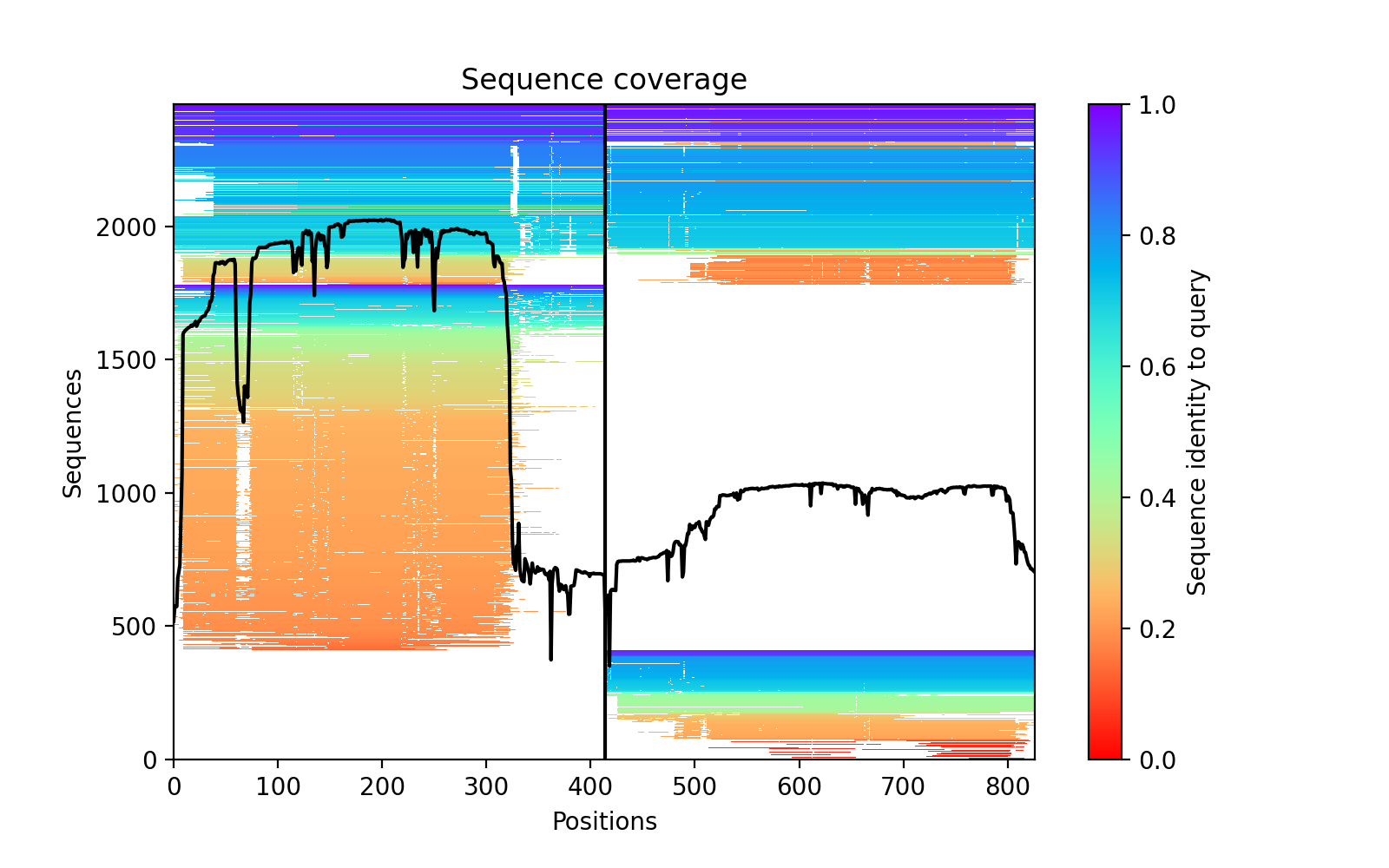

Supplement: Supplementary file 12 — Source Data Fig. 2 [file 44320_2024_19_MOESM12_ESM.zip › Source Data Figure EV3-EV4/ColabFold/ITPK1_TMEM22_coverage.png]

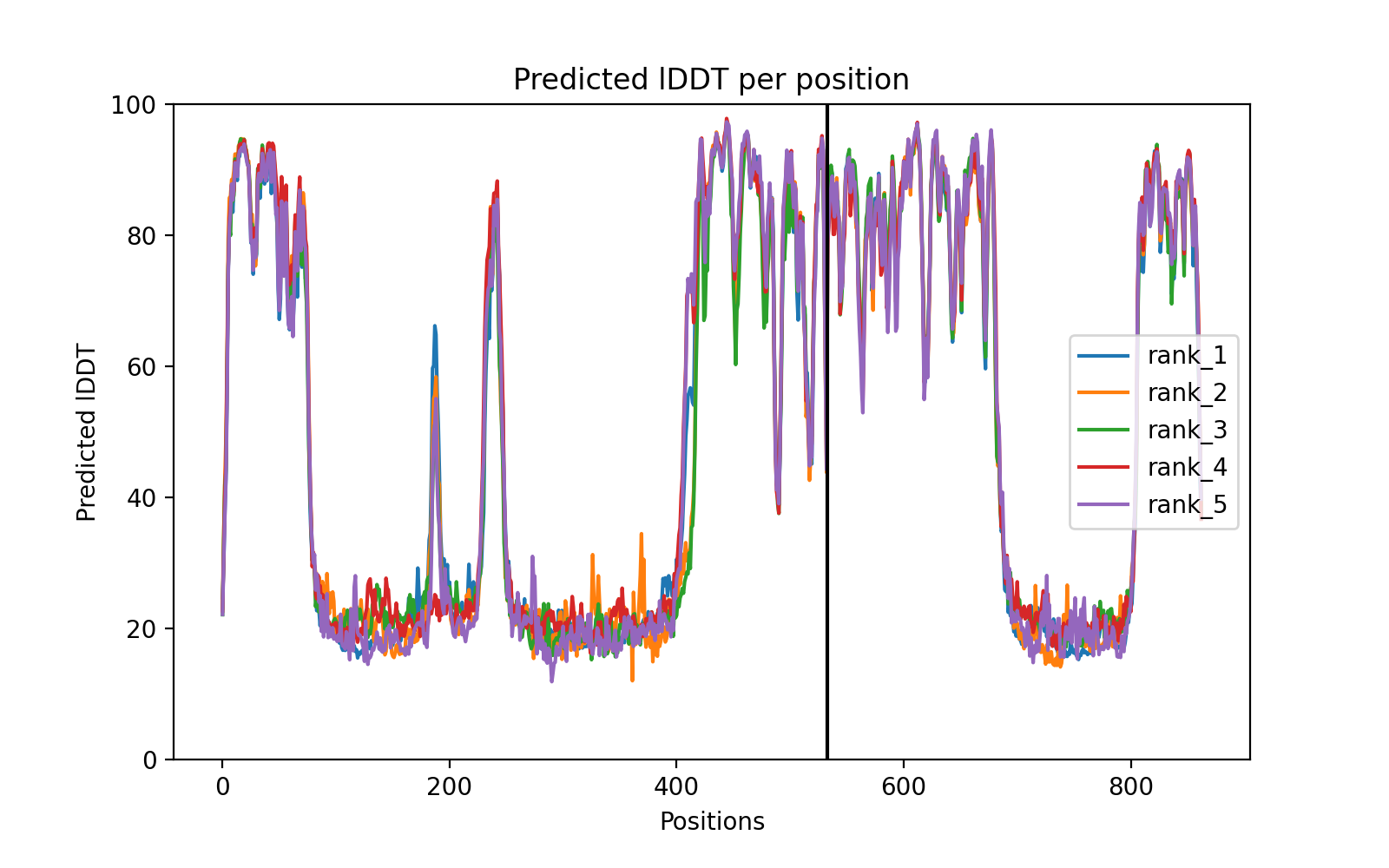

Supplement: Supplementary file 12 — Source Data Fig. 2 [file 44320_2024_19_MOESM12_ESM.zip › Source Data Figure EV3-EV4/ColabFold/LCP2_GRAP2_plddt.png]

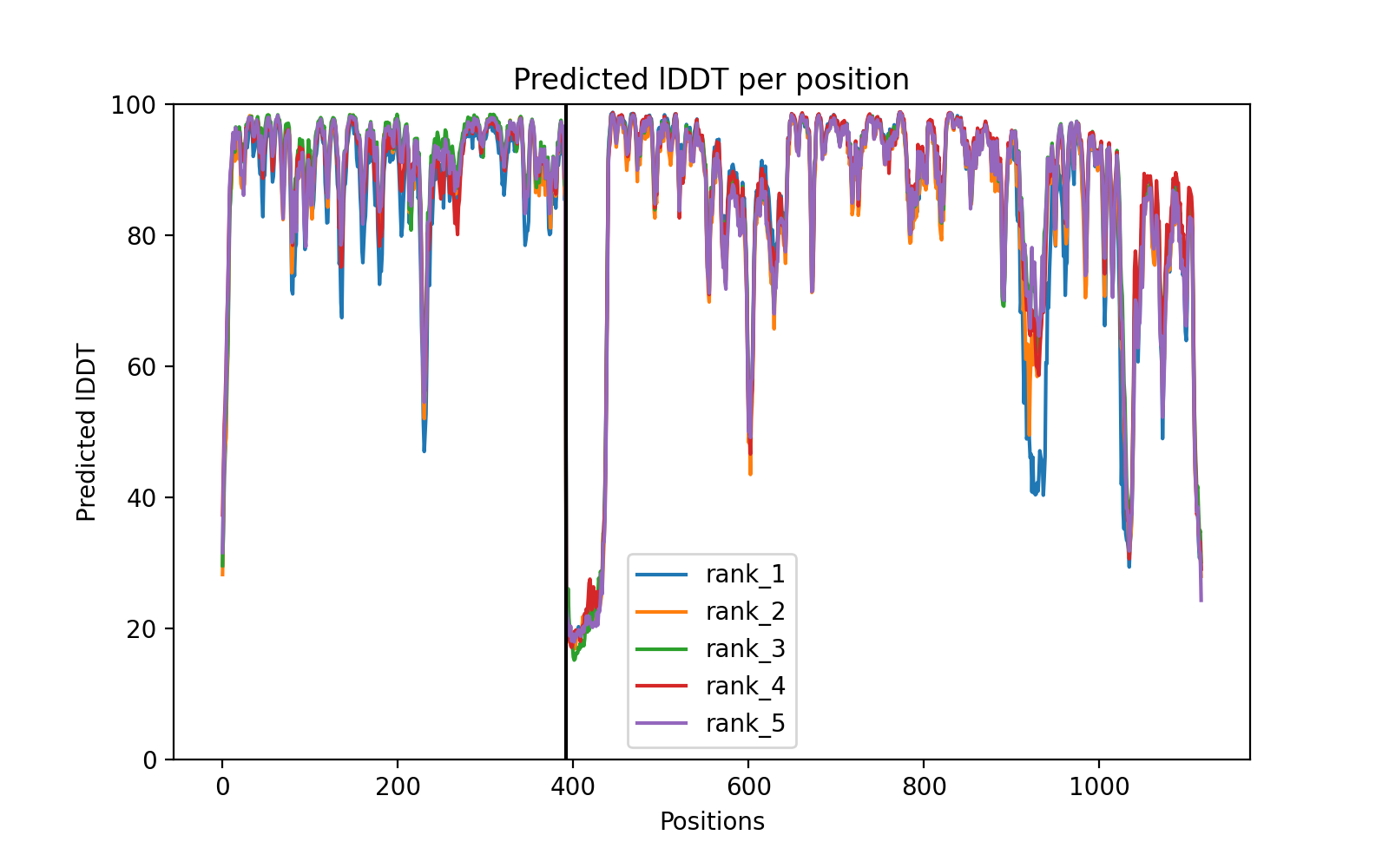

Supplement: Supplementary file 12 — Source Data Fig. 2 [file 44320_2024_19_MOESM12_ESM.zip › Source Data Figure EV3-EV4/ColabFold/GALK1_MCCC1_plddt.png]

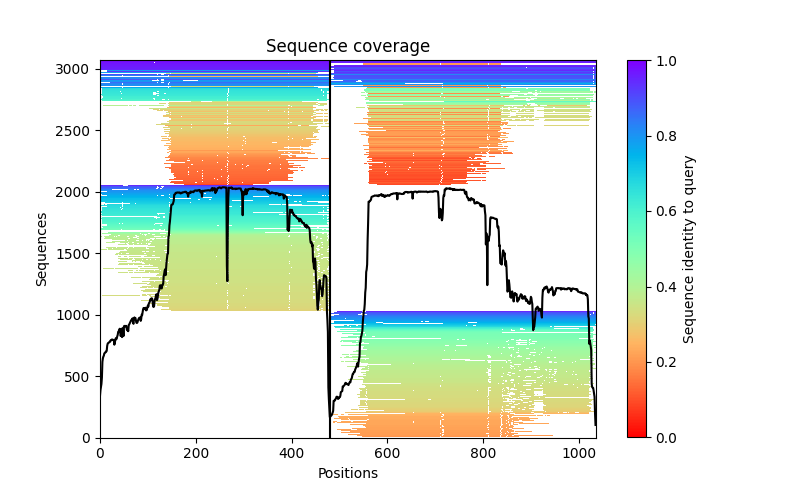

Supplement: Supplementary file 12 — Source Data Fig. 2 [file 44320_2024_19_MOESM12_ESM.zip › Source Data Figure EV3-EV4/ColabFold/AKT1_PDPK1_coverage.png]

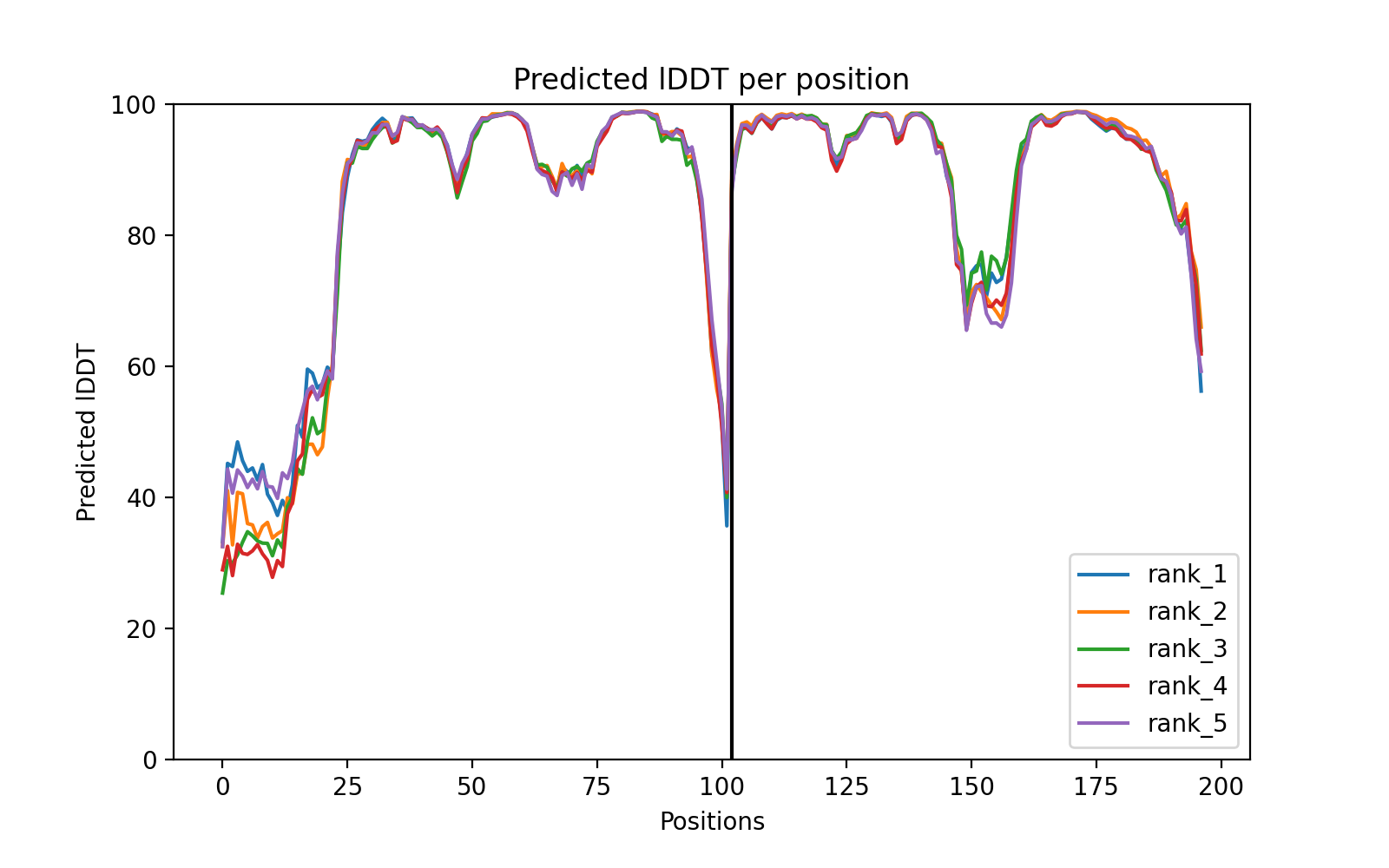

Supplement: Supplementary file 12 — Source Data Fig. 2 [file 44320_2024_19_MOESM12_ESM.zip › Source Data Figure EV3-EV4/ColabFold/LSM3_LSM2_plddt.png]

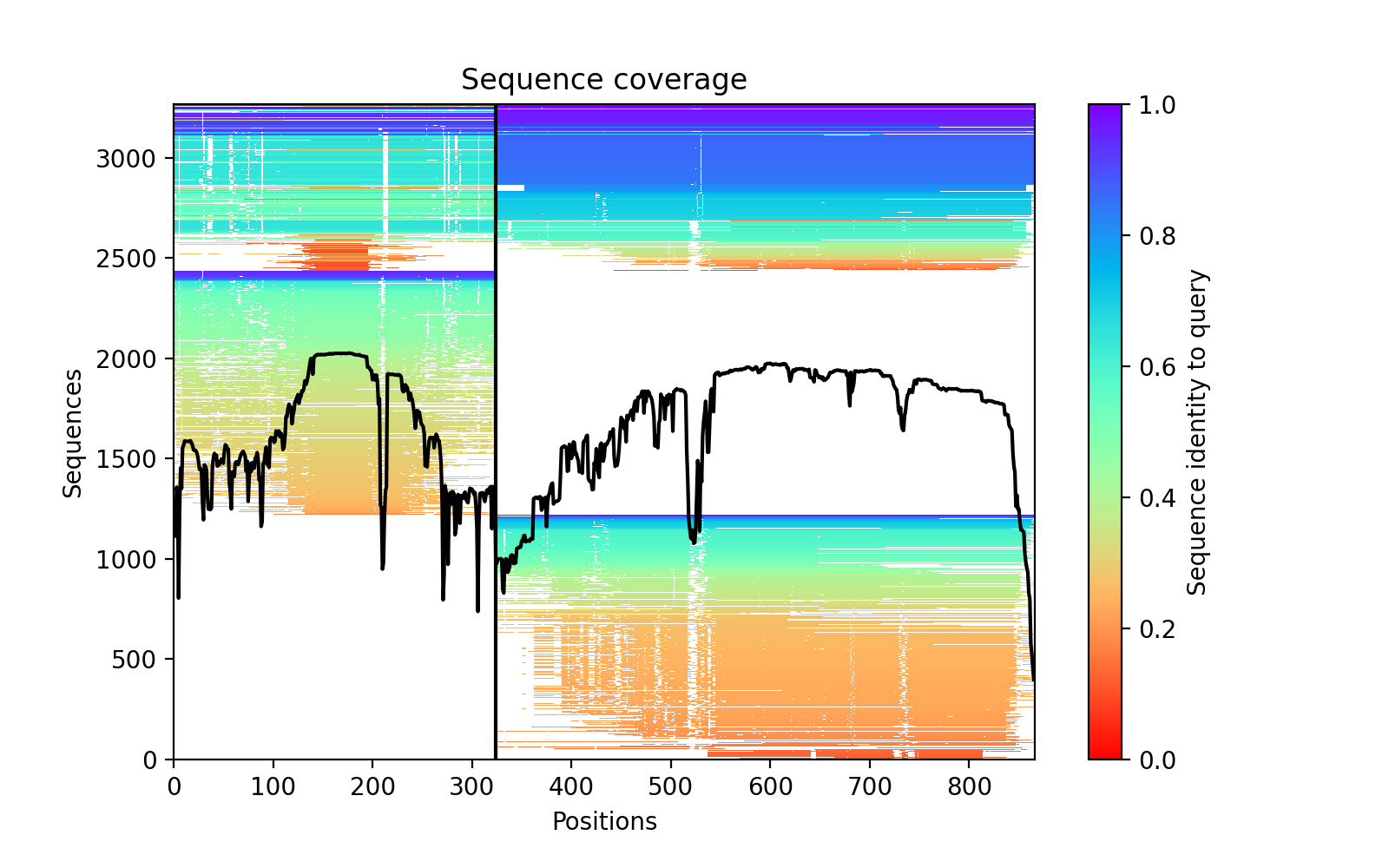

Supplement: Supplementary file 12 — Source Data Fig. 2 [file 44320_2024_19_MOESM12_ESM.zip › Source Data Figure EV3-EV4/ColabFold/NKX2-5_CSGALNACT2_coverage.png]

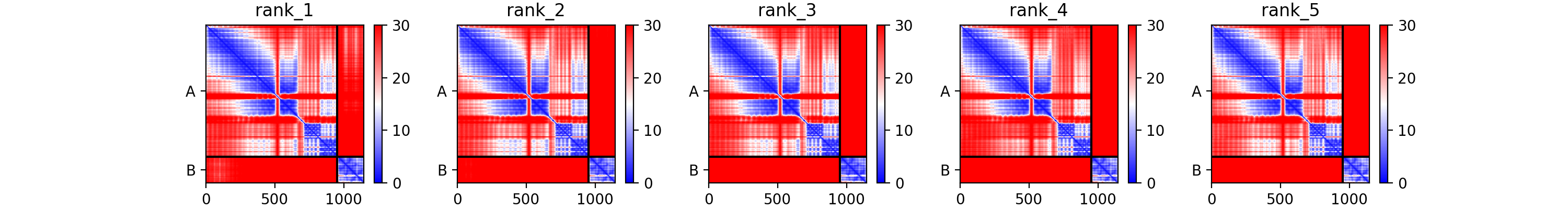

Supplement: Supplementary file 12 — Source Data Fig. 2 [file 44320_2024_19_MOESM12_ESM.zip › Source Data Figure EV3-EV4/ColabFold/COPB1_HPCAL4_PAE.png]

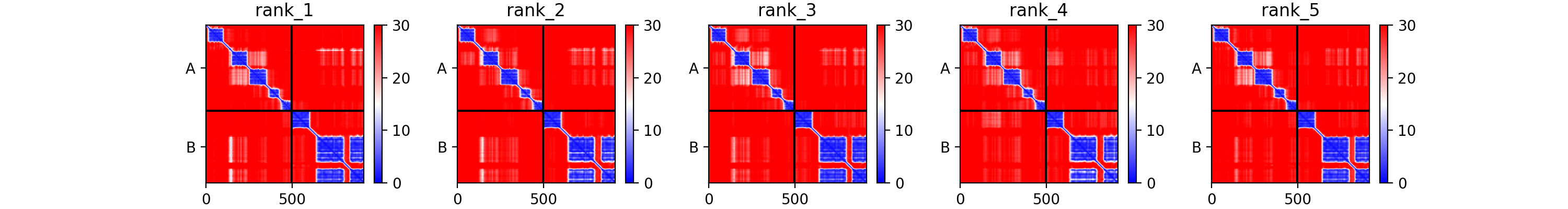

Supplement: Supplementary file 12 — Source Data Fig. 2 [file 44320_2024_19_MOESM12_ESM.zip › Source Data Figure EV3-EV4/ColabFold/XIAP_CASP9_PAE.png]

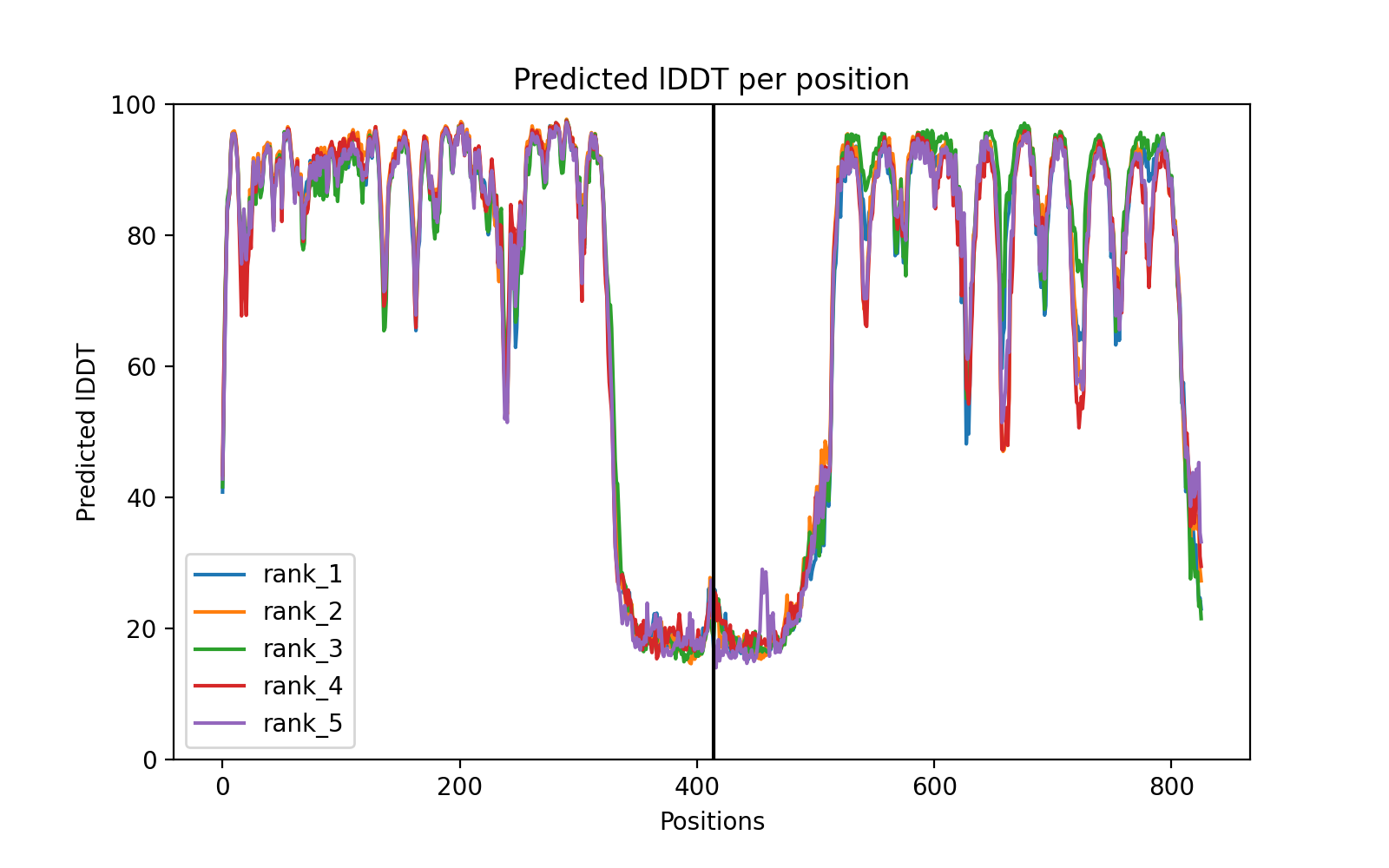

Supplement: Supplementary file 12 — Source Data Fig. 2 [file 44320_2024_19_MOESM12_ESM.zip › Source Data Figure EV3-EV4/ColabFold/ITPK1_TMEM22_plddt.png]

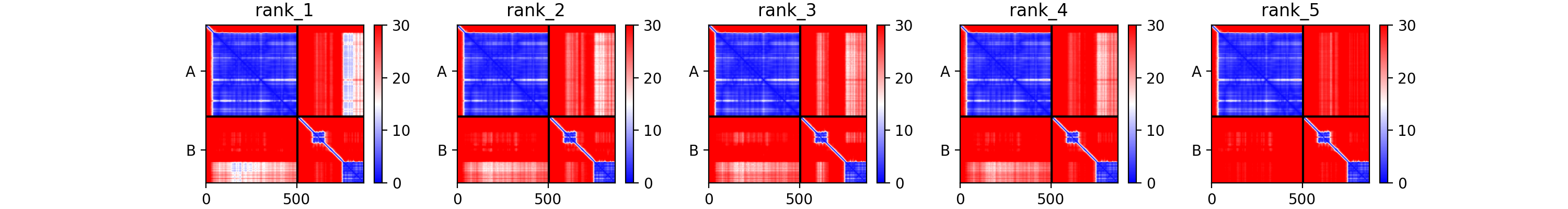

Supplement: Supplementary file 12 — Source Data Fig. 2 [file 44320_2024_19_MOESM12_ESM.zip › Source Data Figure EV3-EV4/ColabFold/SHMT2_STAC3_PAE.png]

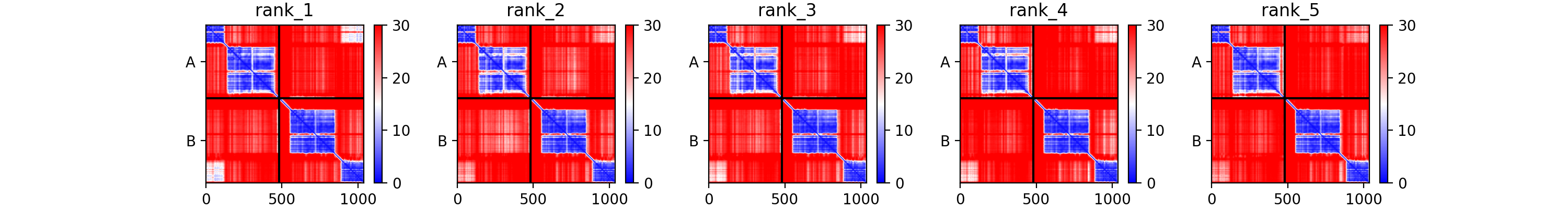

Supplement: Supplementary file 12 — Source Data Fig. 2 [file 44320_2024_19_MOESM12_ESM.zip › Source Data Figure EV3-EV4/ColabFold/AKT1_PDPK1_PAE.png]

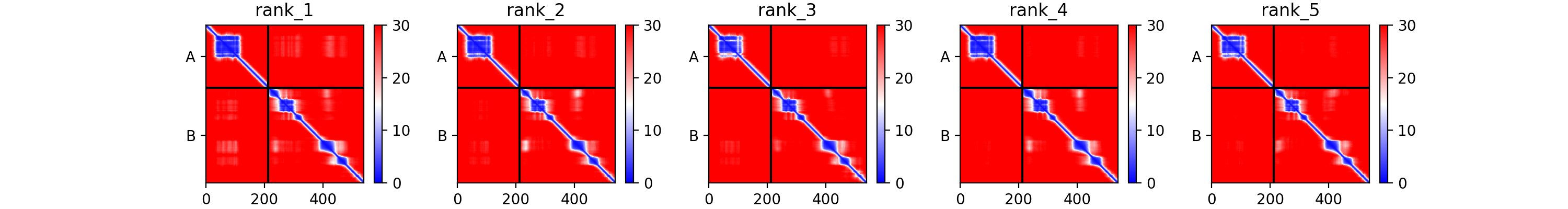

Supplement: Supplementary file 12 — Source Data Fig. 2 [file 44320_2024_19_MOESM12_ESM.zip › Source Data Figure EV3-EV4/ColabFold/HIST1H1C_NPDC1_PAE.png]

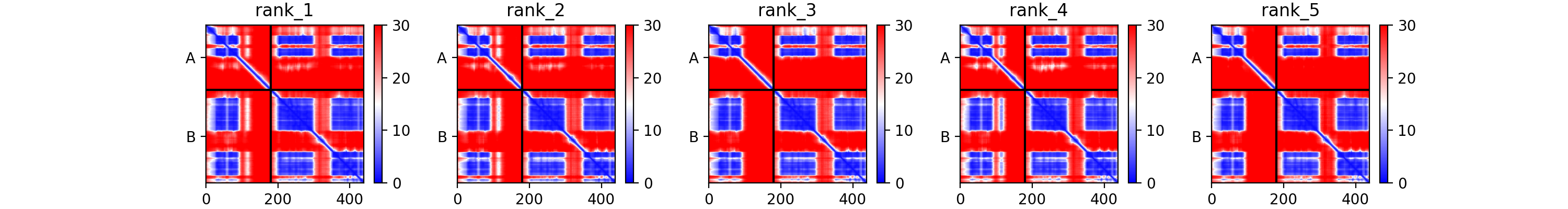

Supplement: Supplementary file 12 — Source Data Fig. 2 [file 44320_2024_19_MOESM12_ESM.zip › Source Data Figure EV3-EV4/ColabFold/IGF2_IGFBP4_PAE.png]

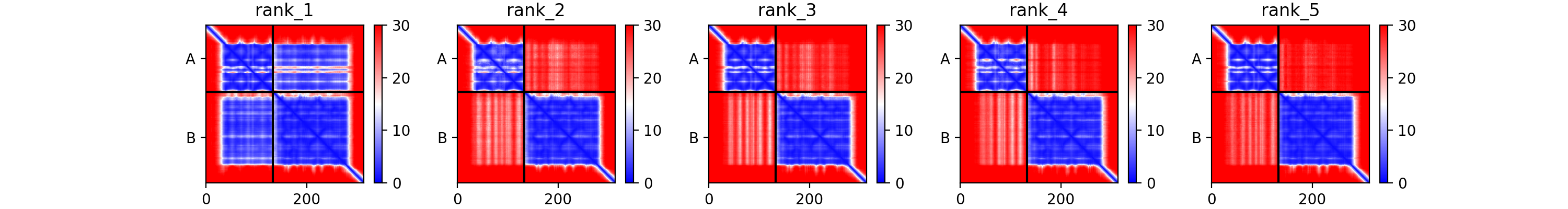

Supplement: Supplementary file 12 — Source Data Fig. 2 [file 44320_2024_19_MOESM12_ESM.zip › Source Data Figure EV3-EV4/ColabFold/NDP_NUDT4_PAE.png]

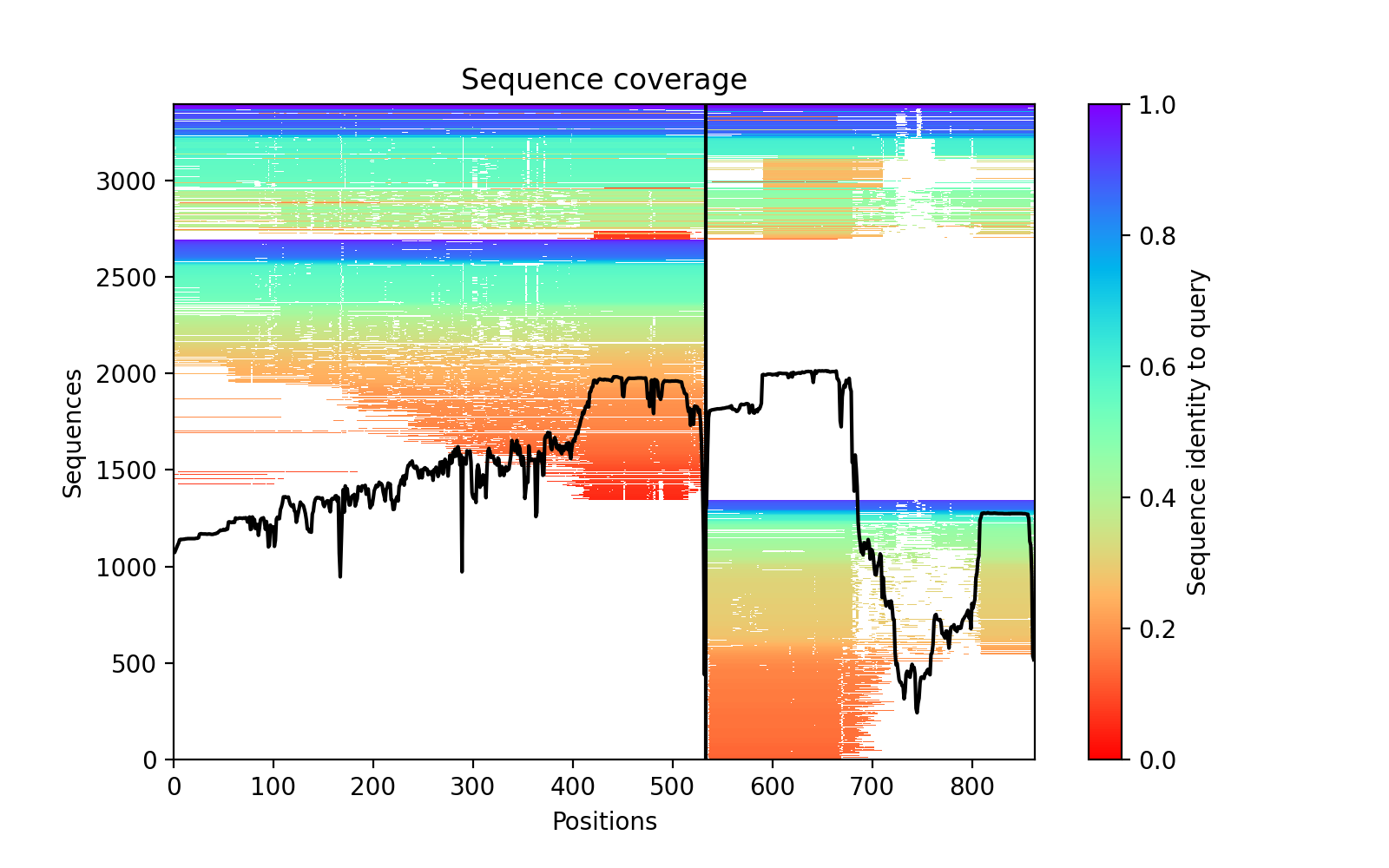

Supplement: Supplementary file 12 — Source Data Fig. 2 [file 44320_2024_19_MOESM12_ESM.zip › Source Data Figure EV3-EV4/ColabFold/LCP2_GRAP2_coverage.png]

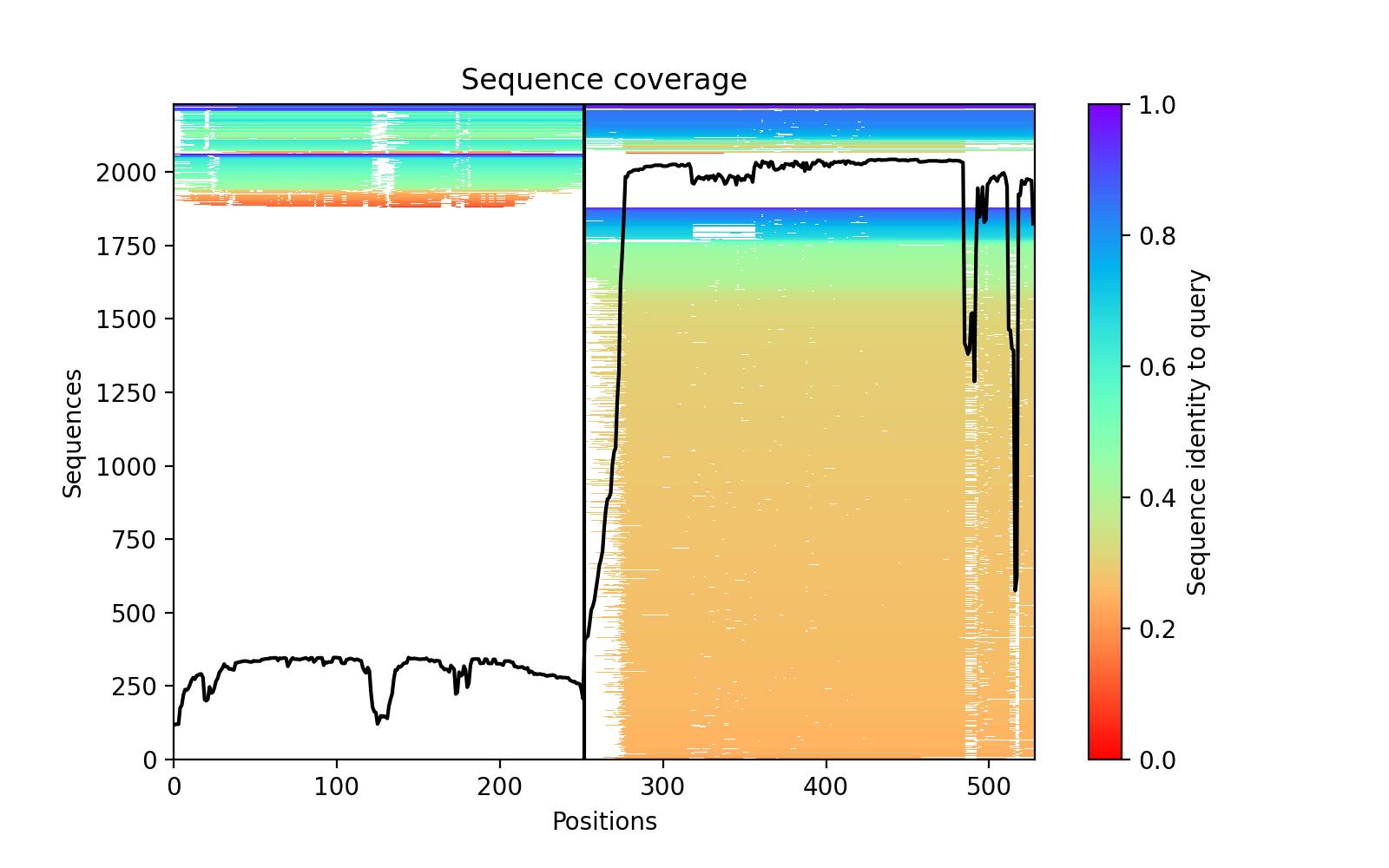

Supplement: Supplementary file 12 — Source Data Fig. 2 [file 44320_2024_19_MOESM12_ESM.zip › Source Data Figure EV3-EV4/ColabFold/OSM_ZNF688_coverage.png]

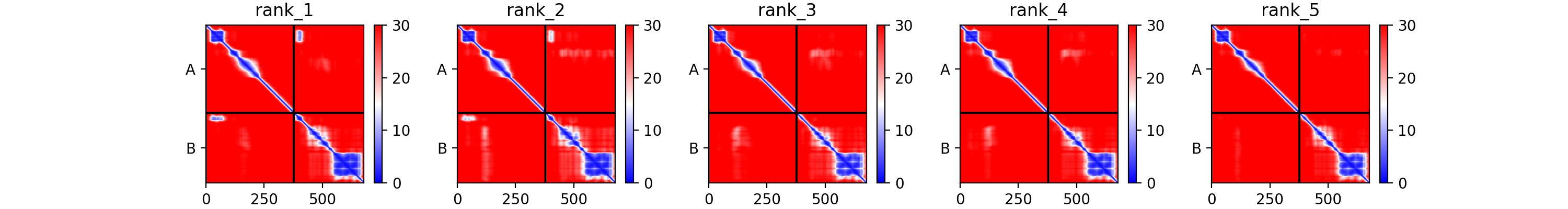

Supplement: Supplementary file 12 — Source Data Fig. 2 [file 44320_2024_19_MOESM12_ESM.zip › Source Data Figure EV3-EV4/ColabFold/PEX14_PEX19_PAE.png]

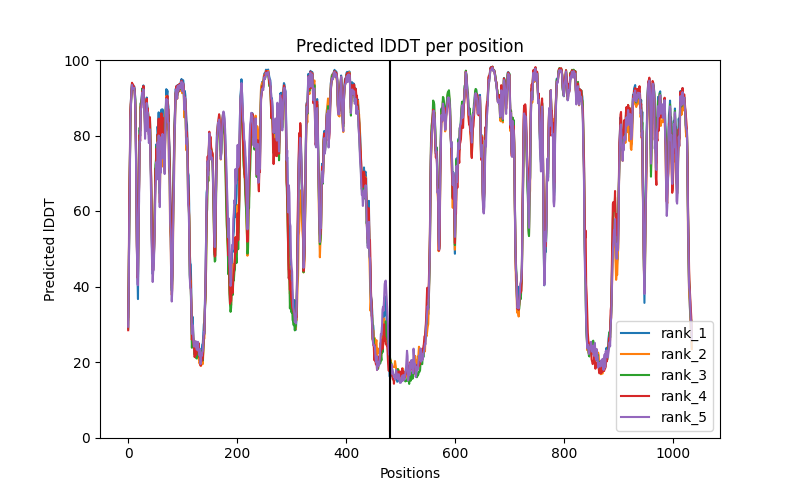

Supplement: Supplementary file 12 — Source Data Fig. 2 [file 44320_2024_19_MOESM12_ESM.zip › Source Data Figure EV3-EV4/ColabFold/AKT1_PDPK1_plddt.png]

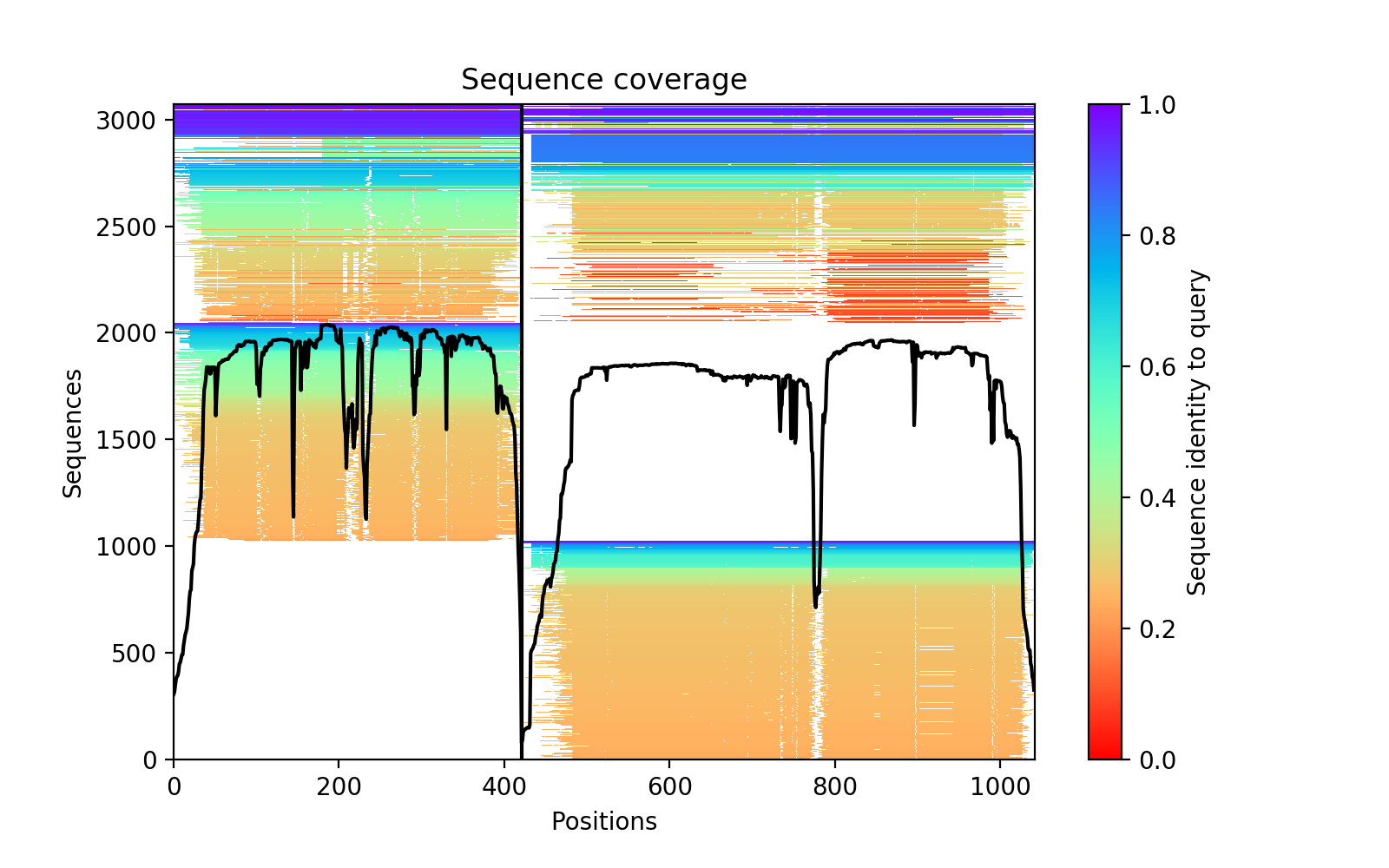

Supplement: Supplementary file 12 — Source Data Fig. 2 [file 44320_2024_19_MOESM12_ESM.zip › Source Data Figure EV3-EV4/ColabFold/RCC1_KLHL6_coverage.png]

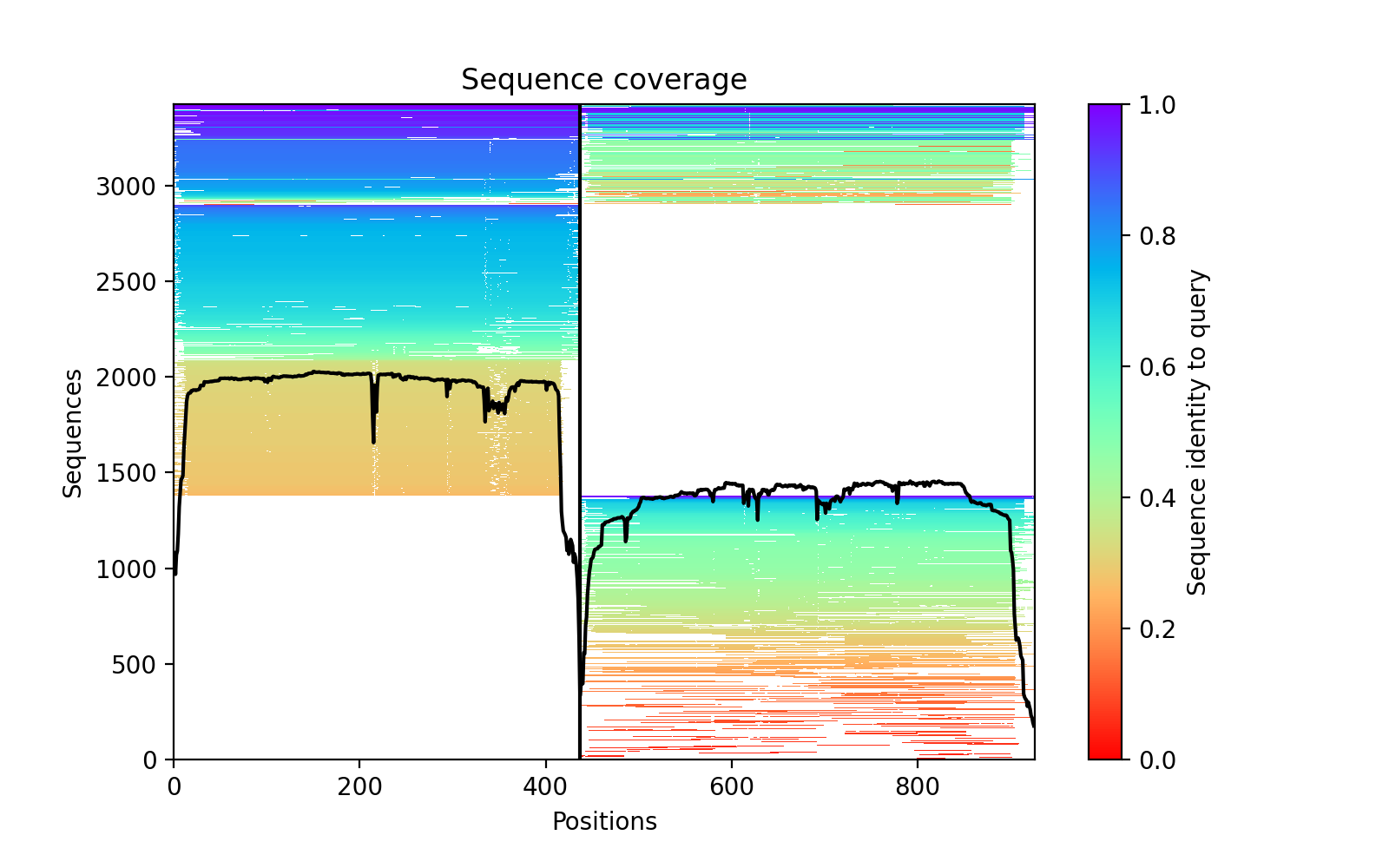

Supplement: Supplementary file 12 — Source Data Fig. 2 [file 44320_2024_19_MOESM12_ESM.zip › Source Data Figure EV3-EV4/ColabFold/ETF1_LMBR1L_coverage.png]

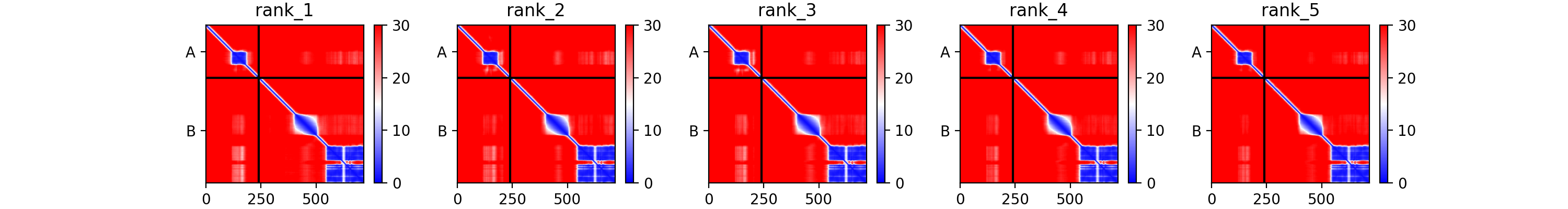

Supplement: Supplementary file 12 — Source Data Fig. 2 [file 44320_2024_19_MOESM12_ESM.zip › Source Data Figure EV3-EV4/ColabFold/DLX4_RAB3IP_PAE.png]

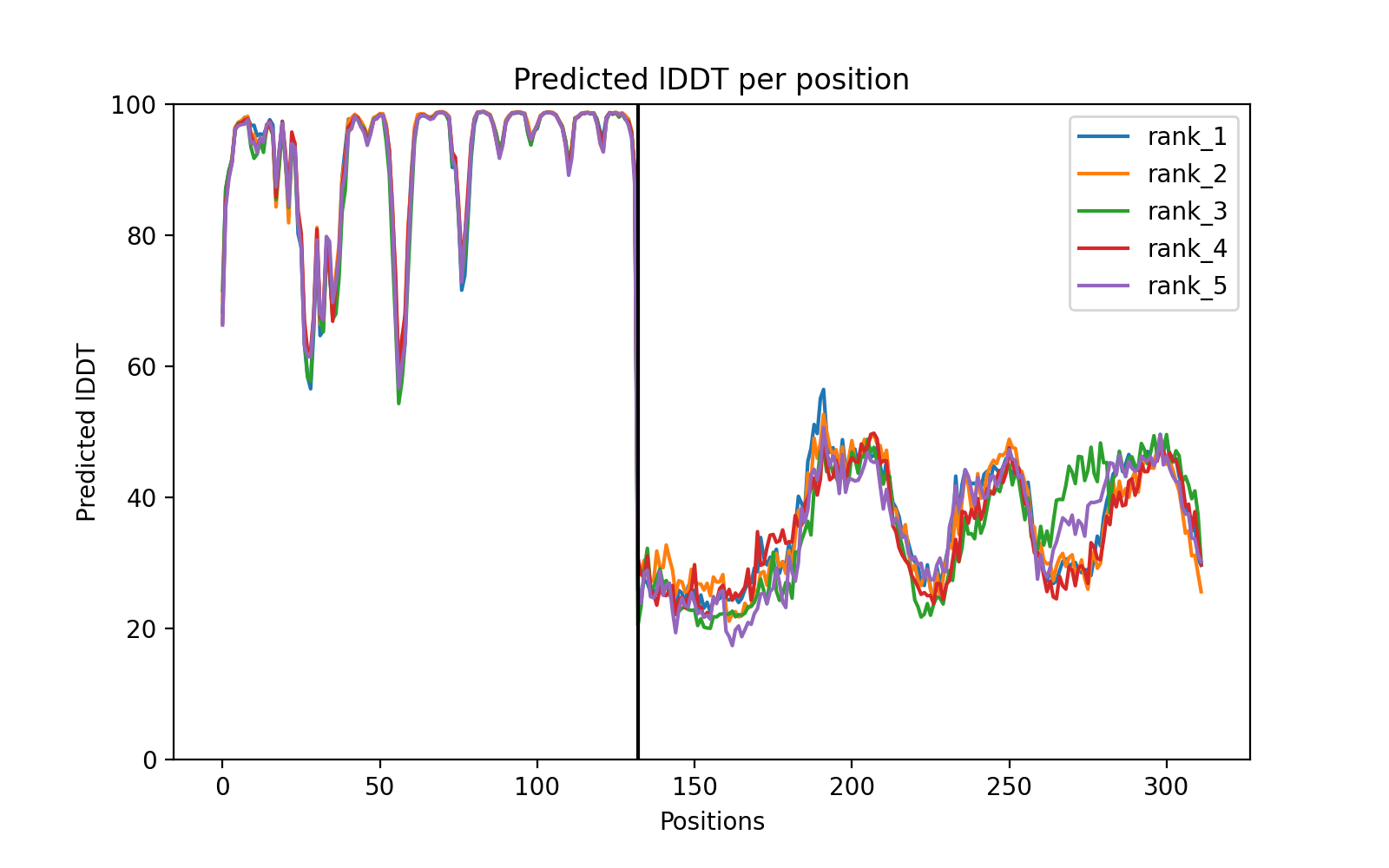

Supplement: Supplementary file 12 — Source Data Fig. 2 [file 44320_2024_19_MOESM12_ESM.zip › Source Data Figure EV3-EV4/ColabFold/FABP4_GCG_plddt.png]

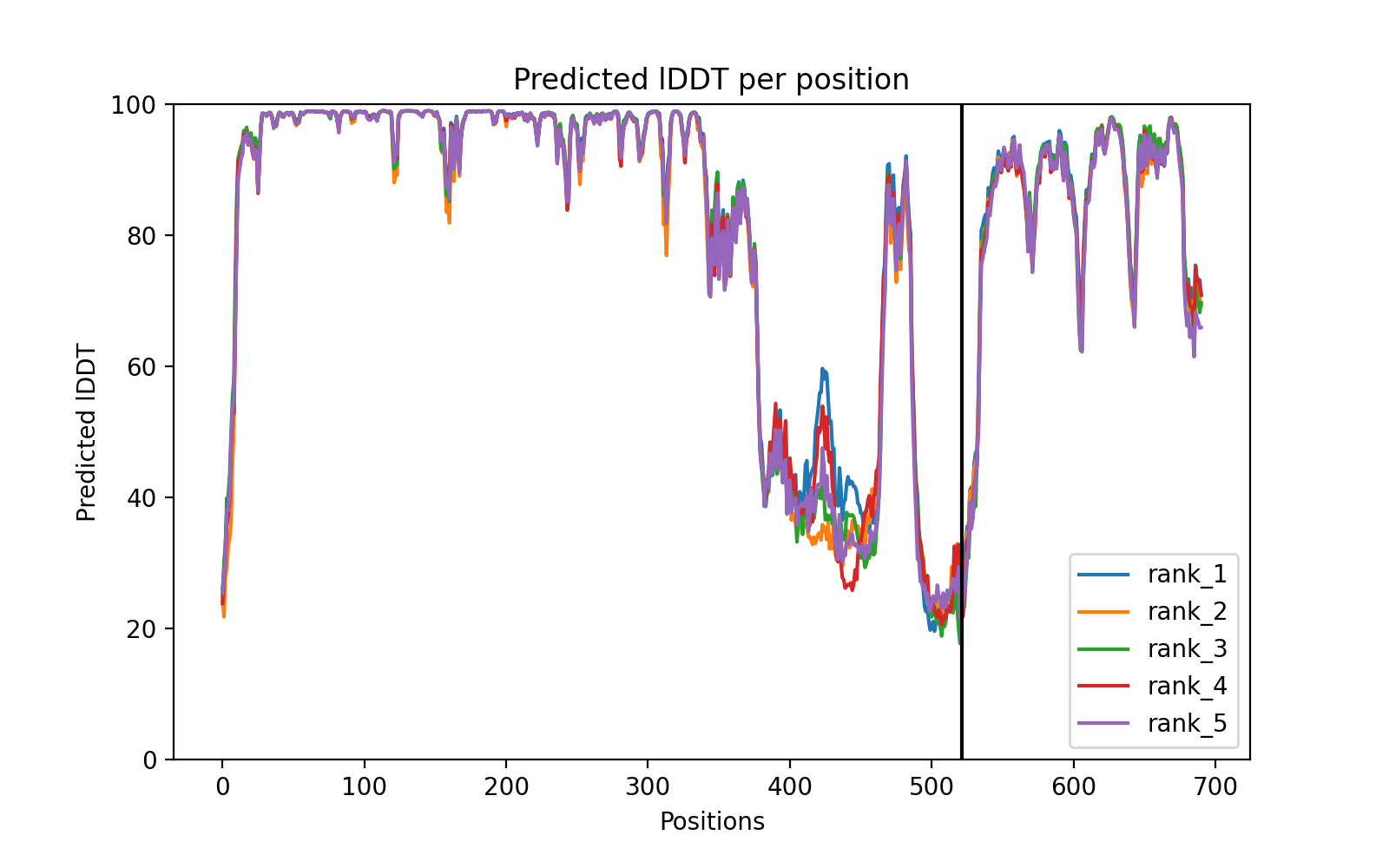

Supplement: Supplementary file 12 — Source Data Fig. 2 [file 44320_2024_19_MOESM12_ESM.zip › Source Data Figure EV3-EV4/ColabFold/PPP3CA_PPP3R1_plddt.png]

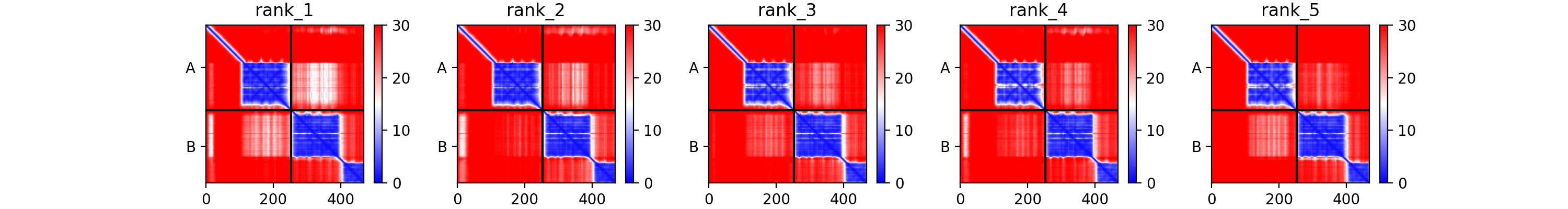

Supplement: Supplementary file 12 — Source Data Fig. 2 [file 44320_2024_19_MOESM12_ESM.zip › Source Data Figure EV3-EV4/ColabFold/DUT_C19orf40_PAE.png]

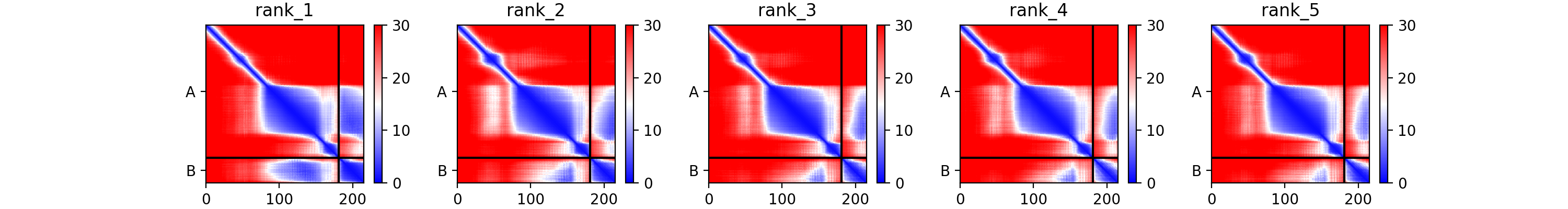

Supplement: Supplementary file 12 — Source Data Fig. 2 [file 44320_2024_19_MOESM12_ESM.zip › Source Data Figure EV3-EV4/ColabFold/ATF3_DDIT3_PAE.png]

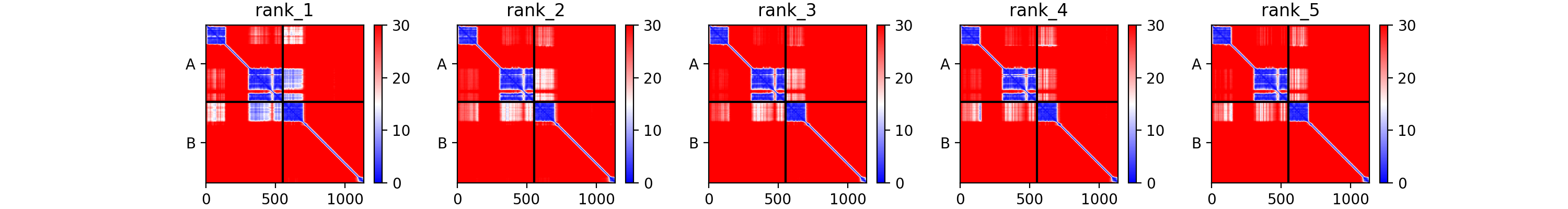

Supplement: Supplementary file 12 — Source Data Fig. 2 [file 44320_2024_19_MOESM12_ESM.zip › Source Data Figure EV3-EV4/ColabFold/SMAD4_DCP1A_PAE.png]

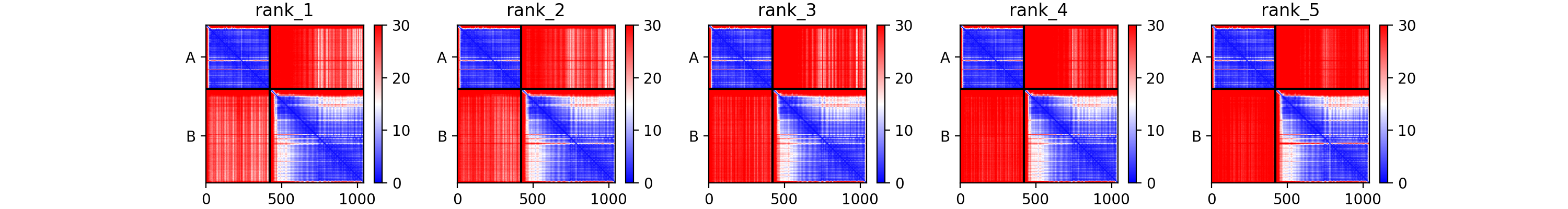

Supplement: Supplementary file 12 — Source Data Fig. 2 [file 44320_2024_19_MOESM12_ESM.zip › Source Data Figure EV3-EV4/ColabFold/RCC1_KLHL6_PAE.png]

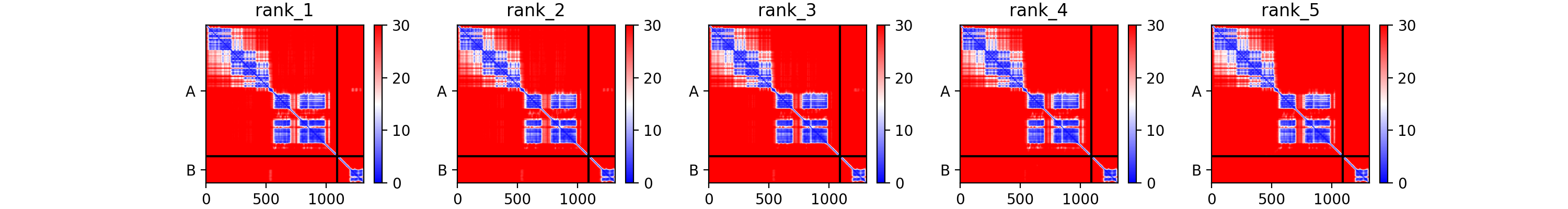

Supplement: Supplementary file 12 — Source Data Fig. 2 [file 44320_2024_19_MOESM12_ESM.zip › Source Data Figure EV3-EV4/ColabFold/PDGFRA_NDFIP1_PAE.png]

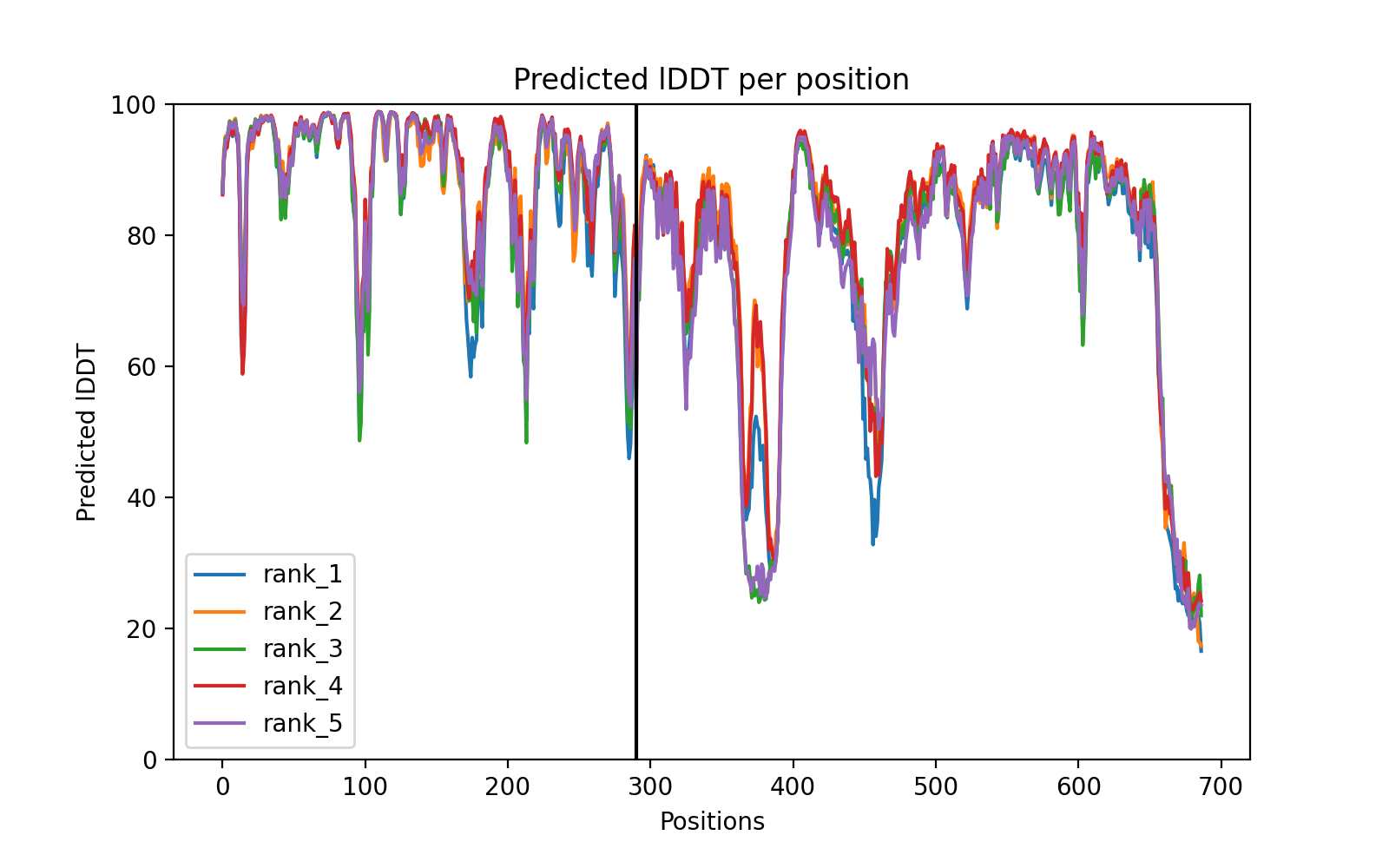

Supplement: Supplementary file 12 — Source Data Fig. 2 [file 44320_2024_19_MOESM12_ESM.zip › Source Data Figure EV3-EV4/ColabFold/NAT2_DNAJA1_plddt.png]

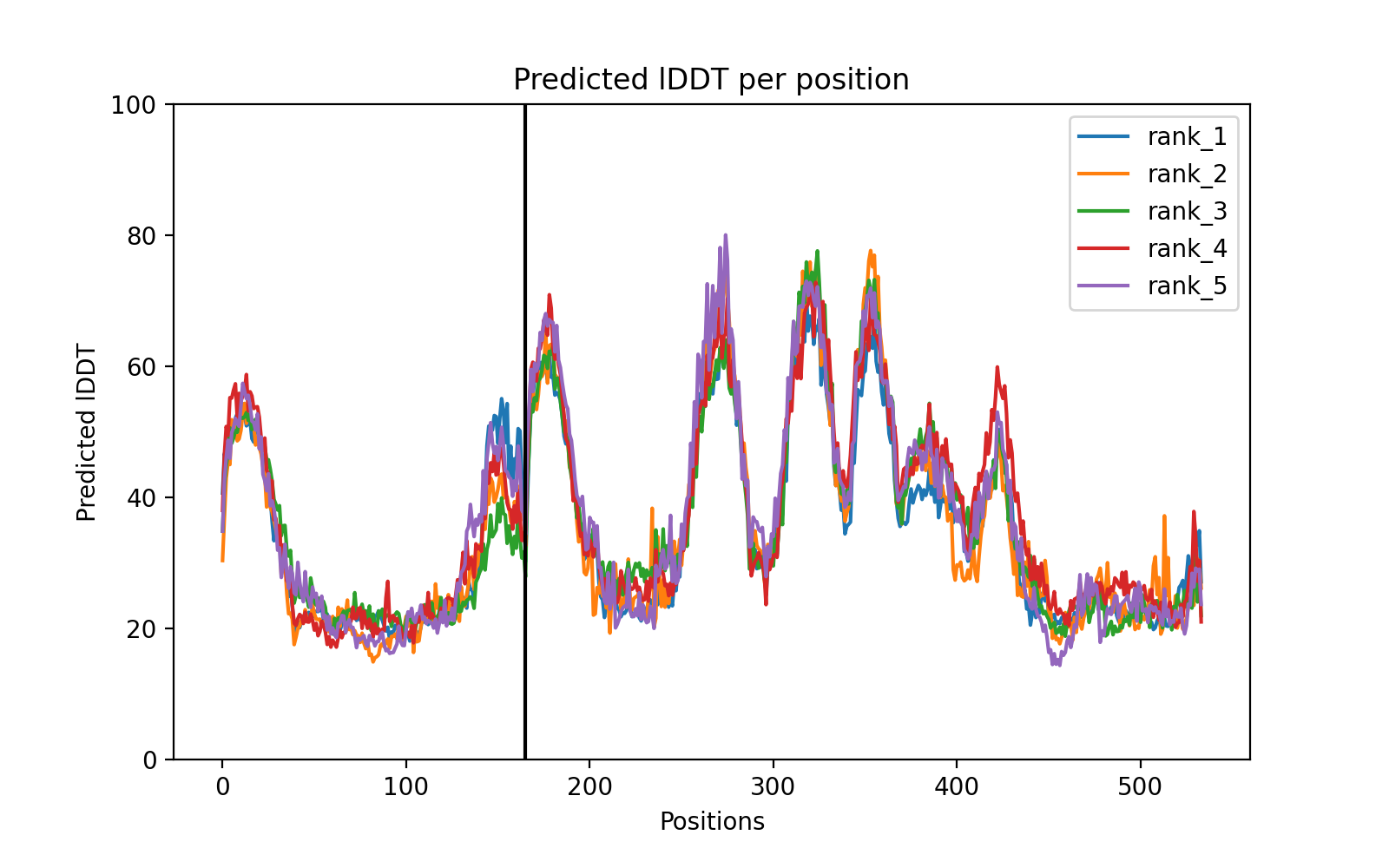

Supplement: Supplementary file 12 — Source Data Fig. 2 [file 44320_2024_19_MOESM12_ESM.zip › Source Data Figure EV3-EV4/ColabFold/PMCH_RIC3_plddt.png]

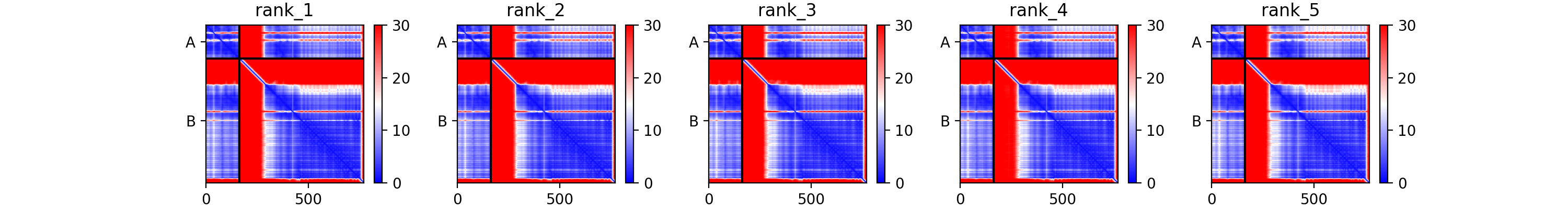

Supplement: Supplementary file 12 — Source Data Fig. 2 [file 44320_2024_19_MOESM12_ESM.zip › Source Data Figure EV3-EV4/ColabFold/SKP1_BTRC_PAE.png]

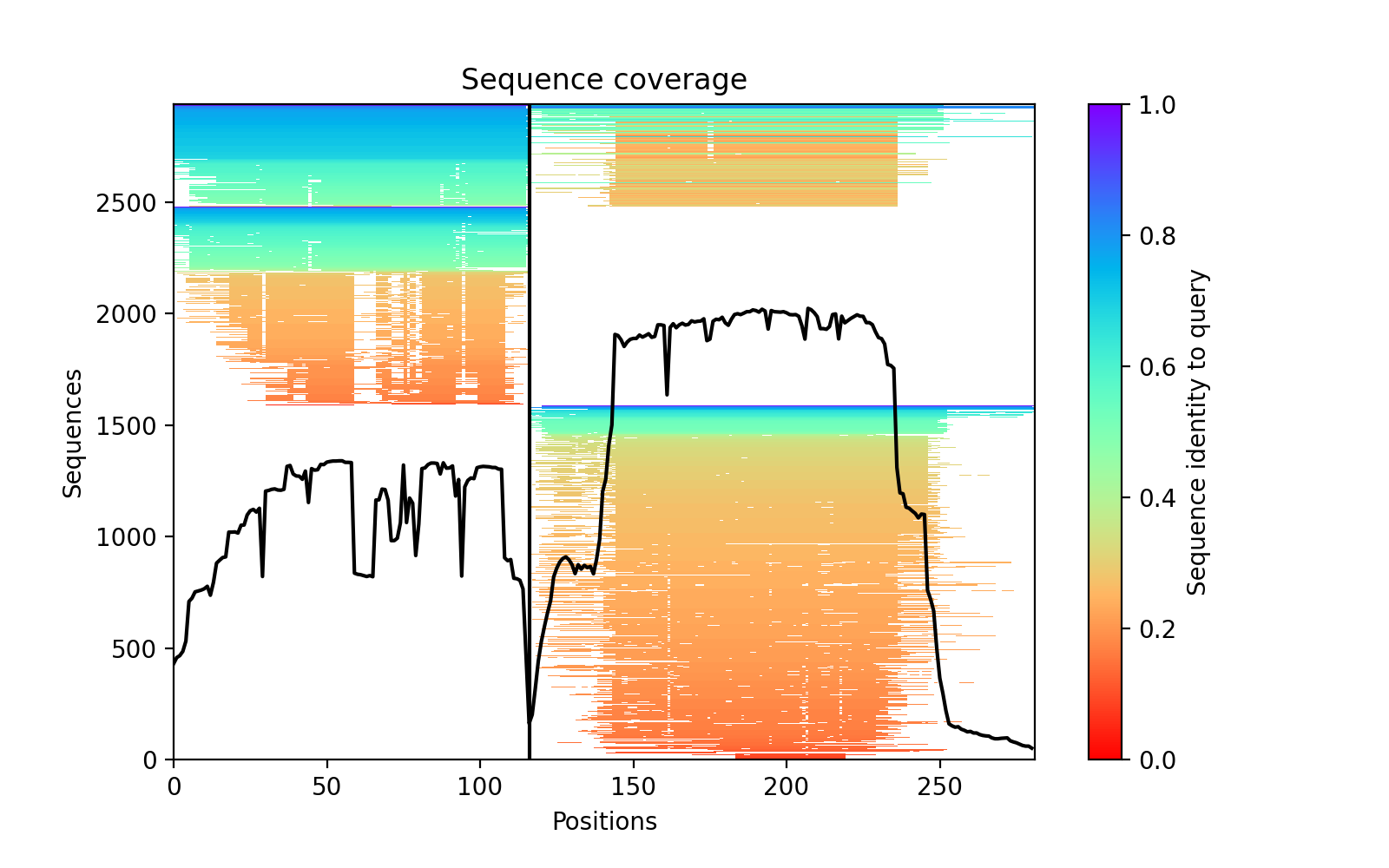

Supplement: Supplementary file 12 — Source Data Fig. 2 [file 44320_2024_19_MOESM12_ESM.zip › Source Data Figure EV3-EV4/ColabFold/CGA_CGB5_coverage.png]

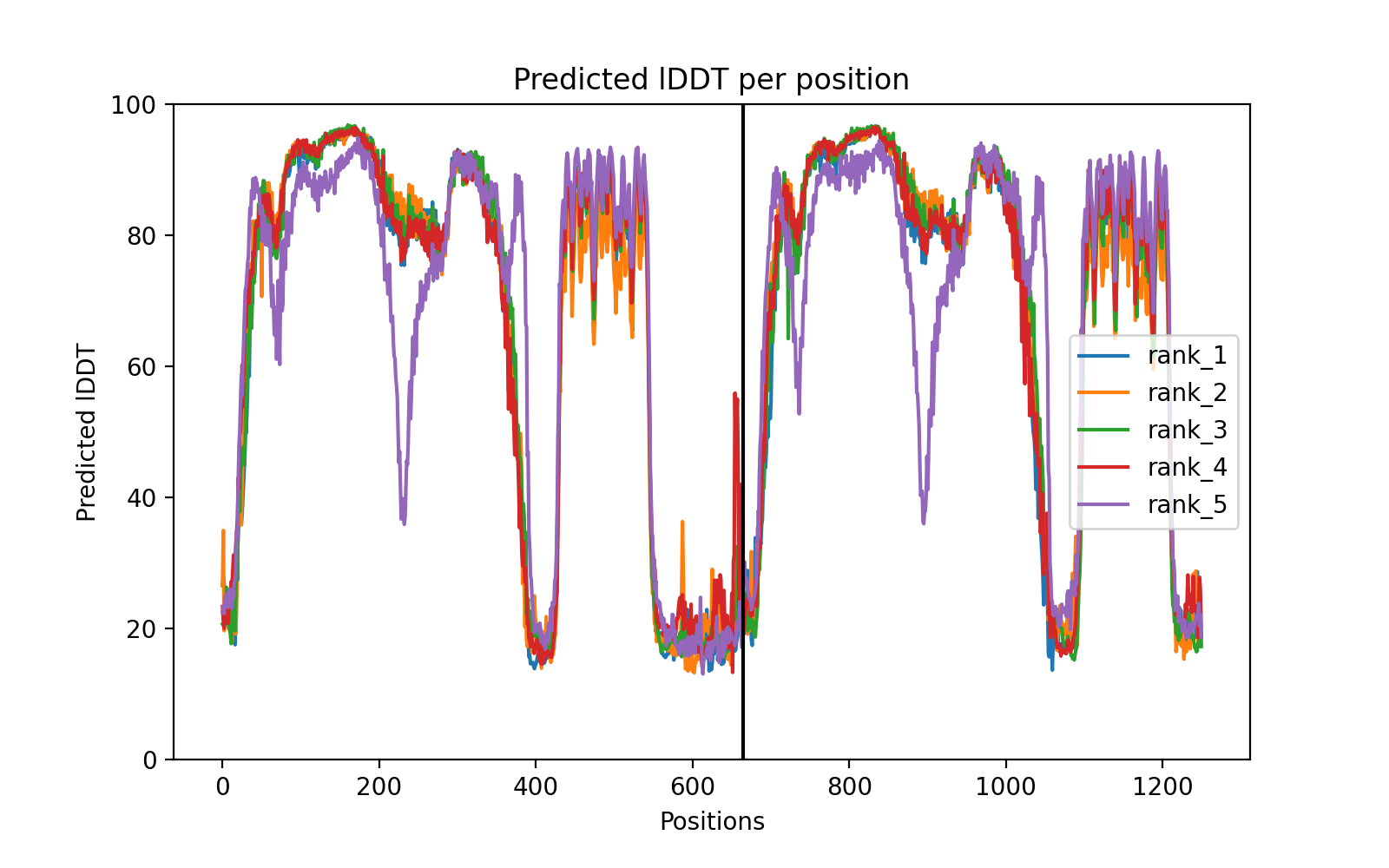

Supplement: Supplementary file 12 — Source Data Fig. 2 [file 44320_2024_19_MOESM12_ESM.zip › Source Data Figure EV3-EV4/ColabFold/LMNA_LMNB1_plddt.png]

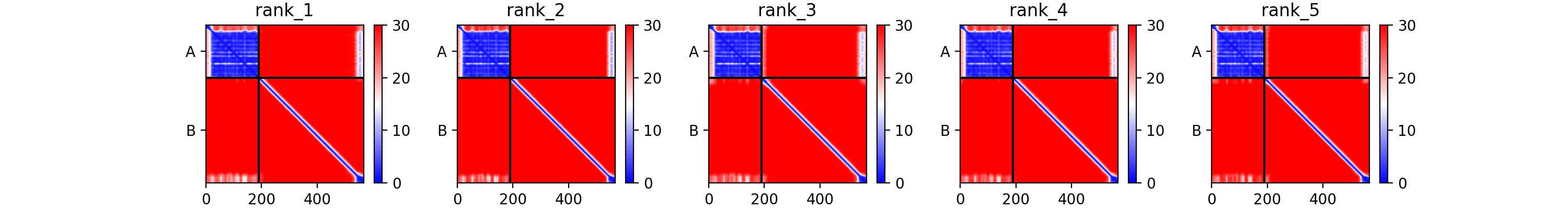

Supplement: Supplementary file 12 — Source Data Fig. 2 [file 44320_2024_19_MOESM12_ESM.zip › Source Data Figure EV3-EV4/ColabFold/APOD_MUC7_PAE.png]

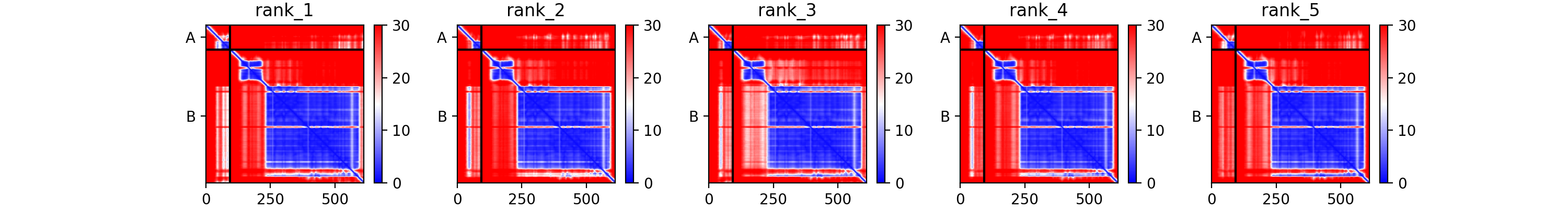

Supplement: Supplementary file 12 — Source Data Fig. 2 [file 44320_2024_19_MOESM12_ESM.zip › Source Data Figure EV3-EV4/ColabFold/DEFA3_TSTD2_PAE.png]

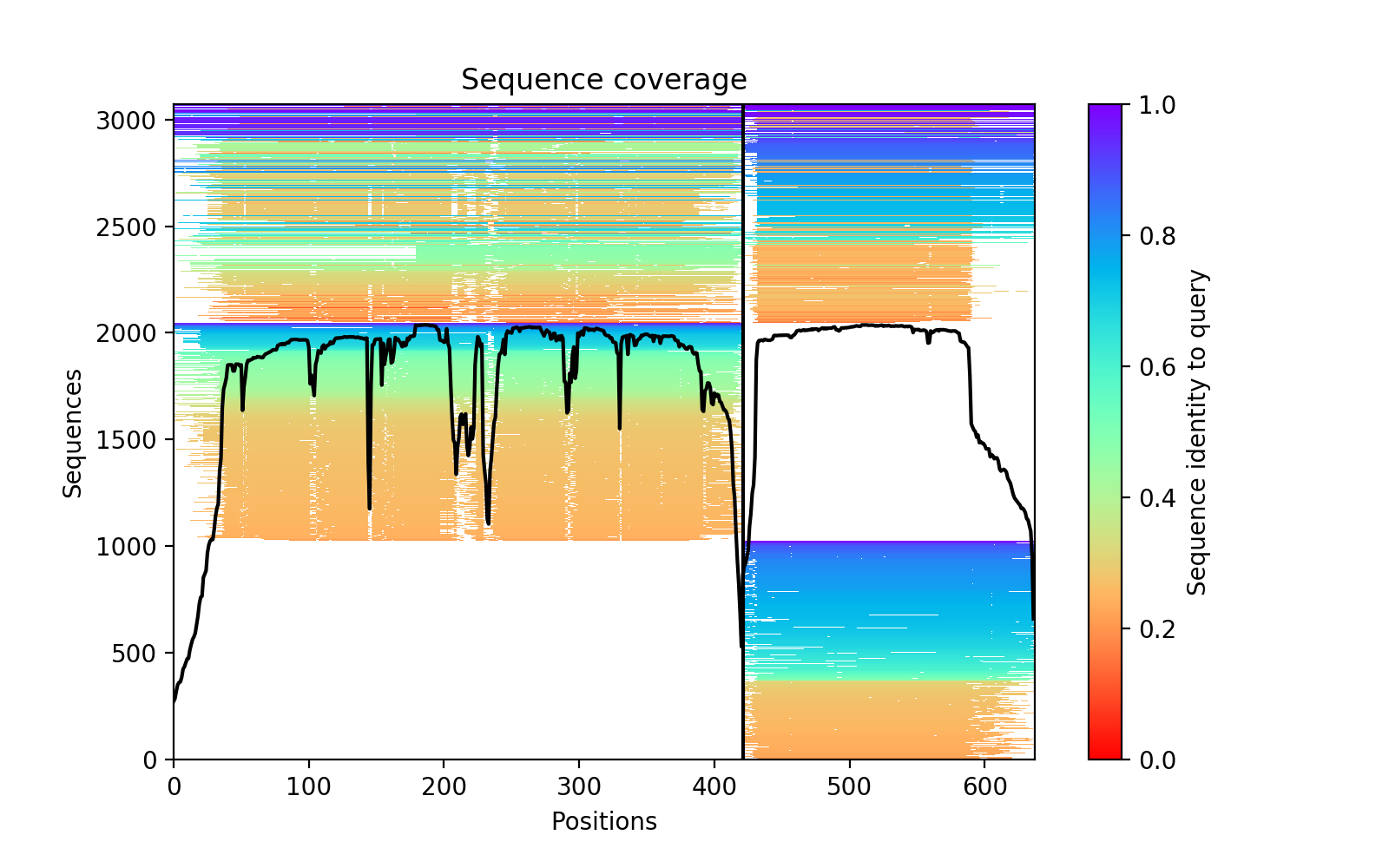

Supplement: Supplementary file 12 — Source Data Fig. 2 [file 44320_2024_19_MOESM12_ESM.zip › Source Data Figure EV3-EV4/ColabFold/RCC1_RAN_coverage.png]

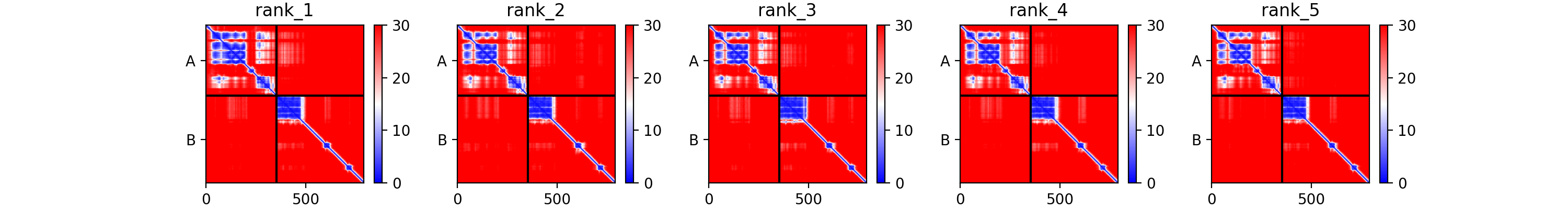

Supplement: Supplementary file 12 — Source Data Fig. 2 [file 44320_2024_19_MOESM12_ESM.zip › Source Data Figure EV3-EV4/ColabFold/FIGF_ZBTB25_PAE.png]

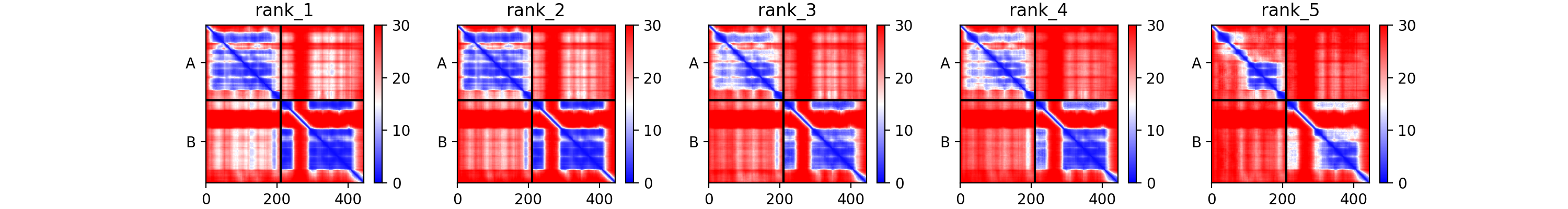

Supplement: Supplementary file 12 — Source Data Fig. 2 [file 44320_2024_19_MOESM12_ESM.zip › Source Data Figure EV3-EV4/ColabFold/BAK1_BCL2L1_PAE.png]

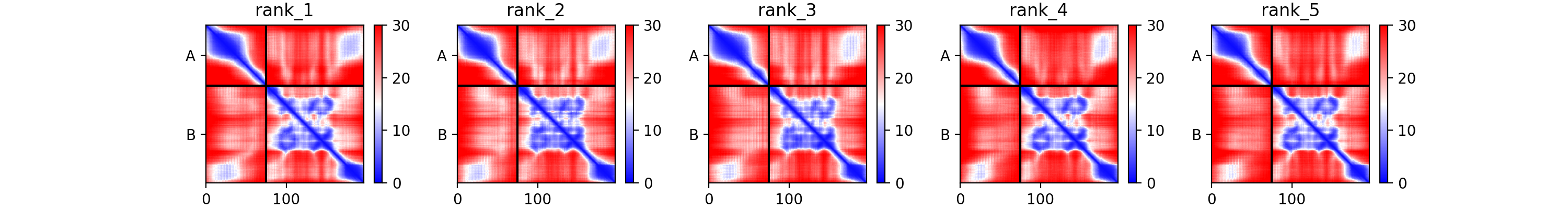

Supplement: Supplementary file 15 — Source Data Fig. 5 [file 44320_2024_19_MOESM15_ESM.zip › Source Data Figure 5/ColabFold/E-ORF7a_764d1.result/E_ORF7a_764d1_PAE.png]

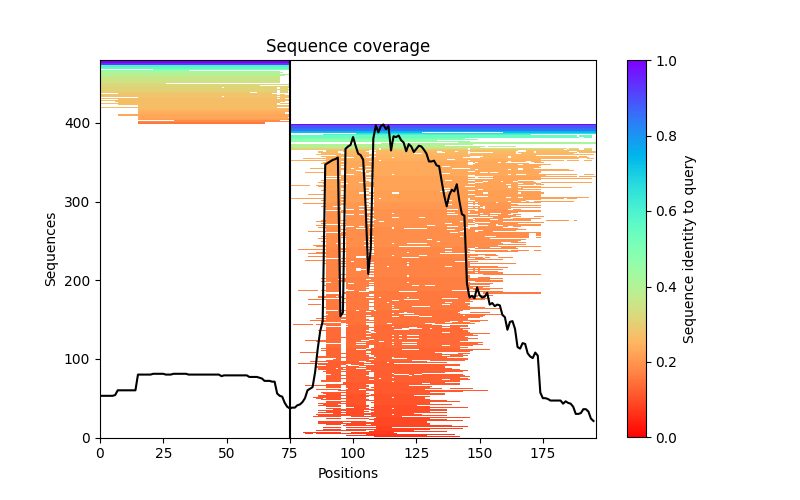

Supplement: Supplementary file 15 — Source Data Fig. 5 [file 44320_2024_19_MOESM15_ESM.zip › Source Data Figure 5/ColabFold/E-ORF7a_764d1.result/E_ORF7a_764d1_coverage.png]

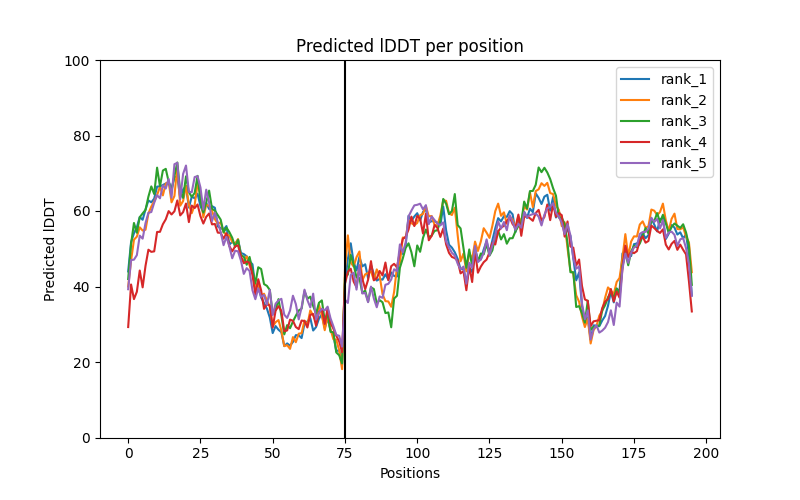

Supplement: Supplementary file 15 — Source Data Fig. 5 [file 44320_2024_19_MOESM15_ESM.zip › Source Data Figure 5/ColabFold/E-ORF7a_764d1.result/E_ORF7a_764d1_plddt.png]

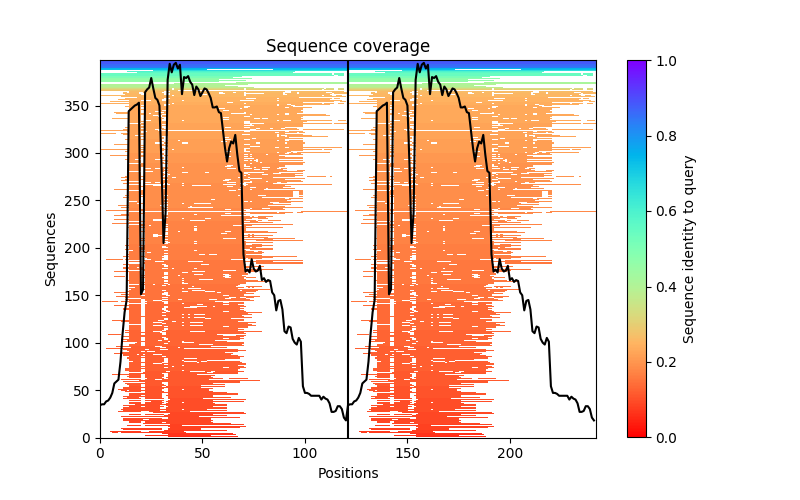

Supplement: Supplementary file 15 — Source Data Fig. 5 [file 44320_2024_19_MOESM15_ESM.zip › Source Data Figure 5/ColabFold/ORF7a-ORF7a_b4818.result/ORF7a_ORF7a_b4818_coverage.png]

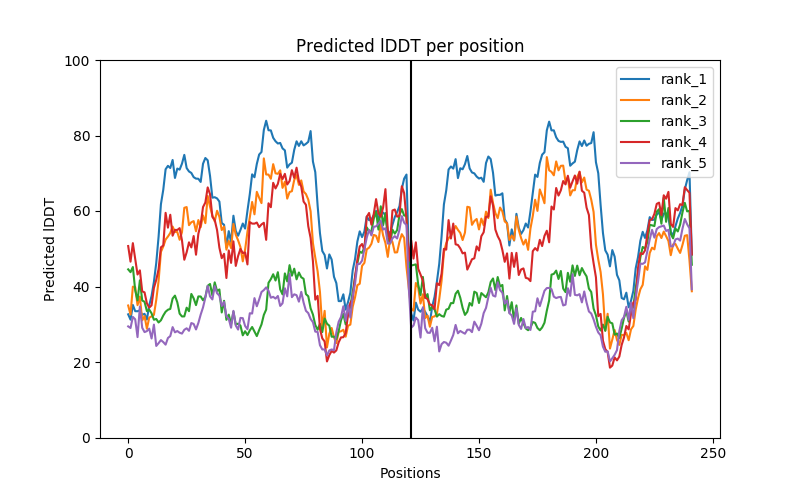

Supplement: Supplementary file 15 — Source Data Fig. 5 [file 44320_2024_19_MOESM15_ESM.zip › Source Data Figure 5/ColabFold/ORF7a-ORF7a_b4818.result/ORF7a_ORF7a_b4818_plddt.png]

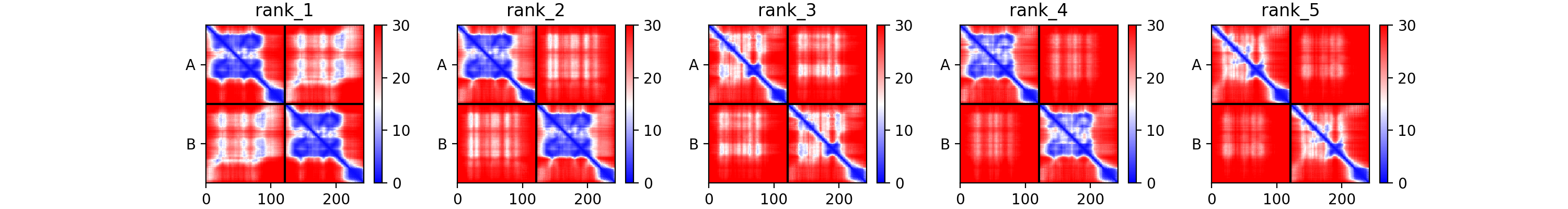

Supplement: Supplementary file 15 — Source Data Fig. 5 [file 44320_2024_19_MOESM15_ESM.zip › Source Data Figure 5/ColabFold/ORF7a-ORF7a_b4818.result/ORF7a_ORF7a_b4818_PAE.png]

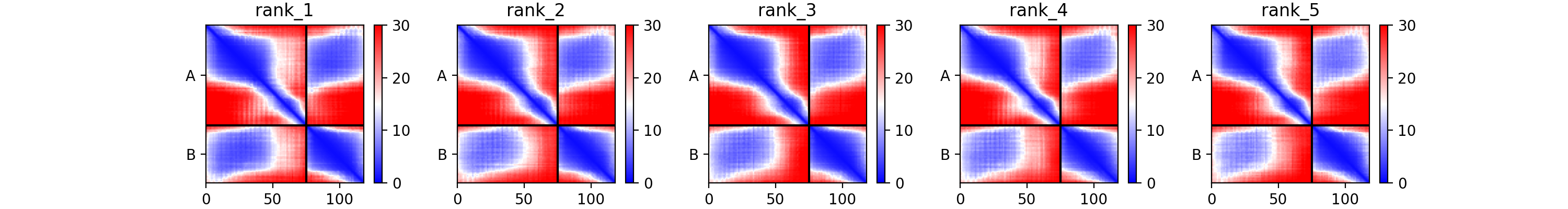

Supplement: Supplementary file 15 — Source Data Fig. 5 [file 44320_2024_19_MOESM15_ESM.zip › Source Data Figure 5/ColabFold/E-ORF7b_a7aff.result/E_ORF7b_a7aff_PAE.png]

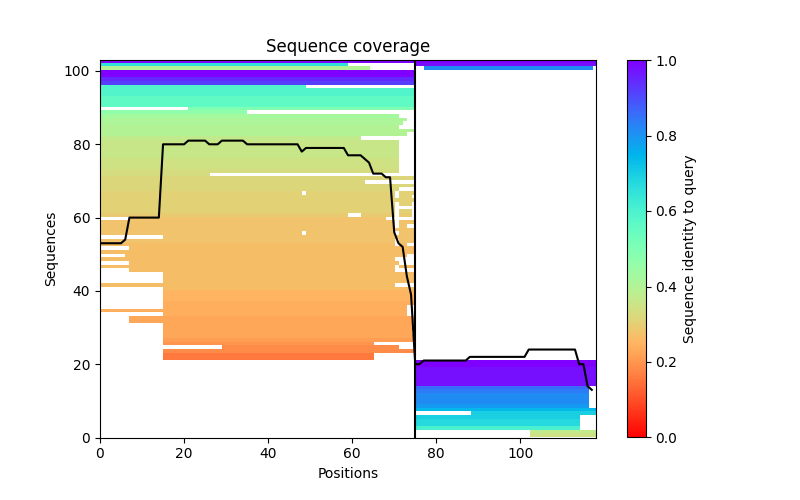

Supplement: Supplementary file 15 — Source Data Fig. 5 [file 44320_2024_19_MOESM15_ESM.zip › Source Data Figure 5/ColabFold/E-ORF7b_a7aff.result/E_ORF7b_a7aff_coverage.png]

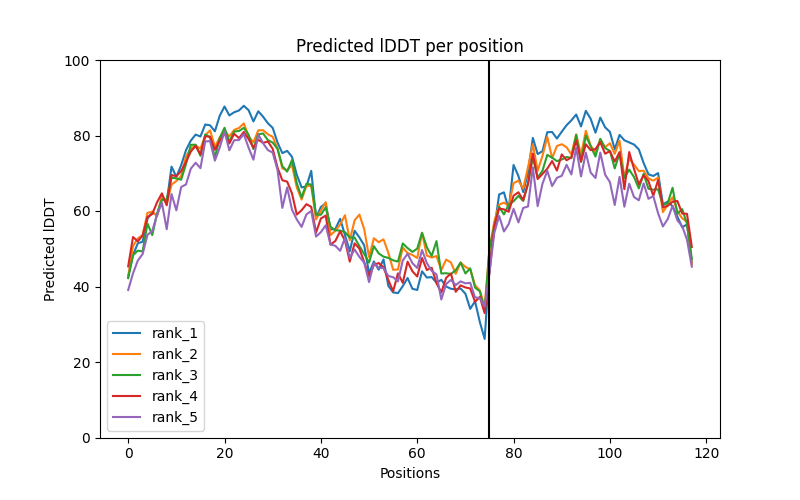

Supplement: Supplementary file 15 — Source Data Fig. 5 [file 44320_2024_19_MOESM15_ESM.zip › Source Data Figure 5/ColabFold/E-ORF7b_a7aff.result/E_ORF7b_a7aff_plddt.png]

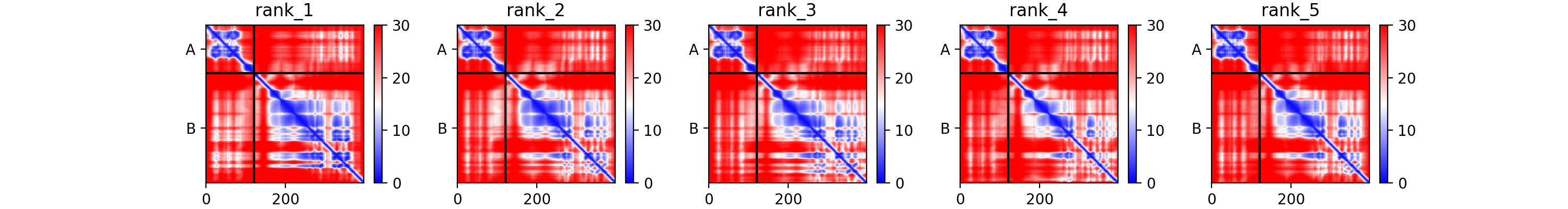

Supplement: Supplementary file 15 — Source Data Fig. 5 [file 44320_2024_19_MOESM15_ESM.zip › Source Data Figure 5/ColabFold/ORF7a-ORF3a_5fed4.result/ORF7a_ORF3a_5fed4_PAE.png]

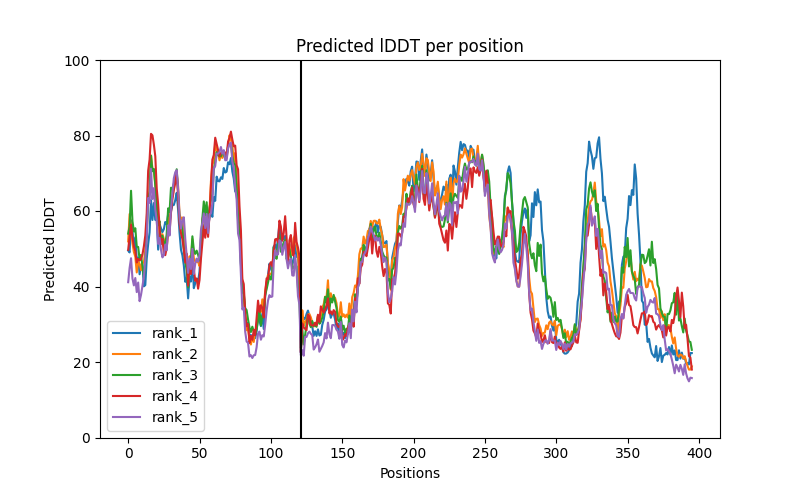

Supplement: Supplementary file 15 — Source Data Fig. 5 [file 44320_2024_19_MOESM15_ESM.zip › Source Data Figure 5/ColabFold/ORF7a-ORF3a_5fed4.result/ORF7a_ORF3a_5fed4_plddt.png]

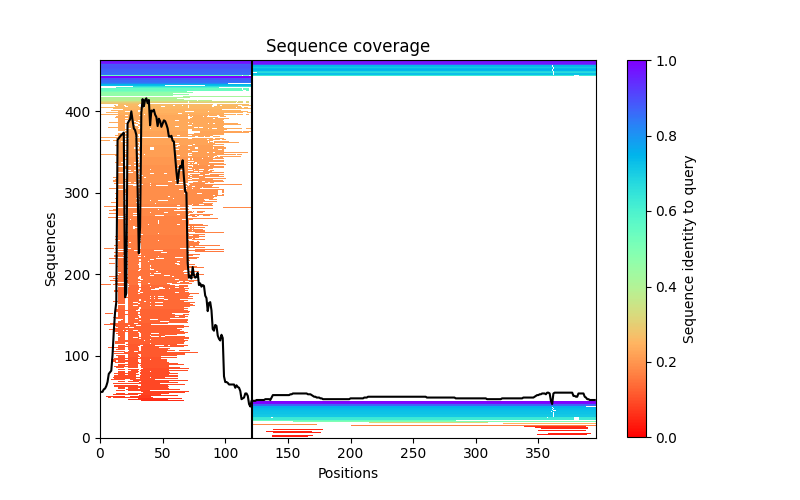

Supplement: Supplementary file 15 — Source Data Fig. 5 [file 44320_2024_19_MOESM15_ESM.zip › Source Data Figure 5/ColabFold/ORF7a-ORF3a_5fed4.result/ORF7a_ORF3a_5fed4_coverage.png]

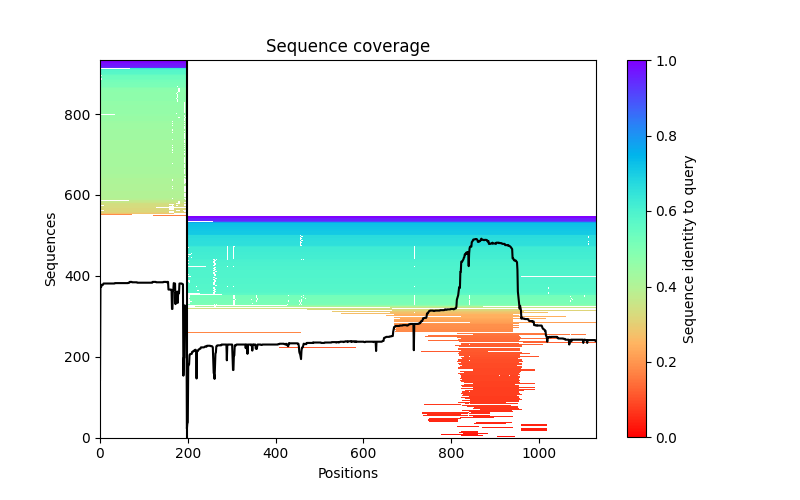

Supplement: Supplementary file 15 — Source Data Fig. 5 [file 44320_2024_19_MOESM15_ESM.zip › Source Data Figure 5/ColabFold/NSP8-NSP12_db995.result/NSP8_NSP12_db995_coverage.png]

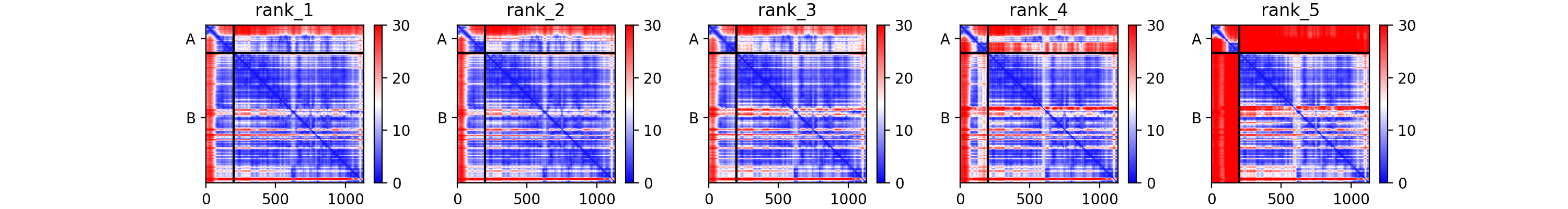

Supplement: Supplementary file 15 — Source Data Fig. 5 [file 44320_2024_19_MOESM15_ESM.zip › Source Data Figure 5/ColabFold/NSP8-NSP12_db995.result/NSP8_NSP12_db995_PAE.png]

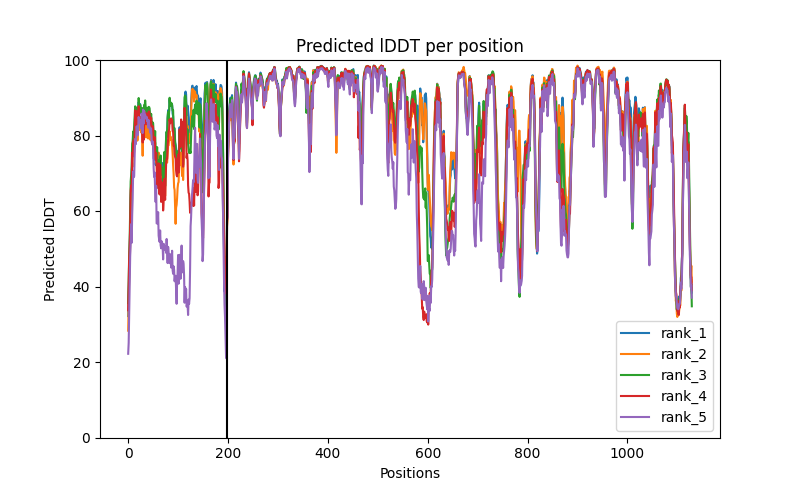

Supplement: Supplementary file 15 — Source Data Fig. 5 [file 44320_2024_19_MOESM15_ESM.zip › Source Data Figure 5/ColabFold/NSP8-NSP12_db995.result/NSP8_NSP12_db995_plddt.png]

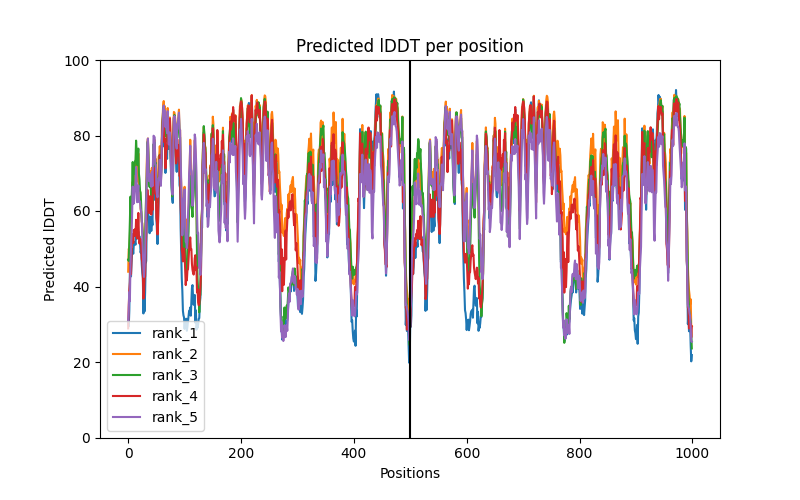

Supplement: Supplementary file 15 — Source Data Fig. 5 [file 44320_2024_19_MOESM15_ESM.zip › Source Data Figure 5/ColabFold/NSP4-NSP4_8f98f.result/NSP4_NSP4_8f98f_plddt.png]

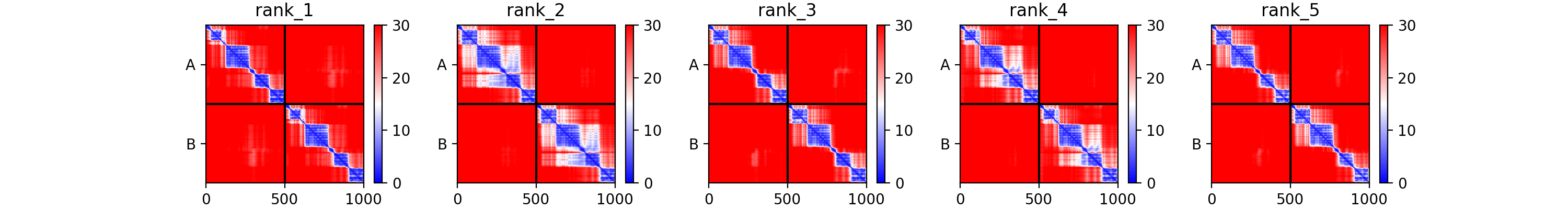

Supplement: Supplementary file 15 — Source Data Fig. 5 [file 44320_2024_19_MOESM15_ESM.zip › Source Data Figure 5/ColabFold/NSP4-NSP4_8f98f.result/NSP4_NSP4_8f98f_PAE.png]

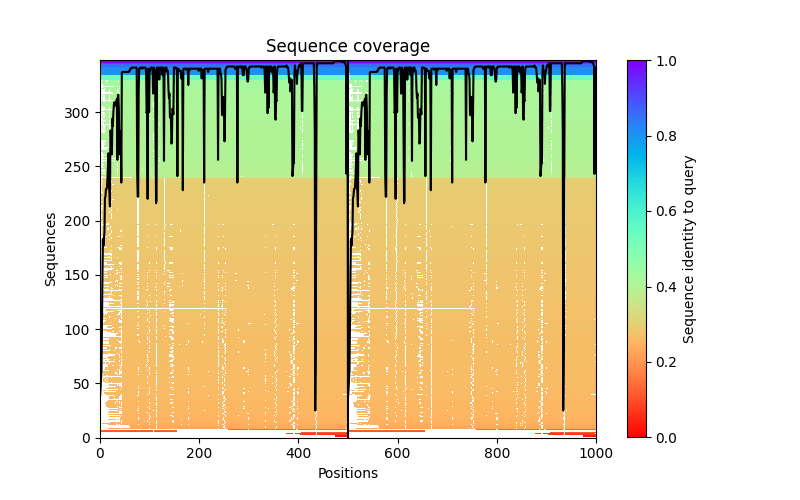

Supplement: Supplementary file 15 — Source Data Fig. 5 [file 44320_2024_19_MOESM15_ESM.zip › Source Data Figure 5/ColabFold/NSP4-NSP4_8f98f.result/NSP4_NSP4_8f98f_coverage.png]

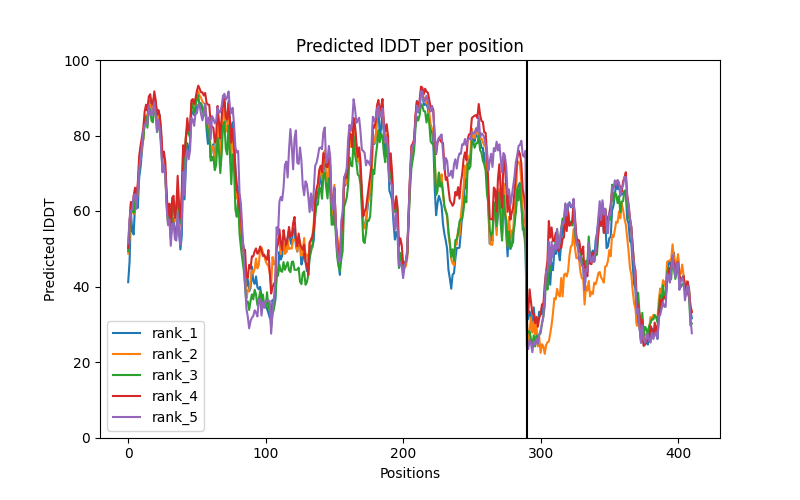

Supplement: Supplementary file 15 — Source Data Fig. 5 [file 44320_2024_19_MOESM15_ESM.zip › Source Data Figure 5/ColabFold/NSP6-ORF7a_43e6e.result/NSP6_ORF7a_43e6e_plddt.png]

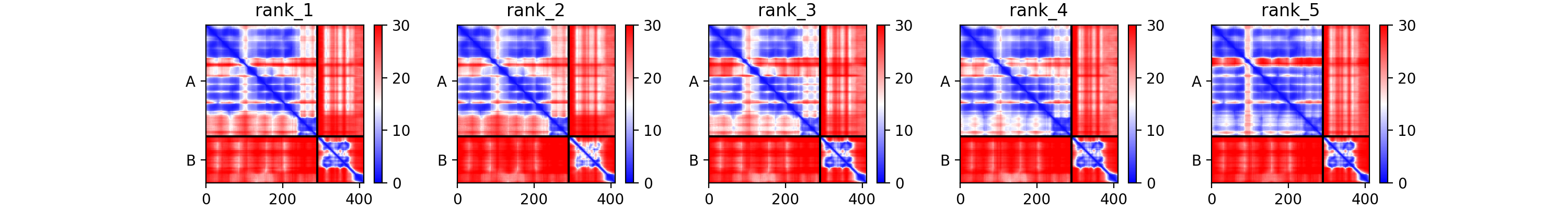

Supplement: Supplementary file 15 — Source Data Fig. 5 [file 44320_2024_19_MOESM15_ESM.zip › Source Data Figure 5/ColabFold/NSP6-ORF7a_43e6e.result/NSP6_ORF7a_43e6e_PAE.png]

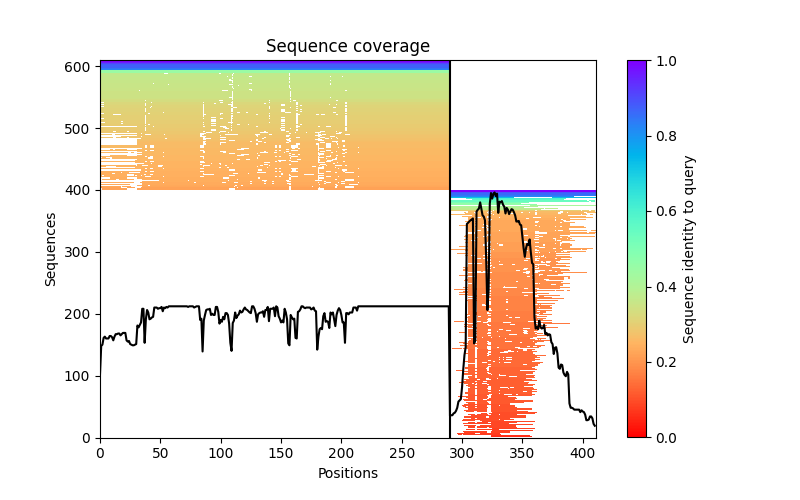

Supplement: Supplementary file 15 — Source Data Fig. 5 [file 44320_2024_19_MOESM15_ESM.zip › Source Data Figure 5/ColabFold/NSP6-ORF7a_43e6e.result/NSP6_ORF7a_43e6e_coverage.png]

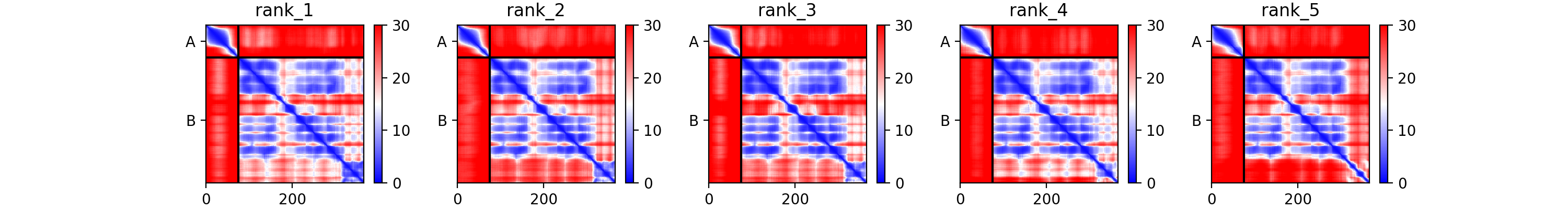

Supplement: Supplementary file 15 — Source Data Fig. 5 [file 44320_2024_19_MOESM15_ESM.zip › Source Data Figure 5/ColabFold/E-NSP6_9c8a7.result/E_NSP6_9c8a7_PAE.png]

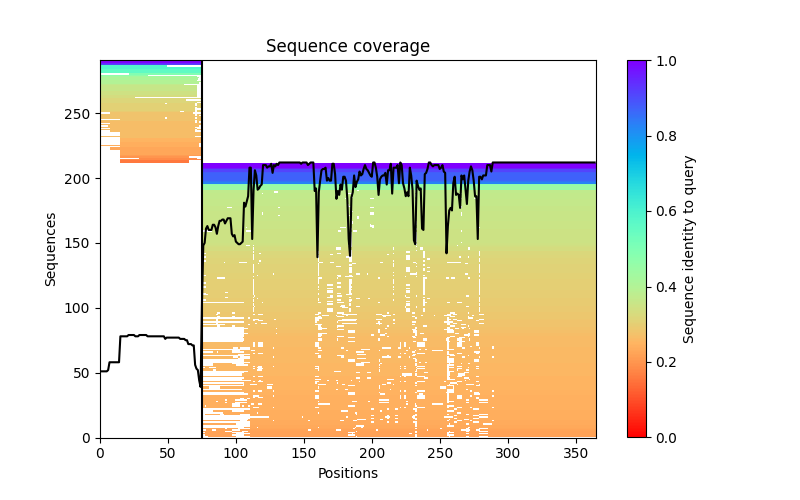

Supplement: Supplementary file 15 — Source Data Fig. 5 [file 44320_2024_19_MOESM15_ESM.zip › Source Data Figure 5/ColabFold/E-NSP6_9c8a7.result/E_NSP6_9c8a7_coverage.png]

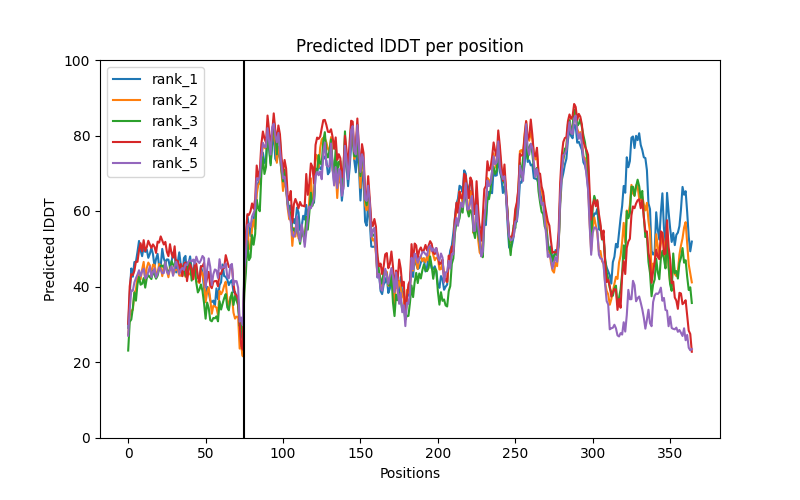

Supplement: Supplementary file 15 — Source Data Fig. 5 [file 44320_2024_19_MOESM15_ESM.zip › Source Data Figure 5/ColabFold/E-NSP6_9c8a7.result/E_NSP6_9c8a7_plddt.png]

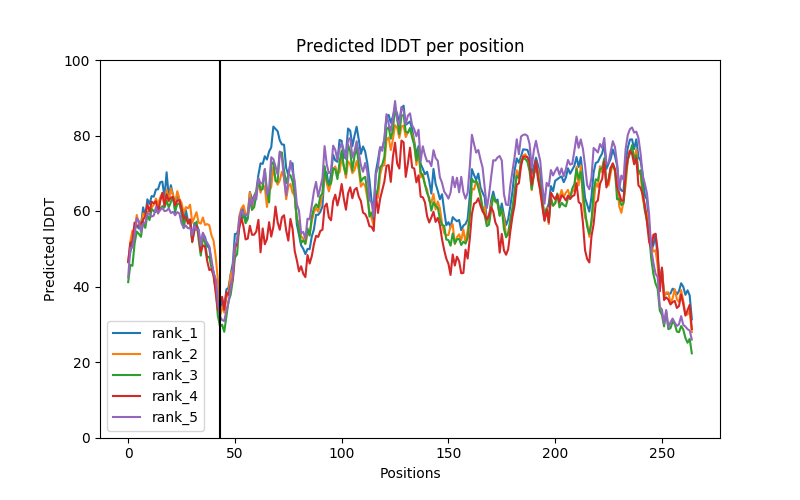

Supplement: Supplementary file 15 — Source Data Fig. 5 [file 44320_2024_19_MOESM15_ESM.zip › Source Data Figure 5/ColabFold/ORF7b-M_904cd.result/ORF7b_M_904cd_plddt.png]

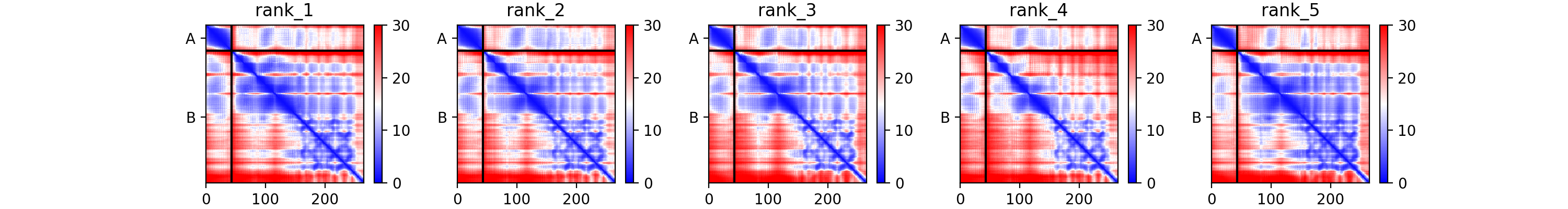

Supplement: Supplementary file 15 — Source Data Fig. 5 [file 44320_2024_19_MOESM15_ESM.zip › Source Data Figure 5/ColabFold/ORF7b-M_904cd.result/ORF7b_M_904cd_PAE.png]

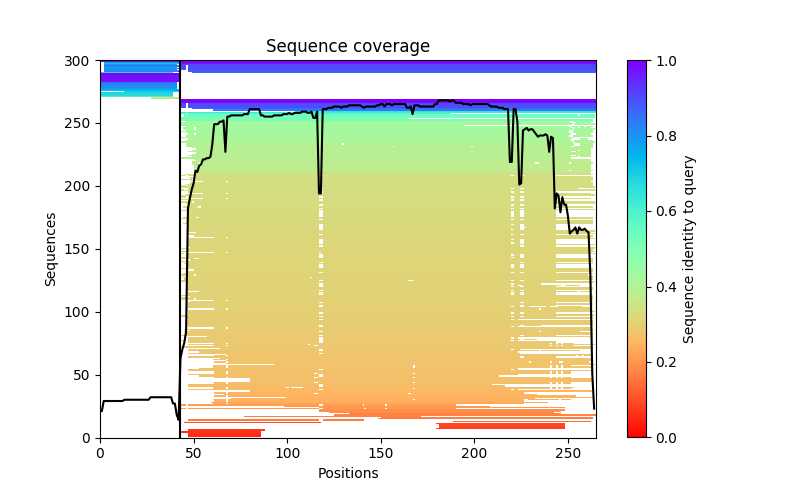

Supplement: Supplementary file 15 — Source Data Fig. 5 [file 44320_2024_19_MOESM15_ESM.zip › Source Data Figure 5/ColabFold/ORF7b-M_904cd.result/ORF7b_M_904cd_coverage.png]

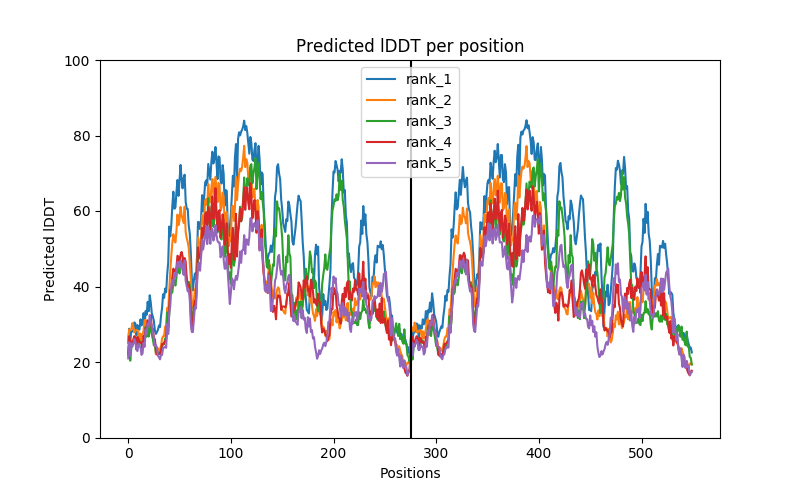

Supplement: Supplementary file 15 — Source Data Fig. 5 [file 44320_2024_19_MOESM15_ESM.zip › Source Data Figure 5/ColabFold/ORF3a-ORF3a_4d856.result/ORF3a_ORF3a_4d856_plddt.png]

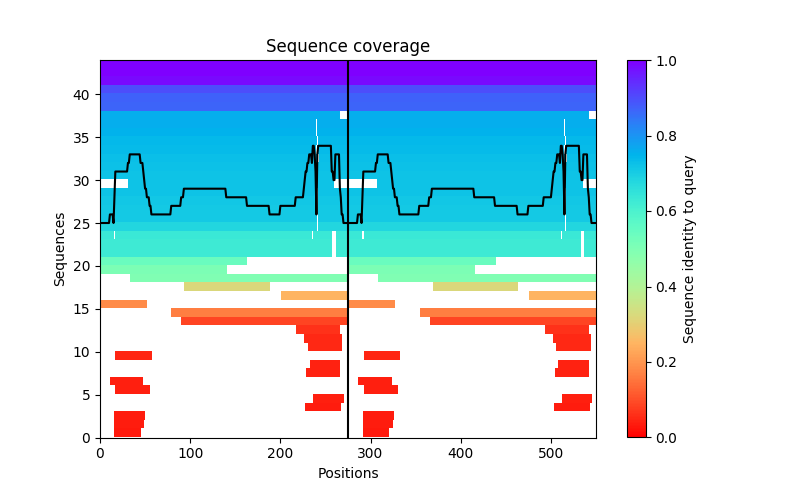

Supplement: Supplementary file 15 — Source Data Fig. 5 [file 44320_2024_19_MOESM15_ESM.zip › Source Data Figure 5/ColabFold/ORF3a-ORF3a_4d856.result/ORF3a_ORF3a_4d856_coverage.png]

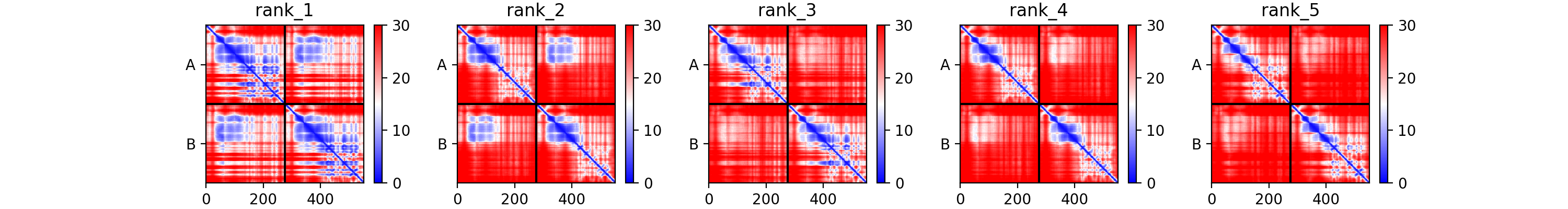

Supplement: Supplementary file 15 — Source Data Fig. 5 [file 44320_2024_19_MOESM15_ESM.zip › Source Data Figure 5/ColabFold/ORF3a-ORF3a_4d856.result/ORF3a_ORF3a_4d856_PAE.png]

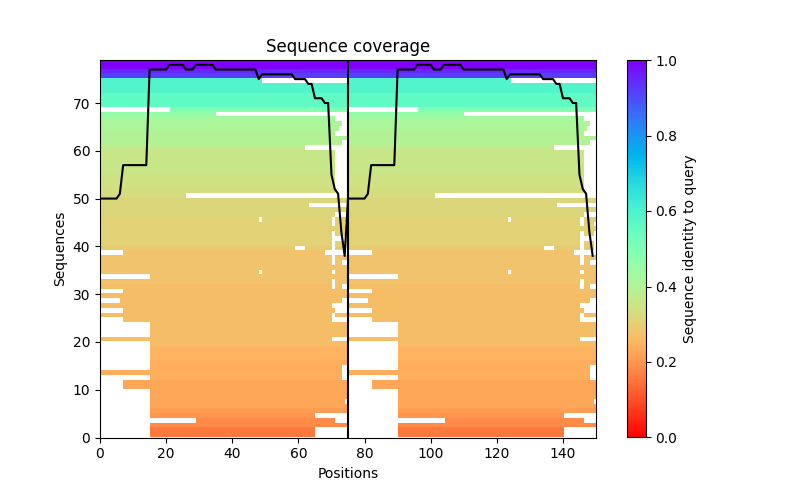

Supplement: Supplementary file 15 — Source Data Fig. 5 [file 44320_2024_19_MOESM15_ESM.zip › Source Data Figure 5/ColabFold/E-E_420f7.result/E_E_420f7_coverage.png]

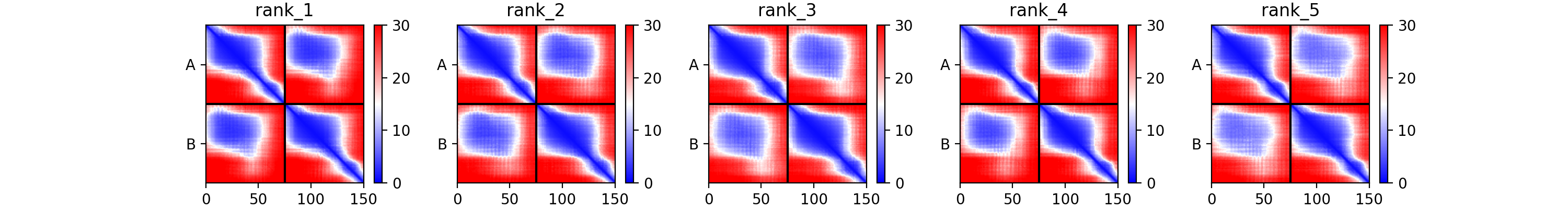

Supplement: Supplementary file 15 — Source Data Fig. 5 [file 44320_2024_19_MOESM15_ESM.zip › Source Data Figure 5/ColabFold/E-E_420f7.result/E_E_420f7_PAE.png]

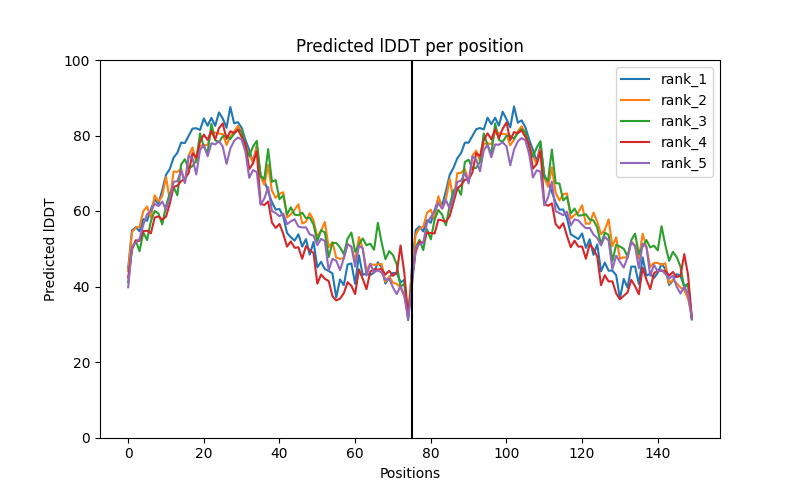

Supplement: Supplementary file 15 — Source Data Fig. 5 [file 44320_2024_19_MOESM15_ESM.zip › Source Data Figure 5/ColabFold/E-E_420f7.result/E_E_420f7_plddt.png]
