# Supplementary figures and images for: AI-guided pipeline for protein–protein interaction drug discovery identifies a SARS-CoV-2 inhibitor (part 2 of 2)
Source: Mol Syst Biol. 2024 Mar 11;20(4):428–57. doi: 10.1038/s44320-024-00019-8 (PMC10987651; doi:10.1038/s44320-024-00019-8)

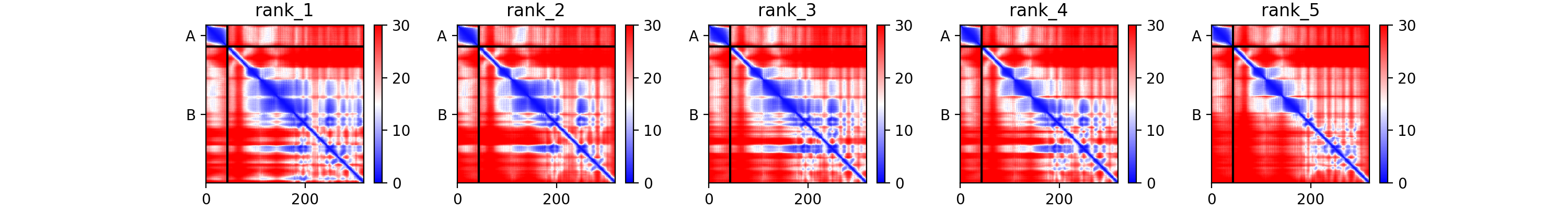

Supplement: Supplementary file 15 — Source Data Fig. 5 [file 44320_2024_19_MOESM15_ESM.zip › Source Data Figure 5/ColabFold/ORF7b-ORF3a_a5d31.result/ORF7b_ORF3a_a5d31_PAE.png]

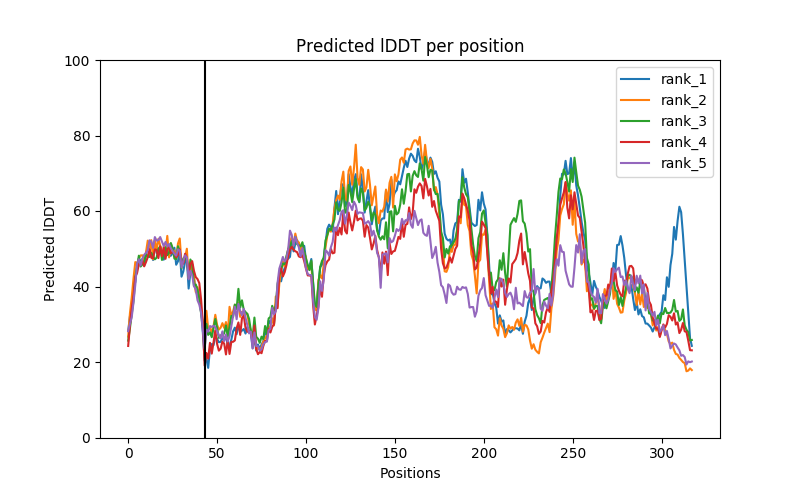

Supplement: Supplementary file 15 — Source Data Fig. 5 [file 44320_2024_19_MOESM15_ESM.zip › Source Data Figure 5/ColabFold/ORF7b-ORF3a_a5d31.result/ORF7b_ORF3a_a5d31_plddt.png]
