# Supplementary material for: AI-guided pipeline for protein–protein interaction drug discovery identifies a SARS-CoV-2 inhibitor
Source: Mol Syst Biol. 2024 Mar 11;20(4):428–57. doi: 10.1038/s44320-024-00019-8 (PMC10987651; doi:10.1038/s44320-024-00019-8)
Supplement: Supplementary file 17 — Expanded View Figures [file 44320_2024_19_MOESM17_ESM.pdf]

Expanded View Figures

Figure EV1. (related to Fig. 1). Effect of different scoring approaches on recovery rates.

(**A**) Schematic overview of the LuTHy-BRET and LuTHy-LuC assays. X: Protein X, Y: Protein Y, D: NanoLuc donor, A: mCitrine acceptor, AB: antibody. (**B**) With the LuTHy assay, each protein pair X–Y can be tested in eight possible configurations (N- vs. C-terminal fusion for each protein), and proteins can be swapped from one tag to the other resulting in 16 quantitative scores for each protein pair, i.e., eight for LuTHy-BRET and eight for LuTHy-LuC. (**C**) Line plots showing the fraction of protein pairs that scored positive (*y* axis) dependent on the quantitative interaction scores (*x* axis) for 10 binary PPI assay versions. For each tested protein pair, the tagging configuration with the highest interaction score is used. For LuTHy all eight tagging configurations were tested, whereas for MN2H, VN2H, YN2H, GPCA, NanoBi four and for KISS, MAPPIT and SIMPL two tagging configurations were tested. Recovery rates at maximum specificity, i.e., where none of the protein pairs in the RRS scored positive (0%), are indicated. Note that in Choi et al (Choi et al, 2019) recovery rates at maximum specificity were calculated by using distinct cutoffs for each tagging configuration. (**D**) Line plots showing the fraction of protein pairs that scored positive (*y* axis) dependent on the distribution of interaction scores, i.e., the mean of all interaction scores + n*(sd) (*x* axis) for 10 binary PPI assay versions. Recovery rates at mean + 1 standard deviation are indicated. (**E**) Line plots showing the fraction of protein pairs that scored positive (*y* axis) dependent on the distribution of interaction scores, i.e., the median of all interaction scores + n*(sd) (*x* axis) for 10 binary PPI assays. Recovery rates at median + 1 standard deviation are indicated. LuTHy experiments from this study were repeated twice with $n = 2$, biological replicates, each containing $n = 3$ technical replicates; SIMPL from Yao et al (Yao et al, 2020); all other from Choi et al (Choi et al, 2019). Note that the SIMPL assay was benchmarked by Yao et al (Yao et al, 2020) against 88 positive proteins pairs derived from the hsPRS-v1 (Venkatesan et al, 2009) and as a random reference set against "88 protein pairs with baits and preys selected from the PRS but used in combinations determined computationally to have low probability of interaction" (Yao et al, 2020).

 © The Author(s)

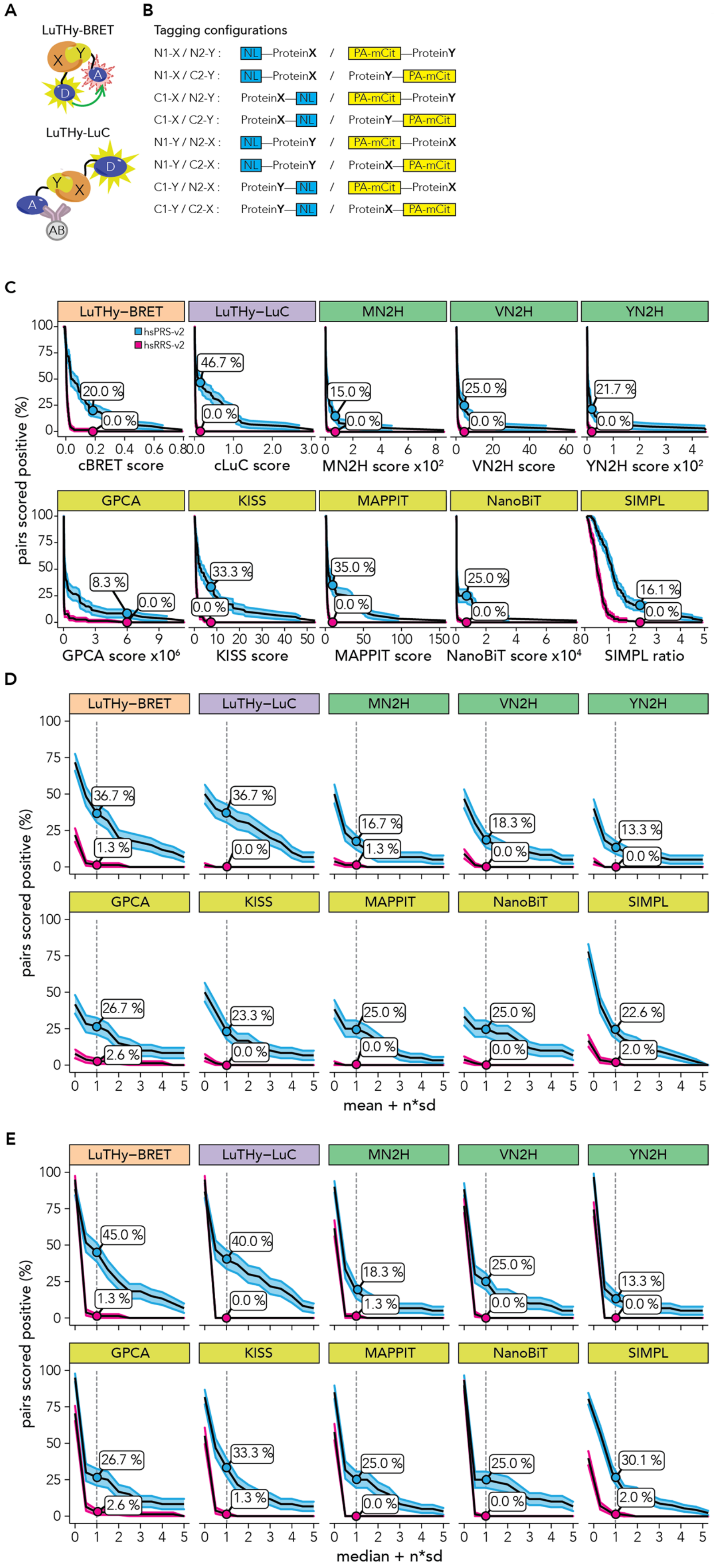

A
LuTHy-BRET
LuTHy-LuC
X
Y
A
D
AB
B
Tagging configurations
N1-X / N2-Y : NL—ProteinX / PA-mCit—ProteinY
N1-X / C2-Y : NL—ProteinX / ProteinY—PA-mCit
C1-X / N2-Y : ProteinX—NL / PA-mCit—ProteinY
C1-X / C2-Y : ProteinX—NL / ProteinY—PA-mCit
N1-Y / N2-X : NL—ProteinY / PA-mCit—ProteinX
N1-Y / C2-X : NL—ProteinY / ProteinX—PA-mCit
C1-Y / N2-X : ProteinY—NL / PA-mCit—ProteinX
C1-Y / C2-X : ProteinY—NL / ProteinX—PA-mCit
C
LuTHy−BRET
LuTHy−LuC
MN2H
VN2H
YN2H
hsPRS-v2
hsRRS-v2
pairs scored positive (%)
cBRET score
cLuC score
MN2H score x10²
VN2H score
YN2H score x10²
GPCA
KISS
MAPPIT
NanoBiT
SIMPL
GPCA score x10⁶
KISS score
MAPPIT score
NanoBiT score x10⁴
SIMPL ratio
20.0 %
0.0 %
46.7 %
0.0 %
15.0 %
0.0 %
25.0 %
0.0 %
21.7 %
0.0 %
8.3 %
0.0 %
33.3 %
0.0 %
35.0 %
0.0 %
25.0 %
0.0 %
16.1 %
0.0 %
D
pairs scored positive (%)
36.7 %
1.3 %
36.7 %
0.0 %
16.7 %
1.3 %
18.3 %
0.0 %
13.3 %
0.0 %
26.7 %
2.6 %
23.3 %
0.0 %
25.0 %
0.0 %
25.0 %
0.0 %
22.6 %
2.0 %
mean + n*sd
E
pairs scored positive (%)
45.0 %
1.3 %
40.0 %
0.0 %
18.3 %
1.3 %
25.0 %
0.0 %
13.3 %
0.0 %
26.7 %
2.6 %
33.3 %
1.3 %
25.0 %
0.0 %
25.0 %
0.0 %
30.1 %
2.0 %
median + n*sd

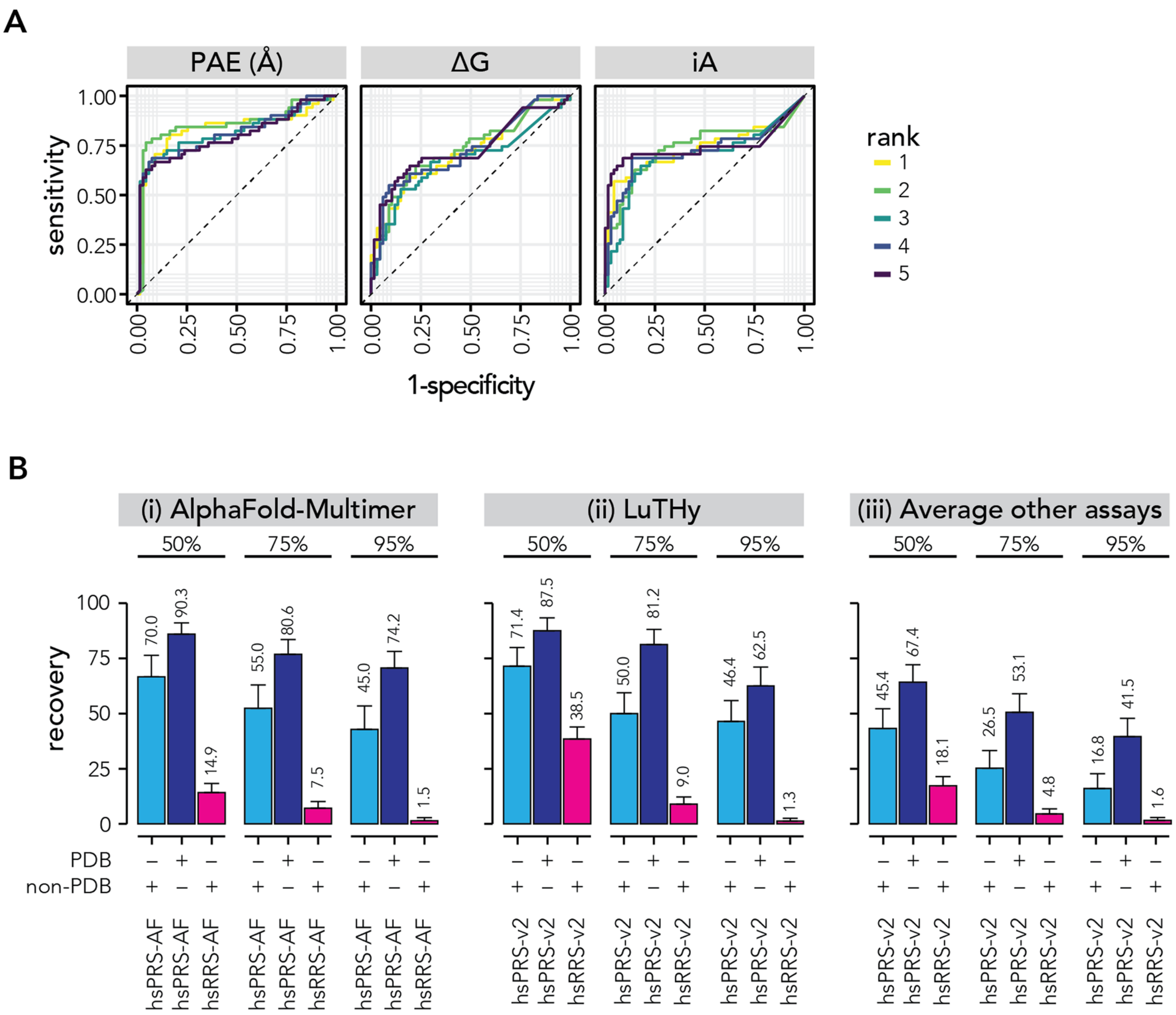

Figure EV2. (related to Fig. 2). Training a maSVM algorithm to classify AFM-predicted structures.

(**A**) Receiver characteristic analysis comparing sensitivity and specificity between the five AFM-predicted structural models for PAE, ΔG and iA of the hsPRS-AF and hsRRS-AF. (**B**) Bar plots showing the fraction of hsPRS-AF and hsRRS-AF interactions with structures deposited in PDB that scored above classifier probabilities of 50%, 75% and 95% by AlphaFold-Multimer (i) by LuTHy (ii) or the mean recovery of N2H (MN2H, VN2H, YN2H), GPCA, KISS, MAPPIT and NanoBiT (iii). Data for the SIMPL assay was excluded for this analysis due to the different composition of the reference sets. LuTHy experiments from this study were repeated two times with $n = 2$, biological replicates, each containing $n = 3$ technical replicates; AFM was used to predict $n = 5$ structural models; all other from Choi et al (Choi et al, 2019). Bars and error bars in this figure represent mean values and standard error of the proportion, respectively.

 © The Author(s)

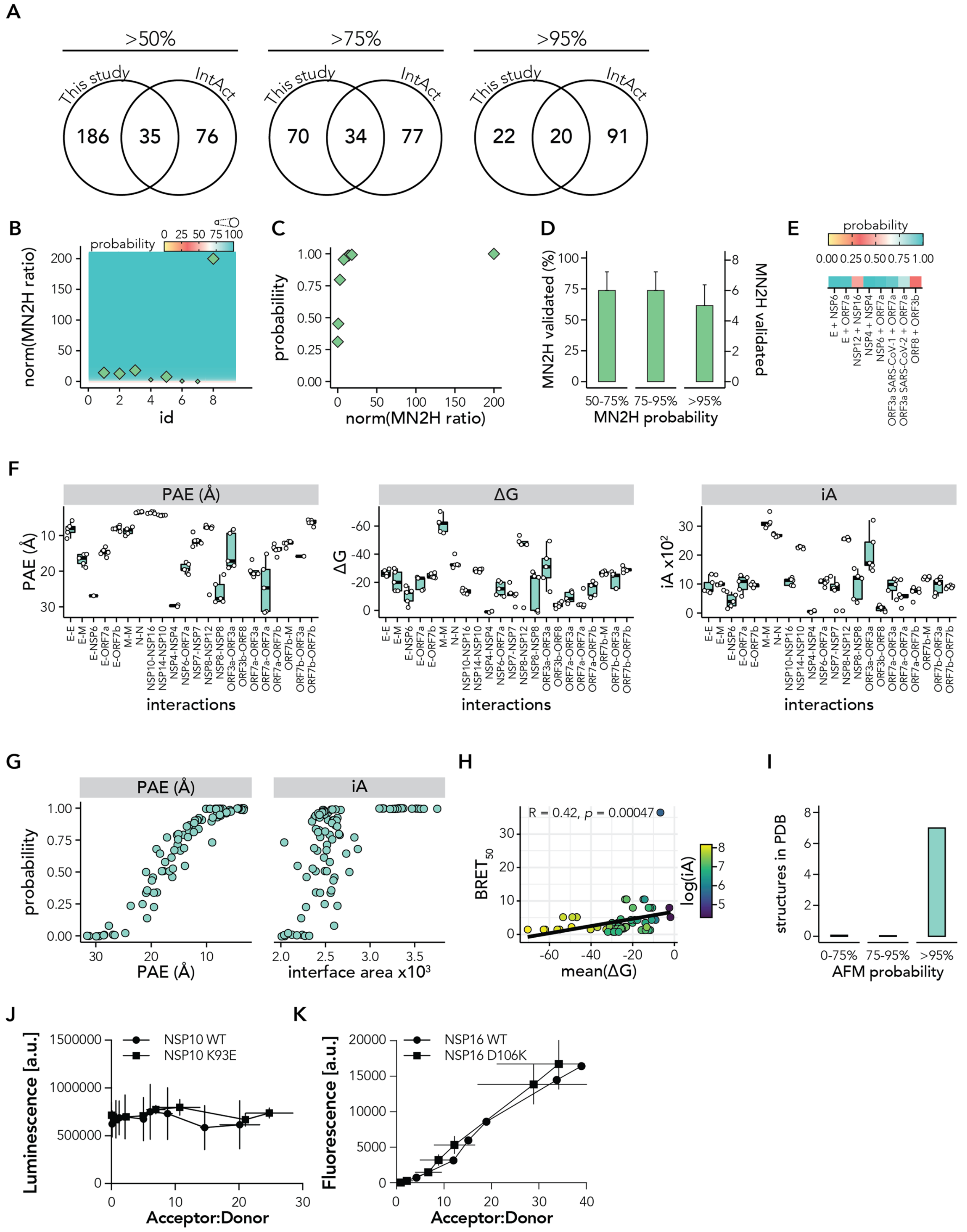
A
>50%
This study
IntAct
186
35
76
>75%
This study
IntAct
70
34
77
>95%
This study
IntAct
22
20
91
B
probability
0 25 50 75 100
norm(MN2H ratio)
id
C
probability
norm(MN2H ratio)
D
MN2H validated (%)
MN2H validated
50-75% 75-95% >95%
MN2H probability
E
probability
0.00 0.25 0.50 0.75 1.00
E + NSP6
E + ORF7a
NSP12 + NSP16
NSP4 + NSP4
NSP6 + ORF7a
ORF3a SARS-CoV-1 + ORF7a
ORF3a SARS-CoV-2 + ORF7a
ORF8 + ORF3b
F
PAE (Å)
ΔG
iA
iA x10²
interactions
E-E
E-M
E-NSP6
E-ORF7a
E-ORF7b
M-M
N-N
NSP10-NSP16
NSP14-NSP10
NSP4-NSP4
NSP6-ORF7a
NSP7-NSP7
NSP8-NSP12
NSP8-NSP8
ORF3a-ORF3a
ORF3b-ORF8
ORF7a-ORF3a
ORF7a-ORF7a
ORF7a-ORF7b
ORF7b-M
ORF7b-ORF3a
ORF7b-ORF7b
G
PAE (Å)
iA
probability
interface area x10³
H
R = 0.42, p = 0.00047
BRET50
log(iA)
mean(ΔG)
I
structures in PDB
0-75% 75-95% >95%
AFM probability
J
NSP10 WT
NSP10 K93E
Luminescence [a.u.]
Acceptor:Donor
K
NSP16 WT
NSP16 D106K
Fluorescence [a.u.]
Acceptor:Donor

© The Author(s)

Figure EV3. (related to Figs. 4 and 5). Validating SARS-CoV-2 protein interactions using the mN2H assay and predicting SARS-CoV-2 protein complexes structures using AlphaFold-Multimer.

(**A**) Venn diagrams showing the overlap between interactions recovered by LuTHy at >50%, >75% and >95% probabilities and interactions deposited in the IntAct database (Orchard et al, 2014). (**B**) Scatter plot showing normalized mN2H ratios (*y* axis) of each of the eight SARS-CoV-2 interactions newly identified with LuTHy (*x* axis). Average classifier probabilities obtained from the hsPRS-v2/hsRRS-v2 mN2H models are displayed as the size of the data points and as a colored grid in the background. (**C**) Scatter plot showing normalized mN2H ratios (*x* axis) against classifier probabilities (*y* axis) for the newly identified SARS-CoV-2 interactions selected for validation. (**D**) Bar plots showing the fraction (left *y* axis) and number (right *y* axis) of newly identified SARS-CoV-2 interactions selected for validation that scored above classifier probabilities of 50%, 75% or 95% with mN2H. Bars and error bars represent mean values and standard error of the proportion, respectively, with $n = 3$ biological replicates. (**E**) Heatmaps showing the mN2H classifier probabilities for the newly identified SARS-CoV-2 interactions selected for validation. (**F**) Boxplots showing predicted alignment error (PAE), solvation-free energy (ΔG) and interface area (iA) from AlphaFold-Multimer (AFM) predicted SARS-CoV-2-AF structures. Boxplots display the median, lower and upper hinges of the 25th and 75th percentiles and lower and upper whiskers extending from the hinges with 1.5× the interquartile range. Each dot represents one predicted structural model. (**G**) Scatter plot showing PAE (*x* axis) against interface area (*y* axis) for all SARS-CoV-2-AF (orange) protein pairs. Average classifier probability predicted by the 100 maSVM models trained by the hsPRS-AF and hsRRS-AF set (see Fig. 2F), is displayed as the size of the data points. Each point in the colored grid in the background displays the average classifier probabilities from the 100 maSVM models. (**H**) Scatter plot showing the ΔG (*x* axis) for all five AFM-predicted structural SARS-CoV-2-AF models against the LuTHy-BRET determined binding strengths ($BRET_{50}$, see Appendix Fig. S7A,B). The respective log-transformed interface areas are indicated by the fill color of the data points. A linear regression fit through the data is shown and the Spearman correlation coefficient (R) and *P* value are indicated. (**I**) Barplot showing the fraction of AFM-predicted structures with 0–75%, 75–95% and >95% classification probability that have an experimentally reported structure deposited to the PDB (Berman et al, 2000) database. (**J**,**K**) Luminescence (**J**) and fluorescence (**K**) values from LuTHy-BRET donor saturation experiments, where constant amounts of NSP10-NL WT or K93E (Lys93Glu) are co-expressed with increasing amounts of mCitrine-NSP16 WT or D106K (Asp106Lys). Experiments with NSP10-NL WT and K93E were repeated two times, with $n = 2$, biological replicates, and each with $n = 2$ technical replicates; experiments with mCit-NSP16 WT and D106K were repeated four times, with $n = 4$, biological replicates, and each with two technical replicates, $n = 2$. Bars and error bars represent the mean and standard deviation, respectively.

 © The Author(s)
